# Downregulation of transposable elements extends lifespan in *Caenorhabditis elegans*

**DOI:** 10.1038/s41467-023-40957-9

**Published:** 2023-08-29

**Authors:** Ádám Sturm, Éva Saskői, Bernadette Hotzi, Anna Tarnóci, János Barna, Ferenc Bodnár, Himani Sharma, Tibor Kovács, Eszter Ari, Nóra Weinhardt, Csaba Kerepesi, András Perczel, Zoltán Ivics, Tibor Vellai

**Affiliations:** 1 Department of Genetics, Eötvös Loránd University (ELTE), 1117 Budapest, Hungary; 2 Eötvös Loránd Research Network (ELKH)-ELTE Genetics Research Group, 1117 Budapest, Hungary; 3 HCEMM-BRC Metabolic Systems Biology Research Group, 6726 Szeged, Hungary; 4 Synthetic and Systems Biology Unit, Institute of Biochemistry, Biological Research Centre, Eötvös Loránd Research Network (ELKH), Temesvári krt. 62, 6726 Szeged, Hungary; 5 Institute for Computer Science and Control (SZTAKI), 1111 Budapest, Hungary; 6 Brigham and Women’s Hospital & Harvard Medical School, Boston, MA 02115 USA; 7 Laboratory of Structural Chemistry and Biology & Hungarian Academy of Sciences (MTA)-ELTE Protein Modelling Research Group, Institute of Chemistry, Eötvös Loránd University, 1117 Budapest, Hungary; 8 Division of Medical Biotechnology, Paul Ehrlich Institute, 63225 Langen, Germany; 9 Vellab Biotech Ltd., 6722 Szeged, Hungary

**Keywords:** DNA methylation, Transposition

## Abstract

Mobility of transposable elements (TEs) frequently leads to insertional mutations in functional DNA regions. In the potentially immortal germline, TEs are effectively suppressed by the Piwi-piRNA pathway. However, in the genomes of ageing somatic cells lacking the effects of the pathway, TEs become increasingly mobile during the adult lifespan, and their activity is associated with genomic instability. Whether the progressively increasing mobilization of TEs is a cause or a consequence of ageing remains a fundamental problem in biology. Here we show that in the nematode *Caenorhabditis elegans*, the downregulation of active TE families extends lifespan. Ectopic activation of Piwi proteins in the soma also promotes longevity. Furthermore, DNA *N*^*6*^-adenine methylation at TE stretches gradually rises with age, and this epigenetic modification elevates their transcription as the animal ages. These results indicate that TEs represent a novel genetic determinant of ageing, and that *N*^*6*^-adenine methylation plays a pivotal role in ageing control.

## Abstract (web summary)

Transposable elements in somatic cells become increasingly mobile during ageing. Here, the authors show that in the nematode *Caenorhabditis elegans*, downregulation of transposable elements extends lifespan, and that their increases with age are coupled with progressively growing *N*^*6*^-adenine methylation in these genetic loci.

## Introduction

Transposable elements (TEs), also called mobile genetic elements or jumping genes, are typically 0.1–20 kilobase-long DNA stretches that can change their genomic position, causing insertional mutations in the new locations^1,2^. If the acceptor chromosomal site contains functional—coding or regulatory—sequences, TE insertion often leads to a phenotypic consequence. TEs are mainly classified as relocating DNA transposons (the element is excised from its original chromosomal site and moves elsewhere in the genome) and self-replicating retrotransposons (the element is first copied by reverse transcription and then the duplicate jumps into a novel locus)^2^. Several TEs have lost the ability to mobilize themselves through accumulating inactivating mutations during evolution. These so-called non-autonomous elements however can be mobilized by the products of intact TEs determined as autonomous elements. The functional significance of TEs is reflected by the fact that they constitute a large fraction of eukaryotic genomes, the majority of them are represented by retrotransposons, and a significant portion of them is mobile in somatic cells during lifespan. For example, at least 47% of the human genome consists of TE-related repetitive sequences, 97% of these elements are identified as retrotransposons, among which several thousand copies are estimated to be transcriptionally active^3–5^. Although most human retrotransposons are unable to actively jump^4^, both autonomous and non-autonomous elements are capable of causing insertional mutations, which can often lead to cell death^6–8^. This mutagenic activity of TEs is associated with genomic instability^6,9^, which is a hallmark of essentially all ageing cells^10^.

A specific RNA silencing mechanism that protects eukaryotic genomes from the adverse mutagenic activity of TEs is the Piwi-piRNA (P element-induced wimpy testis in *Drosophila*-Piwi-interacting RNA) pathway^11^, which operates in non-ageing cells including germline and cancer stem cells, as well as stem cell-like somatic cells of certain animal species, such as the freshwater hydra and flatworm planaria, but not in ageing somatic cells (the non-ageing cells share a unique feature, the capacity to proliferate indefinitely, while the ageing cells, owing to progressively accumulating cellular damage, undergo a functional decline called senescence and eventually die)^12^. This raises the possibility that TEs may substantially contribute to the ageing process^13–16^. Indeed, accumulating evidence indicates that TEs exhibit a gradually increasing activity during the lifespan of various eukaryotic species^17–24^. Despite these data it is still unclear whether ageing is a cause or a consequence of TE mobility. In other words, whether TEs become progressively active in the soma because the organism ages or, conversely, ageing is a result of their growing mobilization remains unresolved (in fact, there is indication for both in the literature^11,22,24^). Since active TE families consist of large copy numbers of elements in eukaryotic genomes, the simultaneous, genetic inhibition of all members within a TE family by mutations or gene silencing has not yet been achieved, leaving their potential role in the ageing process unclear. It was recently shown that in mice LINE-1 (long interspersed nucleotide element; L1) retrotransposons become transcriptionally derepressed during cellular senescence, and that inhibitors of L1 reverse transcriptase interfere with age-associated inflammation^25^. A similar positive effect of reverse transcriptase inhibitors on longevity was previously observed in *Drosophila*^22^. These studies using pharmacological interventions to modulate TE activity suggest that mobilization of retrotransposons represents a causative factor for ageing. However, it is also possible that inhibitors used in these studies are not specific to reverse transcriptase enzymes and (an)other factor(s) may exert the detected positive effect on senescence and lifespan.

In the present study, we effectively downregulated specific TE families in the nematode *Caenorhabditis elegans*, the compact genome of which contains only a relatively small fraction (<12%) of TE-derived repetitive sequences represented by DNA transposons almost exclusively (Supplementary Fig. 1), and found that this genetic intervention extends lifespan. We also observed a lifespan advantage in transgenic nematodes over control when Piwi proteins were ectopically expressed from a heat-inducible promoter in somatic cells. We finally showed that adenine nucleobases at active TE stretches become progressively methylated as the organism ages, and this change is associated with an elevated transcriptional activity of TEs. Thus, TEs play an important role in driving the ageing process in nematodes and perhaps in other organisms, revealing a novel function for a large fraction of eukaryotic genomes.

## Results

### Downregulation of active transposable element families from the onset of adulthood extends lifespan

**Fig. 1:**
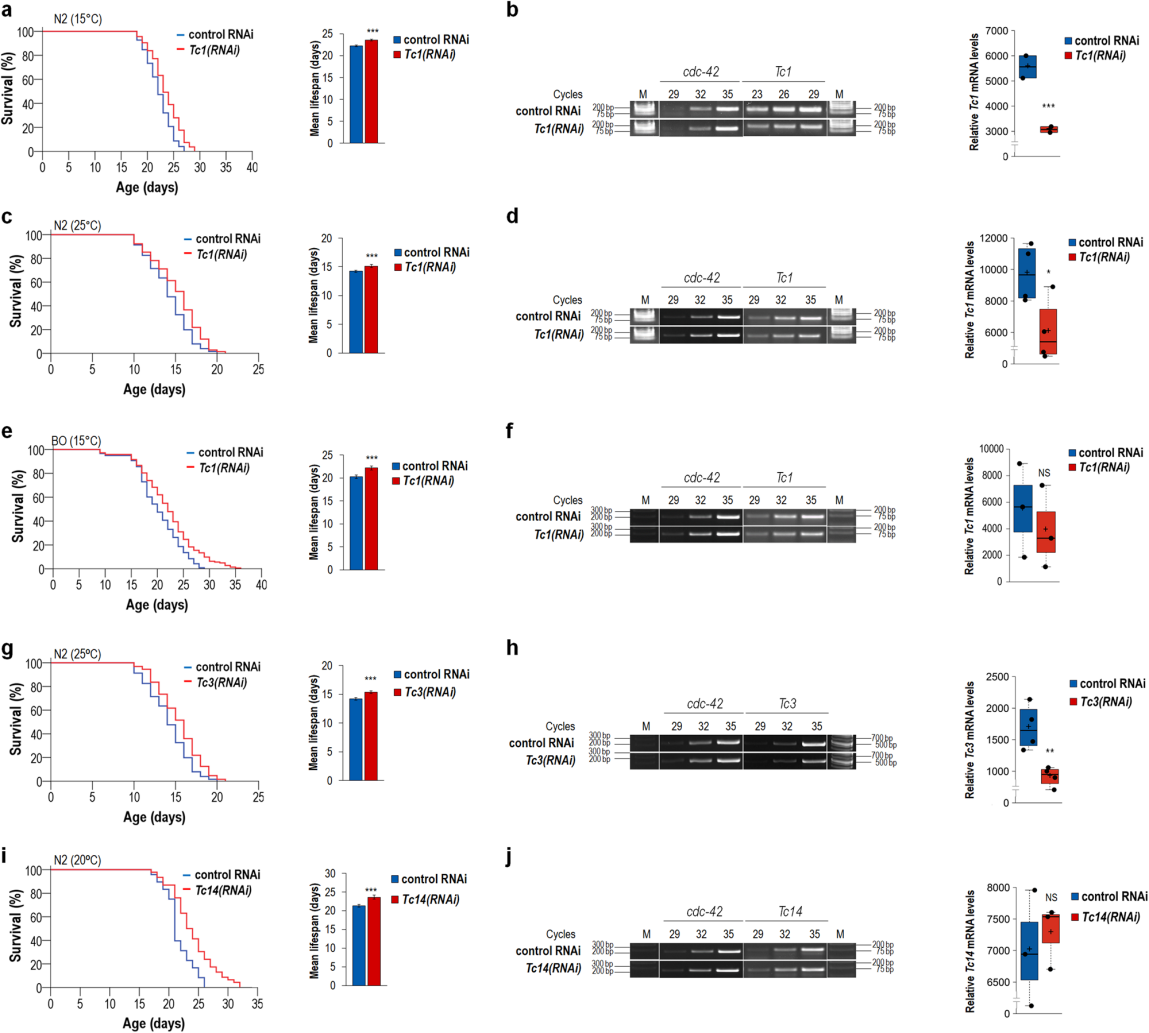
Downregulation of active transposable element (TE) families extends lifespan in *C. elegans*. **a** In the wild-type genetic background (N2), downregulation of *Tc1* (it exists in 31 copies per haploid genome) promotes longevity at 15 °C (left panel). Mean lifespan of control (treated with the empty vector T444T only) versus *Tc1(RNAi)* animals (right panel). **b** Semi-qPCR analysis shows that *Tc1* transcript levels are decreased in the RNAi-treated sample relative to control (left panel). Quantification of PCR bands from three independent experiments (right panel). **c** *Tc1* silencing also extends lifespan at 25 °C (left). Mean lifespan of control and *Tc1(RNAi)* animals (right). **d** Treating animals with double-stranded RNA specific to *Tc1* lowers the corresponding transcript levels (left). Quantification of transcript levels (right). **e** At 15 °C, depletion of *Tc1* in the mutator strain Bergerac (BO) (*Tc1* exists in over 500 copies per haploid genome in this strain) causes a significant lifespan advantage over its control (left). Mean lifespan of control versus RNAi-treated BO animals (right). **f** *Tc1* transcript levels in control vs. *Tc1(RNAi)* animals (left). Quantification of transcript levels from three independent experiments (right). **g** *Tc3(RNAi)* animals live longer than control at 25 °C (left). Mean lifespan of control versus *Tc3(RNAi)* animals (right). **h** RNAi treatment reduced *Tc3* transcript levels by nearly half (left). Quantification of band c (right). **i** Silencing of *Tc14* confers a lifespan advantage over control at 20 °C (left). Mean lifespan of control versus RNAi-treated animals (right). **j** Transcript levels of control versus *Tc14(RNAi)* animals (left). Quantities of PCR bands (right). In panels (**a**, **c**, **e**, **g** and **i**), Kaplan–Meier lifespan curves are shown (left), bars indicate ±S.E.M; *P < 0.05, **P < 0.01, ***P < 0.001, NS: not significant; independent two-sample Student’s two-sided *t* test with Bonferroni correction (right). For statistics, see Supplementary Tables 1 and 2. In panels (**b**, **d**, **f**, **h** and **j**) (left), M indicates molecular weight marker (sizes shown in base pair), and *cdc-42* (cell division cycle-related) was used as an internal control. Total RNA samples were isolated at early adult stages (day 1-2). In the dot plot figures (right), centre lines show the medians; box limits indicate the 25th and 75th percentiles as determined by R software; whiskers extend 1.5 times the interquartile range from the 25th and 75th percentiles, outliers are represented by dots; crosses represent sample means; data points are plotted as circles. Temperatures at which assays were performed are indicated. RNAi treatments were initiated from the onset of adulthood only.

**Fig. 2:**
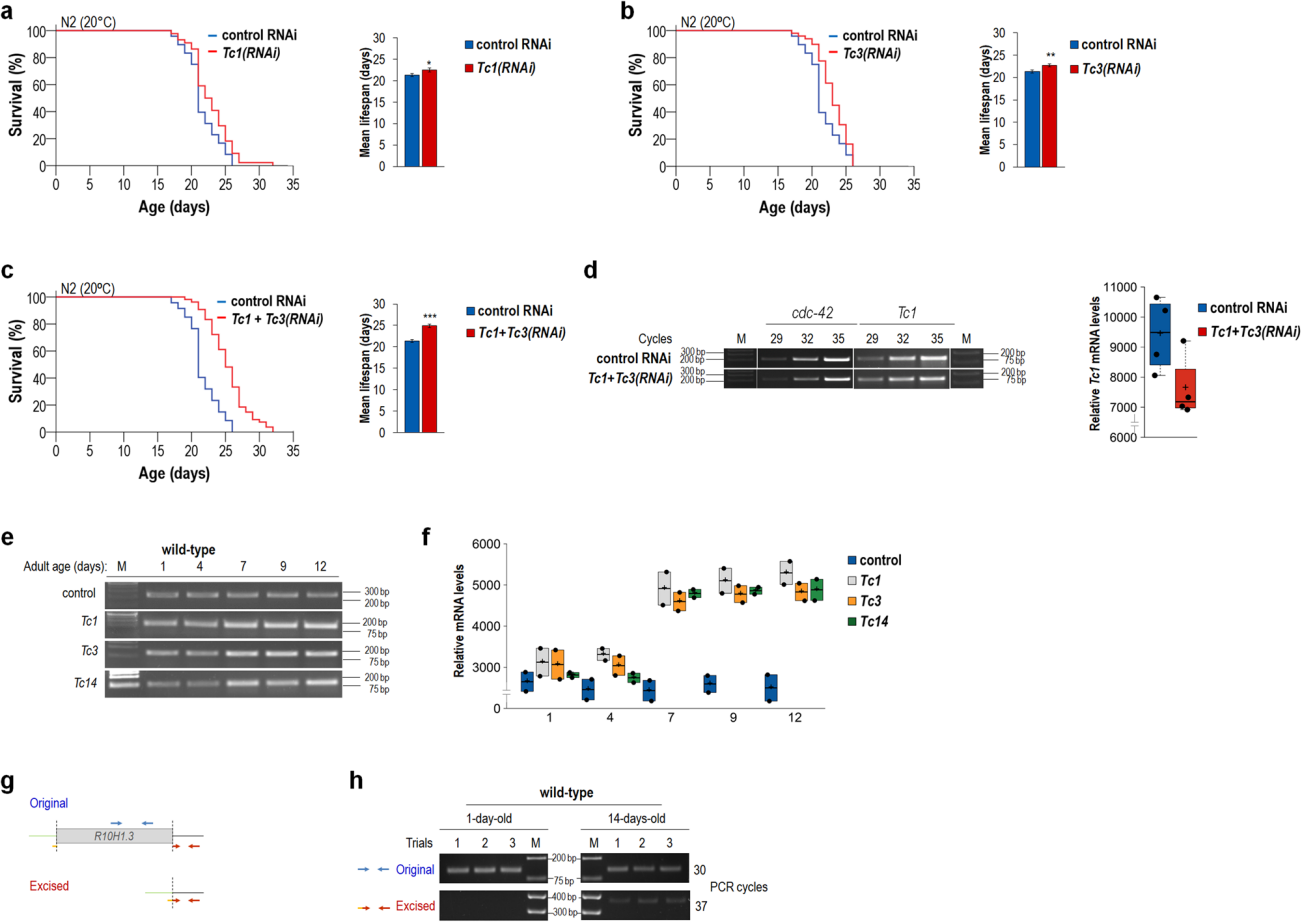
Simultaneous downregulation of *Tc1* and *Tc3* increases lifespan additively. **a** Knock down of *Tc1* in the wild-type genetic background (N2) extends lifespan at 20 °C (left panel). Mean lifespan of control and *Tc1(RNAi)* animals under this condition (right panel). **b** Animals treated with dsRNA specific to *Tc3* live longer than control at 20 °C (left). Mean lifespan of control versus *Tc3(RNAi)* animals (right). **c** At 20 °C, parallel downregulation of *Tc1* and *Tc3* causes a more significant lifespan extension than either single RNAi treatment alone (left). Mean lifespan of control versus *Tc1* + *Tc3(RNAi)* animals (right). **d** Semi-qPCR analysis showing relative *Tc1* transcript levels in control and *Tc1* + *Tc3(RNAi)* animals (left). *cdc-42* was used as an internal control. M indicates molecular weight marker (fragment sizes are shown in base pairs). Relative quantities of *Tc1* transcript levels in control versus *Tc1* + *Tc3(RNAi)* animals (right). Average levels from three independent experiments. Control animals were treated with bacteria expressing the empty RNAi vector T444T only. Kaplan–Meier lifespan curves are shown (**a-c**, left). Bars indicate ±S.E.M, *P < 0.05, **P < 0.01, ***P < 0.001, independent two-sample Student’s two-sided *t* test with Bonferroni correction (right). For statistics, see Supplementary Tables 1 and 2. **e** Semi-qPCR analysis showing relative transcript levels of *Tc1*, *Tc3* and *Tc14* in the wild-type genetic background at different stages of adulthood. M: molecular weight marker. **f** Quantification of band intensities from 3 independent experiments. Dot plot figures: centre lines show the medians; box limits indicate the 25th and 75th percentiles as determined by R software; whiskers extend 1.5 times the interquartile range from the 25th and 75th percentiles, outliers are represented by dots; crosses represent sample means; data points are plotted as circles (**d** and **f**, right). **g** Experimental design for monitoring the excision of an individual *Tc3* stretch, *R10H1.3*. A control primer pair (blue arrows) is designed for detecting the intact *Tc3* internal coding sequence at the original chromosomal site (irrespective of transposition). Another primer pair (red arrows) is used to detect excision. First half of the forward primer is specific to sequences that flank the upstream end of *R10H1.3*, while the other half is specific to sequences flanking the downstream end of *R10H1.3*. So, the primer can work only when the element was excised and the flanking sequences got juxtaposed by DNA repair. **h** Excision of *R10H1.3* at young (1-day-old) and advanced (14-day-old) adult stages. At the adult stage of day 1, excision cannot be detected (the intact element is visible). At day 14, the amounts of fragments excised are well-visible (three independent trials). M, molecule weight marker.

The *C. elegans* genome encodes several active DNA transposon families, in which the elements are present in relatively small copy numbers (Supplementary Fig. 1)^26^. For example, the haploid genome of *C. elegans* contains 31 *Tc1* (transposon *C. elegans* number 1) and 22 *Tc3* DNA transposon stretches, which are the most mobile TEs in this organism^26^. The limited copy numbers of active TE families in nematodes raise the possibility that individual TE families can be effectively downregulated and this genetic intervention may confer a longevity advantage. First, we used a recently developed, highly effective RNA interference (RNAi) method^27^ to silence *Tc1* elements in an otherwise wild-type genetic background. The treatment of animals with *Tc1*-specific double-stranded (ds) RNA was initiated from the onset of adulthood only (non-gravid adult hermaphrodites were transferred onto RNAi plates) because some so-called domesticated TE families have been reported to possess essential developmental roles^28^. Animals depleted for *Tc1* (dsRNA was targeted for the transposase coding region) lived longer at each temperature examined (15, 20 and 25 °C) than control nematodes treated with the empty RNAi vector only (Figs. 1a, c and 2a, b, and Supplementary Fig. 2a and Supplementary Table 1). A similar longevity effect was also obtained when animals were treated under conditions lacking FuDR (5-Fluoro-2′-deoxyuridine), a compound that prevents DNA replication, thereby conferring the animal sterile (Supplementary Fig. 2b and Supplementary Table 1). Thus, lifespan extension provided by *Tc1* downregulation was due to a lower activity of *Tc1* elements. The mutator strain Bergerac (BO) contains a much higher copy number of *Tc1* (over 500 copies per haploid genome) than the wild-type N2 strain^29^. Treating BO nematodes with dsRNA specific to *Tc1* also caused a significant lifespan extension over control (Fig. 1e and Supplementary Table 1). This experiment was performed at 15 °C since BO animals become highly sterile and their physiology altered above this temperature^30^. We next downregulated *Tc3* transposase expression to ascertain whether this intervention also promotes longevity. Indeed, the lifespan of *Tc3(RNAi)* animals was longer than that of control under conditions with and without FuDR (Fig. 1g, and Supplementary Fig. 2c, d and Supplementary Table 1). Consistent with these data, somatic overexpression of *Tc3* from a heat-inducible promoter markedly shortened the lifespan of transgenic animals under inducible conditions (Supplementary Fig. 2e, f and Supplementary Table 1). Then, we targeted *Tc14/CemaT1*, another active TE family, for downregulation, and observed that treated animals are long-lived relative to control (Fig. 1i, and Supplementary Fig. 2g and Supplementary Table 1). We also revealed that, except for *Tc14* that may be downregulated at the protein level as it happens to many genes in this organism or, alternatively, becomes active at later adult stages only (total RNA samples were isolated at the adult stage of day 2), silencing of these TEs leads to a significant but not complete reduction in the corresponding transcript levels, thereby supporting the effectiveness of treatments (Fig. 1b, d, f, h, j and Supplementary Table 2). *Cele14* represents a high copy number (~2000 per haploid genome) TE family reported to have no somatic activity^31^, and its downregulation did not modulate lifespan in the treated animals (Supplementary Fig. 2h and Supplementary Table 1). *Tc2* (only 4 full copies in the haploid genome), *Tc4* (with no open reading frame and only 5 copies per haploid genome) and *Tc5* (only 4 copies in the haploid genome which are inactive in the wild type) were also tested but their silencing, as expected, caused no detectable impact on lifespan (data not shown). The contribution of active TE families consisting of only a few copies to the ageing process may be under detectable levels. To sum, specific downregulation of active TE families with relatively high copy numbers extends lifespan in *C. elegans*.

We also asked whether the positive effect of inhibiting active TE families on lifespan can act additively. Downregulation of *Tc1* and *Tc3* each lengthened lifespan by around 10% at 20 °C (Fig. 2a, b and Supplementary Table 1). When both of these TE families were simultaneously silenced under the same condition, the lifespan extension of treated animals relative to control was nearly the sum of those observed in the single treatments (Fig. 2c and Supplementary Table 1). In addition, the co-treatment could also reduce *Tc1* transcript levels (Fig. 2d and Supplementary Table 2).

In line with literature data^26^, the lifespan assay results above imply that several TE families should be mobile in somatic cells. This prompted us to quantify transcript levels of *Tc1*, *Tc3* and *Tc14* throughout the adulthood. We found that these TEs are transcriptionally active, and each expressed at significantly higher levels in aged adults than in younger ones (Fig. 2e, f). Parallel with this attempt, we could reconfirm that an individual copy of *Tc3*, *R10H1.3*, becomes mobile during ageing; *R10H1.3* was effectively excised from its original genomic locus in aged (14-day-old) adults but not in young (1-day-old) ones (Fig. 2g, h).

### Ectopic expression of Piwi proteins in the soma promotes longevity

**Fig. 3:**
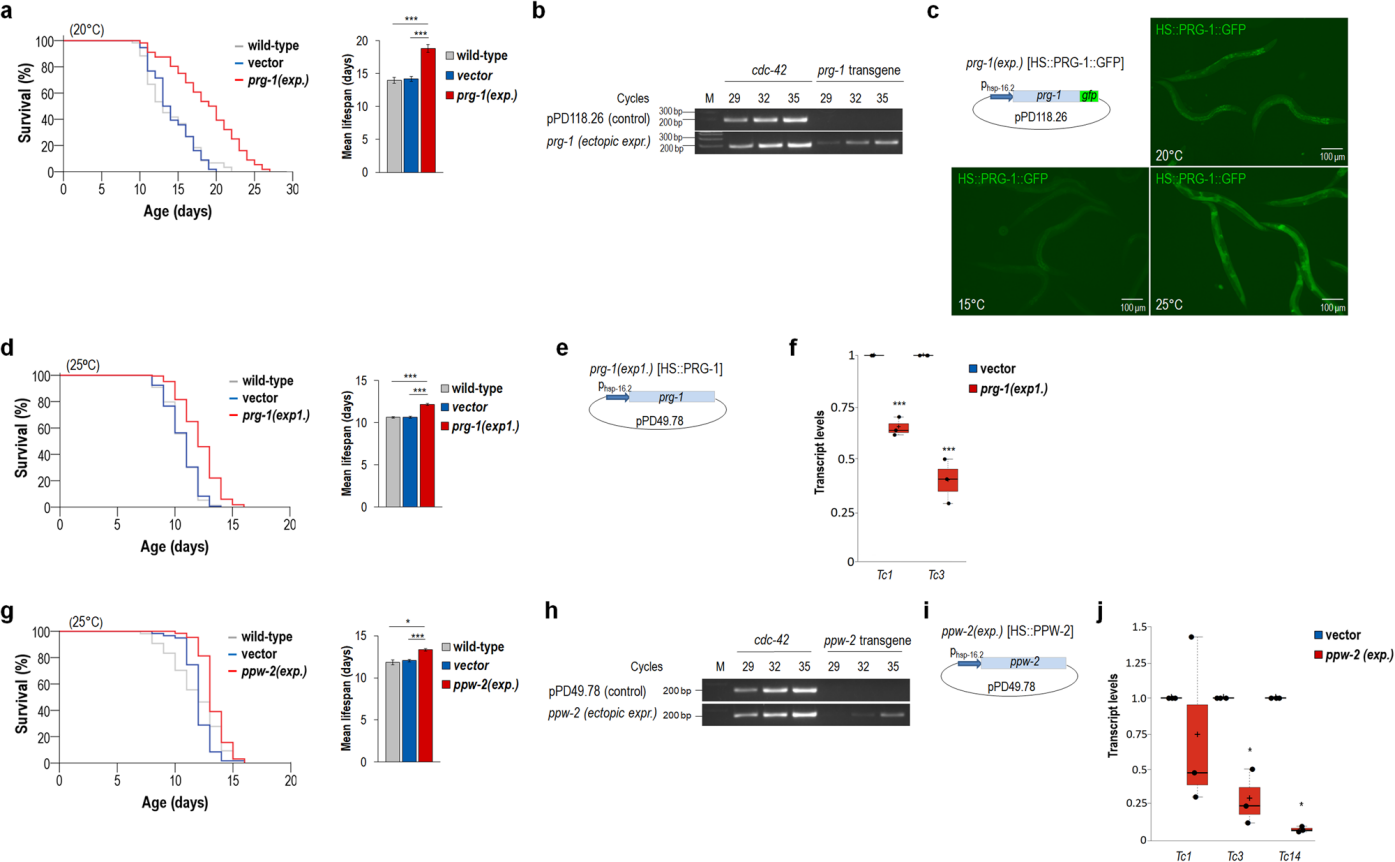
Somatic expression of *prg-1* (Piwi-related gene)*/Piwi* and *ppw-2* (PAZ/PIWI domain containing)*/Ago* each significantly extends lifespan. **a** Expression of a translational fusion PRG-1::GFP reporter gene from a heat-inducible promoter (*hsp-16.2*—heat shock protein) being active in almost all somatic cells markedly increases lifespan at 20 °C (left panel). Mean lifespan of wild-type, control versus PRG-1::GFP transgenic [*prg-1(exp.)*] animals (“vector” indicates animals transgenic for the empty vector pPD118.28) (right panel). **b** Semi-qPCR analysis demonstrating transcript levels of *prg-1* in the soma of transgenic animals (the transgene present in a high copy number is not expressed in the germline, and one of the PCR primers is specific to the vector backbone). M indicates molecule weight marker (numbers show sizes in base pairs). **c** Somatic expression of the PRG-1::GFP fusion protein at 15, 20 and 25 °C. The heat shock promoter is leaky under each of these conditions, so the reporter is expressed even without heat shock (fluorescent signal can be detected). Integrated transgenic lines were analysed. The structure of *prg-1* construct is shown (up-left); *prg-1* coding region is driven by *hsp-16.2* promoter and fused with *gfp* reporter. **d** Another somatically expressed *prg-1* transgene that does not include *gfp* reporter [*prg-1(exp1.)*] leads to a significant lifespan extension at 25 °C (left). Mean lifespan of wild-type, control (transgenic for the empty vector pPD49.78 only) and *prg-1(exp1.)* transgenic animals (right). **e** Structure of *prg-1(exp1.)* transgene: *prg-1* coding region is driven by a *hsp-16.2* promoter. **f** Real-time qPCR analysis showing that transcript levels of both *Tc1* and *Tc3* are lowered in animals transgenic for *prg-1(exp1.)* relative to control (“vector”). **g** Somatic expression of *ppw-2* extends lifespan at 25 °C (left). Mean lifespan of wild-type, control (transgenic for the empty vector) and *ppw-2* transgenic animals (right). **h** Relative transcript levels of *ppw-2* transgene in the soma. M, molecule weight marker. **i** Structure of *ppw-2* transgene; *ppw-2* coding region is driven by a *hsp-16.2* promoter. **j** Real-time qPCR analysis shows that transcript levels of *Tc1*, *Tc3* and *Tc14* are decreased in animals overexpressing *ppw-2* in the soma as compared with control. Kaplan–Meier lifespan curves are shown (**a**, **d**, **g**—left). *exp*. denotes expression. Bars represent ±S.E.M.; *P < 0.05, ***P < 0.001; independent two-sample Student’s two-sided *t* tests (**a**, **d**, **g**—righ*t*, and **f**, **j**) with Bonferroni correction (**a**, **d**, **g**—right) and M**a**nn–Whitney *U* test (**f**, **j**). For statistics, see Supplementary Tables 1 and 3. *cdc-42* was used as an internal control (**b,** **h**). Dot plot figures: centre lines show the medians; box limits indicate the 25th and 75th percentiles as determined by R software; whiskers extend 1.5 times the interquartile range from the 25th and 75th percentiles, outliers are represented by dots; crosses represent sample means; data points are plotted as circles (**f**, **j**).

To further investigate the effect of TE expression on lifespan, we ectopically expressed certain components of the Piwi-piRNA pathway in the soma (Fig. 3, and Supplementary Figs. 3 and 4, and Supplementary Tables 1, 3). Piwi proteins by themselves are capable of degrading TE transcripts^32^, and several members of the pathway, including TE-derived piRNAs, are also expressed somatically (Supplementary Fig. 3)^33^. Hence, it is possible that expressing individual members of the pathway in somatic cells is capable of interfering with TE activity. Indeed, we observed that lifespan is markedly (by around 30%) extended at 20 °C as compared with control when *prg-1* (Piwi-related gene) encoding the primary nematode Piwi protein^34^ is expressed from a heat-inducible promoter, which is active in the majority of somatic cells and leaky even under non-inducing conditions (Fig. 3a and Supplementary Table 1). In these transgenic animals, somatically expressed PRG-1 was fused with GFP (green fluorescent protein), forming a translational fusion reporter system, *p*_*hsp16.2*_::PRG-1::GFP (Fig. 3b, c). Thus, we also generated and tested a *gfp* reporter-free, *p*_*hsp16.2*_-driven, functional *prg-1* transgene (*p*_*hsp16.2*_::PRG-1). Transgenic animals expressing endogenous PRG-1 proteins in somatic cells lived significantly longer (by around 20% at 25 °C) than controls (wild-type worms and nematodes transgenic for the empty vector) at both 15 and 25 °C (Fig. 3d–f, and Supplementary Fig. 4a, b and Supplementary Table 1). Somatic expression of *prg-1* from this construct strongly lowered transcript levels of *Tc1* and *Tc3*, as revealed by a coupled RT-qPCR analysis (Fig. 3f and Supplementary Table 3), and effectively elevated *prg-1* transcript levels under inducing conditions (Supplementary Fig. 5). Furthermore, we found that simultaneous downregulation of *Tc1* and *Tc3* in transgenic animals expressing PRG-1 somatically is incapable of further increasing lifespan (Supplementary Fig. 6 and Supplementary Table 1). Thus, somatic PRG-1 is likely to extend lifespan by inhibiting TE expression.

The somatic accumulation of PRG-2, another Piwi paralogue with unknown function in TE inhibition, also increased lifespan (Supplementary Fig. 4c, d and Supplementary Table 1) (we should emphasize that although *prg-2* is predicted as a pseudogene, it is expressed in the germline and displays a functional redundancy with *prg-1*; see wormbase.org). We then overexpressed *ppw-2* (Paz/Piwi domain-containing) in the soma which is an Argonaute-like protein functioning in the piRNA system (Supplementary Fig. 3)^35^. This genetic intervention conferred a lifespan advantage over control at 25 °C (Fig. 3g, and Supplementary Fig. 4e and Supplementary Table 1) and increased *ppw-2* transcript levels (Fig. 3h, i). Transcriptional activity of *Tc1*, *Tc3* and *Tc14* was reduced in transgenic animals that express *ppw-2* somatically, as compared with control (Fig. 3j and Supplementary Table 3). Based on these results, we conclude that somatic expression of Piwi proteins and certain other components of the Piwi-piRNA pathway attenuates the rate of the ageing process, and this effect is linked to lowered TE expression.

### Adenine nucleobases at active transposable elements are more methylated in aged nematodes than in young adults

It has recently been demonstrated that adenine nucleobases in DNA can be methylated not only in bacteria and plants but in divergent animal taxa ranging from nematodes to mammals^36–39^, and that in *Drosophila* this epigenetic mark preferentially occurs at TE sequences^37^. In addition, *N*^*6*^-methyladenine (6 mA) deposition on certain TE families was reported to affect their activity differently in *Drosophila* (it is associated with activation) and mammals (it is associated with silencing)^37,39^, and was not investigated in nematodes. Therefore, we planned to test the effect of DNA *N*^*6*^-adenine methylation on the transcriptional activity of TEs in *C. elegans*.

**Fig. 4:**
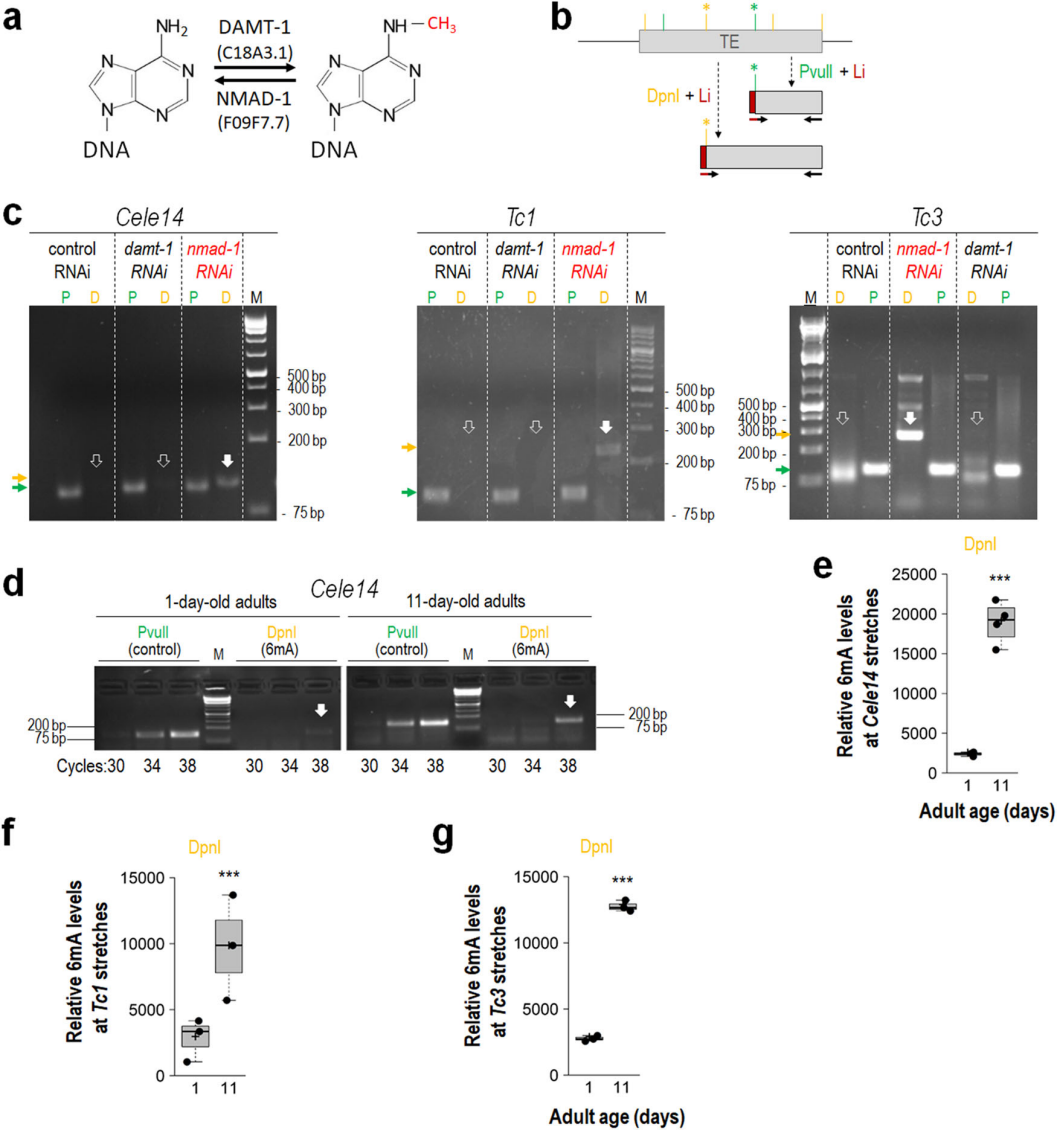
*N*^*6*^-methyladenine levels at active transposable elements increase with age. **a** In *C. elegans*, *N*^*6*^-adenine methylation is catalysed by the putative DNA *N*^*6*^-adenine methyltransferase enzyme DAMT-1 (DNA *N*^*6*^-adenine methyltransferase)/C18A3.1, whereas demethylation of *N*^*6*^-methyladenine (6mA) is mediated by NMAD-1 (a DNA *N*^*6*^-methyladenine demethylase)/F09F7.7. **b** Model showing how 6 mA levels were detected at a specific TE by a PCR-based method developed in this study (see also Supplementary Fig. 7). The restriction enzyme DpnI cuts DNA at a given GATC sequence only when the adenine (A) is methylated. Orange lines: DpnI sites; green lines: PvuII sites (for internal control showing the quantity of the template DNA); stars indicate the target sites. A linker fragment (purple, Li) containing nucleotides specific to an upstream part of the forward primer used for a subsequent PCR reaction is then ligated to the digested genomic fragments. This way the digested (methylated) fragments can be assayed directly (it is crucial since the ratio at which a given adenine nucleobase is methylated among the individual genomes assayed is usually very low). Vertical black and purple-black arrows indicate primers. Forward primer anneals to both linker and flanking genomic sequence. The quantity of the PCR product is normalized to that of control (see Supplementary Figs. 8–10). **c** PCR assay showing that NMAD-1 deficiency causes 6 mA accumulation at *Cele14* (left), *Tc1* (middle) and *Tc3* (right) stretches. Empty/filled thick white arrows: the absence/presence of TE fragments specific to a *N*^*6*^-methylated DpnI site (*); thin horizontal yellow and green arrows: the size of control and 6mA-specific fragments. P: PvuII sites (control); D: DpnI sites (6mA); M: molecule weight marker. **d**–**g** 6mA levels increase with age in TE loci. Semi-qPCR analysis showing relative 6mA levels (DpnI) at *Cele14* stretches in adults at age of days 1 and 11 (**d**). The number of PCR cycles is indicated (bottom). *Tc3*-specific PvuII fragments were used as an internal control. Relative 6mA levels at *Cele14* stretches in young versus aged adults (**e**). Relative 6mA levels (normalized to control) at *Tc1* (**f**) and *Tc3* (**g**) stretches in animals at early (day 1) and advanced (day 11) adult stages. M: molecular weight marker (**c**, **d**). Quantification of relative band intensities (**e**-**g**). Bars represent ±S.E.M; ***P < 0.001; two-sample Student’s two-sided *t* test (**e**–**g**). For statistics, see Supplementary Table 4. Dot plot figures: centre lines show the medians; box limits indicate the 25th and 75th percentiles as determined by R software; whiskers extend 1.5 times the interquartile range from the 25th and 75th percentiles, outliers are represented by dots; crosses represent sample means; data points are plotted as circles (**e**–**g**).

In *C. elegans*, DNA *N*^*6*^-adenine methylation is catalysed by DAMT-1 enzyme (a putative DNA *N*^*6*^-adenine methyltransferase) encoded by the open reading frame *C18A3.1*, while demethylation of 6 mA is achieved by NMAD-1 (*N*^*6*^-methyladenine demethylase) encoded by *F09F7.7* (Fig. 4a)^36,38^. To examine the relationship between age and the rate of *N*^*6*^-adenine methylation in TE loci, we developed a PCR- (polymerase chain reaction) based method to detect relative 6 mA levels at any selected DNA stretch being present in a high copy number in the genome (Fig. 4b, and Supplementary Fig. 7). The method is not only relatively simple, but highly reliable (sequence specific; see Supplementary Fig. 7) unlike the most widely used techniques—including single molecule real-time sequencing (SMRTseq), liquid-chromatography-tandem mass spectrometry (LC-MS/MS) and hybridization with a 6mA-specific antibody—developed to date to identify 6 mA sites in a genome, which have been revealed to frequently generate artefacts^40–43^. In addition, these previous methods are incapable of accurately identifying 6 mA levels at specific genomic sites in individual genomes isolated from a tissue sample. The PCR-based method introduced here is a further development of a previously described technique that involves a DNA methylation-sensitive/dependent enzymatic cleavage of genomic DNA and a subsequent PCR amplification of a selected target (digested) site^44^. Because PCR amplification uses the intact, undigested DNA fragments as template and *N*^*6*^-adenine methylation in general is a very rare event (only a very few individual genomes within a tissue sample are *N*^*6*^-adenine methylated at a given site), the technique is not suitable for accurately determining 6 mA levels at specific DNA sequences. The improved method involves a DpnI-mediated restriction cleavage of genomic DNA samples which is sensitive to the methylated status of the adenine nucleobase found in the restriction site (5′-GA^Me^TC-3′), and a subsequent ligation of an adaptor DNA fragment called linker to the cleaved site, serving as a template for the upstream part of the forward primer that is used in a subsequent PCR reaction (so, the forward primer is simultaneously specific to a downstream part of the linker and the adjacent upstream genomic sequence at the target site; Fig. 4b, and Supplementary Fig. 7). This way the quantity of the digested, methylated DNA fragments at a given site can be determined directly in a tissue sample. Since a given adenine site is randomly *N*^*6*^-methylated among the individual genomes isolated from the tissue sample examined (genomic DNA is isolated from numerous cells), our method can be used to determine precisely relative (as normalized to an internal control) 6 mA levels at the selected sites.

In a control experiment to evaluate our method, we found that three different TEs, *Cele14*, *Tc1* and *Tc3*, display elevated 6mA levels in animals deficient in NMAD-1 function, as compared with the wild-type and DAMT-1 defective backgrounds (Fig. 4c, and Supplementary Fig. 8). Then, relative 6mA levels at these TE sequences were determined in young (1-day-old) versus aged (11-day-old) adults. According to the results, 6mA deposition was markedly increased with age on each TE family examined (Fig. 4d–g, and Supplementary Figs. 9a–c). The increase in relative 6mA levels between young and aged adults was around 5-6-fold in all cases. These data suggest that the epigenetic process DNA *N*^*6*^-adenine methylation influences the mobilization of active TEs during ageing.

### NMAD-1 and DAMT-1 affect the expression of active transposable elements and influence lifespan

**Fig. 5:**
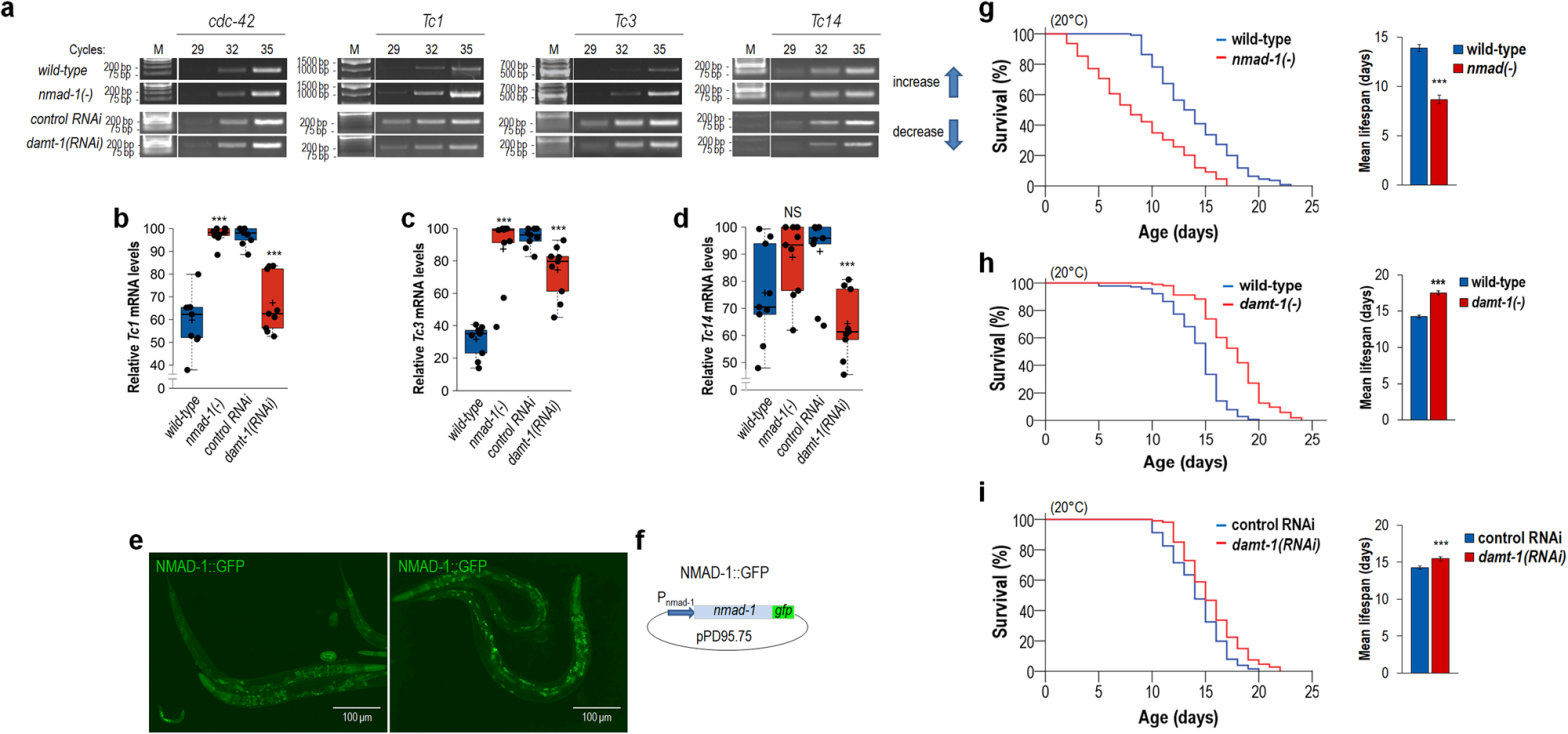
*N*^*6*^-adenine methylation at active transposable elements promotes their expression and accelerates ageing. **a** Transcript levels of *Tc1*, *Tc3* and *Tc14* increase in mutant animals defective for NMAD-1 (*Tc14* displays a tendency), but decrease in animals downregulated for DAMT-1, as compared with the corresponding control (semi-qPCR analysis). *cdc-42* was used as an internal control. The number of PCR cycles is indicated. Transcript levels were tested in 2-day- (NMAD-1 defective) and 6-day-old (DAMT-1 depleted) adults. M indicates molecule weight marker (fragment sizes shown in base pair). **b**–**d** Quantification of relative *Tc1*, *Tc3* and *Tc14* transcript levels (normalized to *cdc-42* transcript levels). Bars indicates ±S.E.M.; ***P < 0.001, NS: not significant; Independent two-sided *t* test. For statistics, see Supplementary Table 2. Dot plot figures: centre lines show the medians; box limits indicate the 25th and 75th percentiles as determined by R software; whiskers extend 1.5 times the interquartile range from the 25th and 75th percentiles, outliers are represented by dots; crosses represent sample means; data points are plotted as circles. **e** Expression of a NMAD-1::GFP translational fusion reporter in various somatic cells. Two representative images of mixed-stage populations are shown. **f** Structure of the reporter construct; a 5 kb-long upstream regulatory fragment and *nmad-1* coding region fused N-terminally with *gfp* gene were cloned into pPD95.75. Integrated transgenic lines were analysed. **g** *nmad-1(−)* mutant animals are short-lived at 20 °C (left panel). Mean lifespan of wild-type and *nmad-1(−)* mutant animals (right panel). Isogenized *nmad-1(ok3133)* mutants were tested. **h** Lifespan curves of wild-type and *damt-1(gk961032)* mutant animal (left). Lifespan advantage conferred by *gk961032* allele is highly significant because the mutation, which is a large deletion, eliminates *damt-1* and four other genes. Knockout of the latter genes is likely to affect survival negatively. The corresponding mean lifespan data (right). **i** Animals depleted for DAMT-1 live longer than control at 20 °C (left). Mean lifespan data of control and *damt-1(RNAi)* animals (right). Kaplan–Meier lifespan curves (**g**–**i**—left). Control animals were fed with bacteria expressing the empty feeding vector only. Bars represent ±S.E.M.; ***P < 0.001; unpaired two-sample Student’s two-sided *t* test with Bonferroni corrections (**g**–**i**—right). For statistics, see Supplementary Table 1.

It was found previously that DNA *N*^*6*^-adenine methylation is positively correlated with TE expression in *Drosophila*^37^ but not in mice^39^. These fly data suggest that this epigenetic modification affects TE activity. To prove that *N*^*6*^-adenine methylation promotes transposition during the adult lifespan, thereby contributing to ageing, we measured relative transcript levels of *Tc1*, *Tc3* (the most active TEs in *C. elegans*) and *Tc14* elements in wild-type versus NMAD-1 defective and in control (wild-type animals fed with the empty RNAi vector only) versus DAMT-1-depleted genetic backgrounds at given adult stages [total RNA samples were isolated at day 2 from wild-type animals and *nmad-1(−)* mutants and at day 6 from RNAi control and *damt-1(RNAi)* animals]. Results we obtained indicate that a loss-of-function mutation in *nmad-1*, *ok3133*, considerably elevates, while downregulation of *damt-1* markedly lowers, the expression of TEs examined relative to the corresponding control (Fig. 5a–d, and Supplementary Fig. 9d–f and Supplementary Table 4). The only exception was *Tc14*, transcript levels of which in the NMAD-1 defective background displayed a tendency to grow but this change was not significant compared to control (Fig. 5a, d). It is possible that *Tc14* becomes active following the early adult stages (day 2) only (in *nmad-1(−)* mutants, total RNA samples were isolated at the adulthood of day 2). Nevertheless, one can conclude that TE expression is promoted by *N*^*6*^-adenine methylation in nematodes. This epigenetic process is likely to increase TE expression in somatic cells in an age-dependent manner. Consistent with this assumption, we found, using an NMAD-1::GFP translational fusion reporter, that *nmad-1* is expressed in essentially all somatic cells during development and throughout adulthood (Fig. 5e, f).

We next determined the lifespan of animals that are defective for either NMAD-1 or DAMT-1. Both *nmad-1(ok3133)* mutants and *nmad-1(RNAi)* nematodes died significantly earlier than the corresponding controls (Fig. 5g, and Supplementary Fig. 10a–c and Supplementary Table 1). In the *nmad-1(ok3133)* mutant background, the expression of *Tc1* and *Tc3* is elevated compared to wild-type (Fig. 5a–c). Thus, lifespan reduction triggered by NMAD-1 deficiency may be due to, at least in part, increased mobilization of *Tc1* and *Tc3*. We showed that simultaneous downregulation of *Tc1* and *Tc3* in *nmad-1(ok3133)* mutant animals significantly increases lifespan as compared with RNAi control [*nmad-1(ok3133)* mutants treated with the empty RNAi vector only] (Supplementary Fig. 10e). In contrast, an inactivating mutation in *damt-1*, *gk961032*, and *damt-1* downregulation each increased lifespan (Fig. 5h, i, and Supplementary Fig. 10d and Supplementary Table 1). Longevity effect conferred by *gk961032* allele was particularly significant because the mutation, which is a large deletion, eliminates *damt-1* and four additional genes from the genome (Supplementary Fig. 10f, g), and these latter genes may have a considerable contribution to viability. Because a previous study reported a slight lifespan reduction in *damt-1(gk961032)* mutant animals^45^, prior to performing this set of lifespan assays, we outcrossed the mutant strain with the wild-type genetic background several times to avoid the effect of possible background mutations, and applied FuDR to suppress germline activity. Under these conditions, the *gk961032* mutation conferred a long-lived phenotype (Fig. 5h, and Supplementary Fig. 10d and Supplementary table 1). Taken together, we propose that DNA *N*^*6*^-adenine methylation contributes to nematode ageing through activating TEs.

### *N*^*6*^-methyladenine deposition on active transposable elements progressively increases with age

**Fig. 6:**
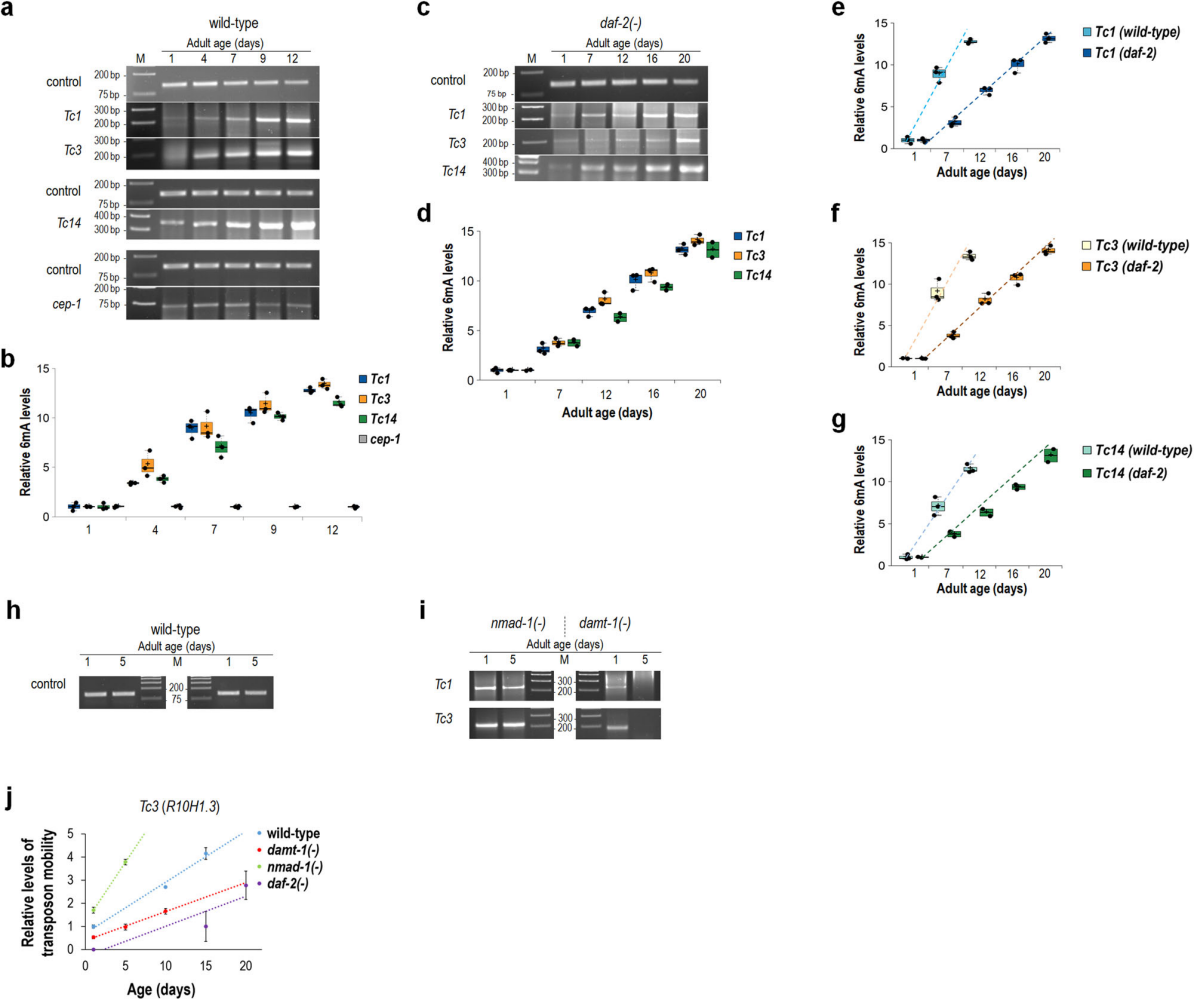
*N*^*6*^-methyladenine levels at active transposable elements gradually increase with age during the adult lifespan. **a** In wild-type animals, 6mA levels in *Tc1*, *Tc3* and *Tc14* loci, but not in *cep-1* (an arbitrary chosen non-TE gene) locus, progressively elevate during ageing. **b** Quantification of relative 6mA levels at TE stretches and *cep-1* site in wild-type adults at different stages. **c** 6mA levels at *Tc1*, *Tc3* and *Tc14* also gradually increase throughout adulthood in a *daf-2(−)* mutant genetic background. **d** Quantification of relative 6mA levels at TE stretches in *daf-2(−)* mutant adults of different ages. **e** Comparison of relative 6mA levels at *Tc1* sequences in wild-type and *daf-2(−)* mutants. In *daf-2(−)* mutants, which live around twice as long as the wild type, 6mA levels elevate with age at a nearly half rate as compared to wild-type. **f** Relative 6mA levels at *Tc3* stretches in wild-type and *daf-2(−)* mutant animals. **g** Relative 6mA levels at *Tc14* sequences in wild-type versus *daf-2(−)* mutant nematodes. The age-dependent growth dynamics of relative 6mA levels at active TE stretches is similar in wild-type and *daf-2(e1370)* mutant animals (**e–g**). The longer lifespan of *daf-2(−)* mutants is associated with a slower rate at which 6mA accumulates at TE stretches. **h** Samples at the 1- and 5-day adult stages contain the same amount of genomic DNA (control). **i** 6mA levels at *Tc1* and *Tc3* stretches are constantly high in *nmad-1(−)* mutants, while remain permanently low in *damt-1(−)* mutants. At day 1, a weak band is still visible in DAMT-1 defective animals, suggesting the presence of another enzyme with DNA *N*^*6*^-adeine methyltransferase activity which acts during development. Isogenized *nmad-1(ok3133)* and *damt-1(gk961032)* mutants were tested. Age (days) is indicated; M, molecular weight marker (numbers show fragment sizes in base pair), control bands indicate relative amounts of genomic DNA (**a**, **c**, **h** and **i**). In panels (**b**, **d**–**g**), bars represent ±S.E.M.; ***P < 0.001; independent two-sided *t* test with Bonferroni correction. For statistics, see Supplementary Tables 1 and 4. Dot plot figures: centre lines show the medians; box limits indicate the 25th and 75th percentiles as determined by R software; whiskers extend 1.5 times the interquartile range from the 25th and 75th percentiles, outliers are represented by dots; crosses represent sample means; data points are plotted as circles. **j** Excision (mobilization) rate of an individual *Tc3* stretch (*R10H1.3*) at different adult stages. The element is more active in *nmad-1(−)* mutants, but less dynamic in *damt-1(−)* mutant, as compared with wild-type. At a given adult day, its excision is the less frequent in *daf-2(−)* mutants among the genotypes tested. Bars indicate ±S.E.M. For statistics, see Supplementary Table 2.

Because adenine nucleobases at active TE stretches are more *N*^*6*^-methylated at advanced ages than at young adulthood (Fig. 4d–g and Supplementary Fig. 9a–c), we next asked whether the rate at which this chromatin modification process takes place is about proportional to the age of the animal. In other words, can the relative *N*^*6*^-methylation level of a given adenine site in a TE locus within a tissue sample be predictive for chronological age? To this end, we determined relative 6 mA levels at *Tc1*, *Tc3* and *Tc14* sequences throughout the adulthood, and found that they do gradually elevate as the organism ages (Fig. 6a, b, and Supplementary Table 4). 6mA levels at *Tc1* stretches were also significantly higher in old adults than in young ones when animals were maintained on agar media without FuDR (Supplementary Fig. 10h). In contrast, *cep-1* (*C. elegans* p53-like protein), which was chosen as a non-TE-like gene encoding the nematode orthologue of mammalian p53, did not exhibit such an age-dependent change in 6mA levels (Fig. 6a, b, and Supplementary Table 4). Rather, relative 6mA levels were nearly constant in the *cep-1* locus during the entire adult lifespan.

We also assessed the age-dependent dynamics of *N*^*6*^-adenine methylation at *Tc1*, *Tc3* and *Tc14* stretches in long-lived *daf-2(−)* (dauer formation defective) mutant animals, which are defective for insulin/IGF-1 (insulin-like growth factor) signalling and live two-times longer than wild-type animals under conditions that permit development into adulthood^46^. Interestingly, the age-dependent rates at which relative 6mA levels increased in these TE loci were highly similar to those found in wild-type animals; 6mA deposition on *Tc1*, *Tc3* and *Tc14* sequences also gradually increased during the adult lifespan but this increment took place about half as fast as in the wild type (Fig. 6c, d, and Supplementary Table 4). These observations suggest that *daf-2(−)* mutant adults, which typically exhibit a lifespan roughly twice that of wild type, display a corresponding delay in reaching the relative maximum levels of 6mA mark at these genomic sites as compared with control (the wild-type background) (Fig. 6e–g).

Because *daf-2(−)* mutant animals live two times longer than wild-type, relative 6mA levels at the end of life (at the maximal lifespan) are about similar in both genetic backgrounds (Fig. 6e–g). Thus, relative 6mA levels in active TE loci appear to correlate to the age of the animal with a given genotype, suggesting *N*^*6*^-adenine methylation as an important process in ageing control. This is consistent with our next observation that there is a significant decrease in TE expression at a given adult stage in *daf-2(−)* mutant animals as compared to the wild type at the same stage (Supplementary Fig. 11 and Supplementary Table 5). In this attempt, we analysed freely available RNAseq data obtained from wild-type and *daf-2(−)* mutant animals at comparable (1-2-day-old) adult stages^47^, and identified 46 TE families, the expression of which were significantly (*P* < 0.05) changed between the two genetic backgrounds. 39 out of these TEs were expressed at significantly lower levels in *daf-2(−)* mutants (Supplementary Fig. 11a and Supplementary Table 5). At this early adult stage, transcript levels of *Tc1* and *Tc3*, but not *Tc14*, were decreased relative to control (*Tc14* may be active at later—mid and advanced—adult stages only) (Supplementary Fig. 11b–d and Supplementary Table 5). We put forward that in a mutant strain living two times longer than the wild type, the cumulative expression of all active TE elements at a certain adult stage (day) is around half the level of that for the wild type at the same stage. Certain TE elements however can be overexpressed in this mutant genetic background relative to wild-type. In sum, 6mA deposition on active, specific TE sequences may serve as an epigenetic signature for determining age from DNA (i.e. a tissue sample).

The effects of DAMT-1, a putative DNA *N*^*6*^-adenine methyltransferase, and NMAD-1 *N*^*6*^-methyladenine demethylase on the methylation status of adenine nucleobases in *Tc1* and *Tc3* loci were also tested at the 1- and 5-day adult stages (Fig. 6h, i). We found that in the *nmad-1(−)* mutant background, relative 6mA levels are equally high at both adult stages. These results imply that low levels of 6mA in these loci at young adult stages is a consequence of NMAD-1 activity. In *damt-1(−)* mutants, however, relative 6mA levels were low but still detectable at the adult day 1 and almost completely absent at day 5 (Fig. 6h, i). Based on these data one can propose that DAMT-1 mediates *N*^*6*^-adenine methylation in these TE loci throughout adulthood, and that during development DNA *N*^*6*^-adenine methylation is also catalysed by (a) yet identified enzyme(s) that is/are distinct from DAMT-1.

We also determined the rate at which a selected individual *Tc3* stretch (*R10H1.3*) is excised from its original genomic site during the adult lifespan in control versus *nmad-1(−)* and *damt-1(−)* mutants, as well as in *daf-2(−)* mutant animals (Fig. 6j). In good agreement with results above (first, relative 6mA levels at active TE sequences increase with age and *N*^*6*^-adenine methylation promotes ageing, and, second, 6 mA accumulates in TE loci at a halved rate in long-lived mutants deficient in DAF-2 activity relative to wild-type), NMAD-1 deficiency strongly increased, while defects in DAMT-1 and DAF-2 function lowered, *Tc3* (*R10H1.3*) transposition as compared with the control genetic background (Fig. 6j, and Supplementary Fig. 12). Thus, the rate of the ageing process is strongly influenced by *N*^*6*^-adenine methylation at active TE stretches.

### Single-molecule real-time sequencing verifies an age-associated increase in *N*^*6*^-methyladenine levels at active transposable elements

**Fig. 7:**
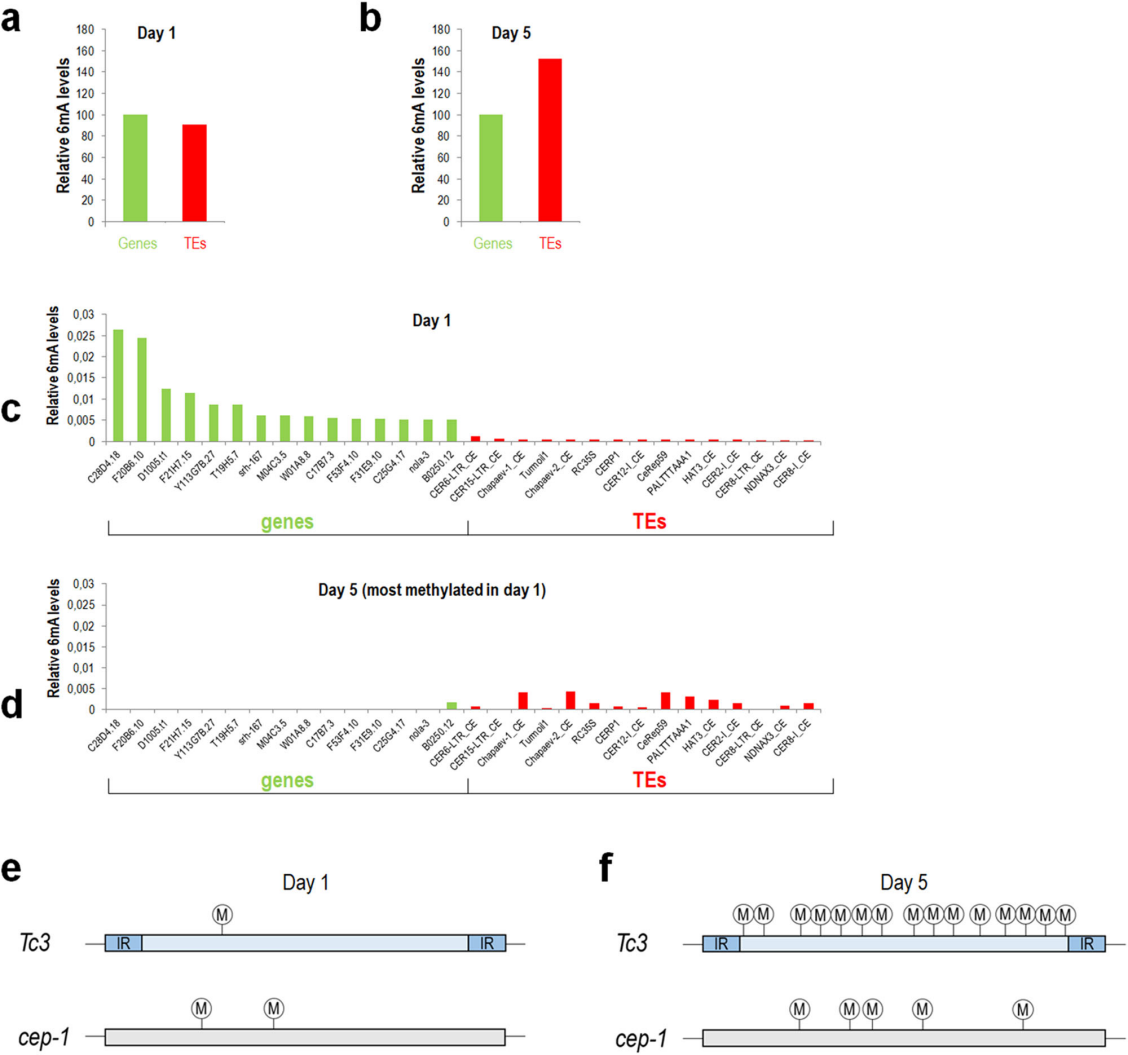
Single molecule real-time sequencing reveals that *N*^*6*^-adenine methylation preferably occurs at transposable elements during adulthood. **a**, **b** Relative 6mA levels at genic and TE sequences in wild-type adults at the 1-day (**a**) and 5-day (**b**) stages. Columns represent the mean of relative 6mA levels at all genes (green) and TE-derived sequences (red), normalized to that of the genes. At the 1-day stage, relative 6mA levels are higher at genic sequences than TE stretches, whereas in 5-day-old adults the ratio is opposite in favour of TEs. 6mA levels were compared between genic and TE sequences within a given stage and not between the stages in order to avoid limitations resulting from different amounts of genomic DNA samples. **c** Relative 6mA levels at the top 15 most methylated genes (green) and TE sequences (red) in 1-day-old adults. 6mA levels are higher at these genes than at the TEs selected. **d** Relative 6mA levels at the same genes and TE stretches in adults at the 5-day stage. TEs display higher 6mA levels than the selected genes do. For TEs, the mean of all copies of a TE family was calculated, and wild-type animals were assayed. **e**, **f** The mean number of 6mA sites at *Tc3* stretches (up) and *cep-1* locus (bottom) in adults at the 1-day (**e**) and 5-day (**f**) stages. The position of 6mA sites is indicated by M in a white circle. In 1-day-old adults, the number of 6mA sites is nearly similar between *Tc3* stretches and *cep-1*, whereas at the 5-day stage *Tc3* stretches contain more *N*^*6*^-methylated adenines than *cep-1* locus.

To validate the above PCR-based data, according to which 6mA deposition on active TE stretches gradually increases with age, we applied SMRTseq technology serving as a direct method currently available to detect chemical modifications of cytosine and adenine nucleobases^48^. Genomic DNA samples isolated from synchronized adult populations at the 1- and 5-day stages were subjected to the analysis. To ensure that the results and conclusions are not influenced by the amount and quality of genomic DNA prepared from the two age groups, that is, variations in library constructions and differences in sequencing coverage, we did not directly compare 6mA sites between 1- and 5-day-old adult animals. Rather, 6mA sites in different loci were identified within a given age group; non-TE-like versus TE sequences were separately assessed at young (1-day) and middle-age (5-day) adult stages (Fig. 7).

Similar to the PCR-based 6 mA results (Fig. 6a, b), SMRTseq data revealed that 6 mA epigenetic marks increase with age at TE sequences relative to non-TE-like genes (Fig. 7a, b). Utilizing the coverage provided by SMRTseq technology, genes and TE sequences were listed and ordered as a function of their relative 6 mA quantity in 1-day-old adults. From these data we extracted how the relative quantity of 6 mA marks on the top 15 most methylated genes and TEs changes during ageing (Fig. 7c, d). We found that the top 15 most methylated genes in 1-day-old animals are an order of magnitude more methylated than the top 15 most methylated TEs (Fig. 7c). Contrary to this finding, *N*^*6*^-adenine methylation of the same set of TEs was almost one order of magnitude higher than the relative *N*^*6*^-adenine methylation level of the selected genes in 5-day-old animals (Fig. 7d). It is intriguing that the top 15 most *N*^*6*^*-*adenine methylated genes at day 1 became highly demethylated when adults reached the older stage. Relative N6-adenine methylation levels of the top 15 most *N*^*6*^-adenine methylated TE families at day 1 however further increased during this period.

Next, we aimed to compare the PCR-based results with those provided by the SMRTseq assay. To do this, we examined relative 6 mA levels at each *Tc3* stretch in animals with various ages, and compared to that of *cep-1* gene (Fig. 7e, f). We found that in 1-day-old animals, *cep-1* has twice as much modified adenines than the *Tc3* regions do (Fig. 7e), but in 5-day-old animals the relative *N*^*6*^-adenine methylation of the *Tc3* regions was nearly four times higher than the relative *N*^*6*^-adenine methylation level of *cep-1* locus (Fig. 7f). Based on both PCR- and SMRTseq-based 6 mA data (Figs. 6 and 7), we conclude that TEs are likely to exhibit a differential *N*^*6*^-adenine methylation during ageing.

Here, it is worth mentioning that SMRTseq technology has been criticized recently as it is often unable to specifically detect 6 mA sites from sample DNA due to a significant detection noise (nearby cytosine methylation), and the presence of bacterial DNA contamination and *N*^*6*^-adenine methylated RNA^42,43^. Despite these limitations, potential artefacts provided by the technology should affect both samples (1 and 5 days old adult nematodes) with a similar extent, so total 6 mA quantities at distinct TE families are comparable between young and older adults (our primary goal was not to accurately identify individual 6 mA sites within a single genome, rather we compared relative 6 mA levels in specific genomic loci between two samples, using the same detection approach).

### Downregulation of transposable elements increases thermotolerance in *C. elegans*

**Fig. 8:**
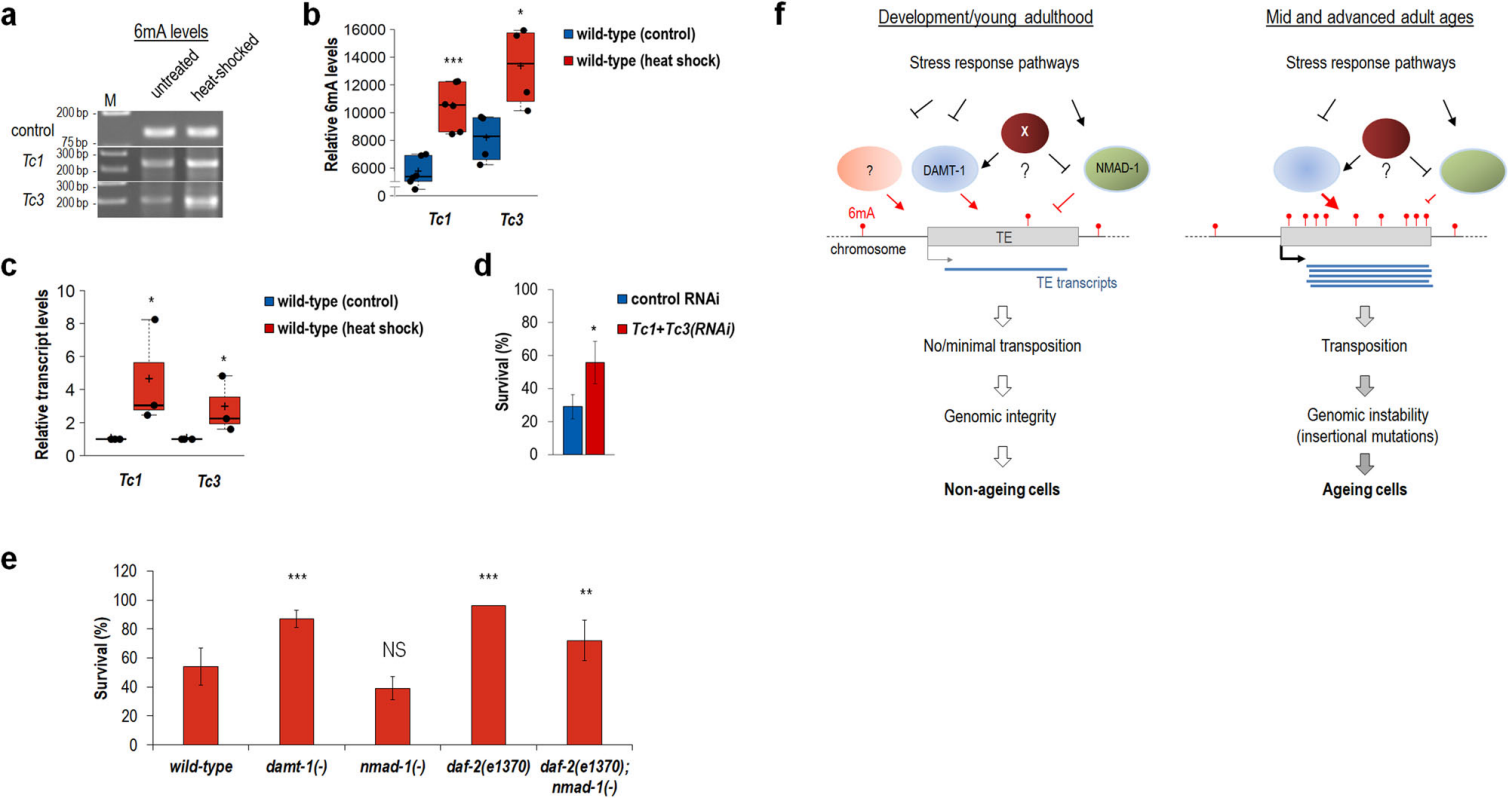
Downregulation of transposable elements promotes tolerance in nematodes to heat stress. **a** Heat stress increases 6mA levels at *Tc1* and *Tc3* sequences. Control animals were maintained at 20 °C. M indicates molecule weight marker (in base pair). **b** Quantification of relative 6mA levels. **c** Expression of *Tc1* and *Tc3* increases in response to heat stress (at 35 °C for 5 h). Transcript levels were determined by RT-qPCR. **d** Downregulation of *Tc1* and *Tc3* considerably increases the survival of nematodes exposed to heat stress. **e** Heat shock tolerance in mutant animals with altered 6 mA accumulation rates in TE loci. *damt-1(−)* mutants are more resistant, while *nmad-1(−)* mutants tend to be more sensitive, to heat stress than wild-type. *nmad-1(−)* mutation markedly suppresses the elevated tolerance of *daf-2(−)* mutants to heat stress. Bars indicate ±S.E.M., ***P < 0.001; independent two-sided *t* test with Bonferroni correction (**b–e**). For statistics, see Supplementary Tables 3, 4 and 6. Dot plot figures (**b**, **c**): centre lines show the medians; box limits indicate the 25th and 75th percentiles as determined by R software; whiskers extend 1.5 times the interquartile range from the 25th and 75th percentiles, outliers are represented by dots; crosses represent sample means; data points are plotted as circles. **f** Model showing that 6 mA levels gradually increase with age at mobile TE stretches to limit lifespan. This change is coupled with elevated TE transcription that accelerates the rate at which somatic cells age. Under normal conditions, 6 mA levels in active TE loci (grey box) steadily increase throughout adulthood. This change can potentially be established at different levels. First, the activity of NMAD-1 demethylase (green circle) may gradually attenuate during the adult lifespan (highlighted by yellow colouring) as a consequence of a co-factor (denoted by X, brown circle), the amount of which drops as the organism ages. Alternatively, X (brown circle) represents a repressor for NMAD-1 and its activity progressively increases during ageing. Second, such a co-factor (X), the amount of which gradually changes throughout adulthood, may elevate DAMT-1 (blue circle) activity during ageing. Increasing amounts of 6mA marks in TE loci lead to enhanced TE transcription and transposition. Under conditions of cellular stress, stress response pathways may modulate the expression of genes encoding enzymes involved in 6mA metabolism. Elevated TE activity causes genomic instability (insertional mutations), which may significantly contribute to the ageing process. TE-generated insertional mutations in maintenance systems, such as autophagy and DNA repair pathways (indirect way), or in other protein-encoding genes (direct way) can lead to the progressive accumulation of cellular damage. Question mark (?) within an orange circle denotes a hypothetical DNA *N*^*6*^-adenine methyltransferase functioning during development.

As shown above, *N*^*6*^-adenine methylation on TE sequences occurs at a halved rate in *daf-2(−)* mutants having a double lifespan relative to wild type (Fig. 6a–g). We therefore asked whether environmental stress factors can modulate 6mA accumulation at TE sites to explain the altered survival of nematodes deficient in a certain stress response pathway. To address this issue, we tested the tolerance of animals to high temperatures when TEs were inhibited (animals maintained at 20 °C were transferred at 35 °C for 5 h, then shifted back to 20 °C, and assayed for those being alive on the next day^49^). Relative 6mA levels at *Tc1* and *Tc3* sequences were first compared in untreated control versus heat-stressed nematodes. Results obtained show that the *N*^*6*^-adenine methylation process at these specific sites is activated in response to high temperatures (Fig. 8a, b, and Supplementary Table 6). This epigenetic change was coupled with elevated *Tc1* and *Tc3* expression (Fig. 8c, and Supplementary Table 6). *Tc1* and *Tc3* elements were then downregulated simultaneously, and this intervention considerably increased the survival of treated animals relative to untreated control (Fig. 8d). To strengthen these results, we also measured heat shock tolerance in *damt-1(−)* and *nmad-1(−)* mutant animals (Fig. 8e, and Supplementary Table 6). We found that the former survive better, while the latter tends to be more sensitive to heat stress, than the wild type. Furthermore, *nmad-1(−)* mutation significantly suppressed the increased tolerance of long-lived mutants defective for DAF-2 function (Fig. 8e, and Supplementary Table 6). Thus, nematodes with a lowered rate at which 6mA accumulates in TE loci live longer and more resistant to heat stress than normal. In contrast, a genotype conferring a faster 6mA accumulation dynamics at TE sequences limits lifespan and renders the animal more sensitive to heat stress.

Heat stress data above indicate that TEs mediate cellular stress through acting downstream of the heat stress response pathway in this organism. Indeed, neither *Tc1* + *Tc3* RNAi co-treatment nor inactivating mutations in *damt-1* and *nmad-1* could influence the expression of *hsp-16.2* (heat-shock protein), which is a target gene of the nematode heat stress response pathway (Supplementary Fig. 13a, b and Supplementary Table 7)^50^. We also tested the expression of *sod-3* (superoxide dismutase) and *gcs-1* (gamma glutamyl cysteine synthetase), targets of the *C. elegans* nutrient-sensing and oxidative stress response pathways, respectively^50,51^, in control vs. *Tc1* + *Tc3* co-downregulated genetic backgrounds, but detected no obvious difference between the treated and corresponding control samples (Supplementary Fig. 13c–f and Supplementary Table 7). Similarly, downregulation of *nmad-1* and *damt-1* did not alter *hsp-16* and *gsc-1* expression (Supplementary Fig. 13g, h and Supplementary Table 7). This raises the possibility that TEs and factors that influence 6mA levels at TE sequences function downstream of stress response pathways in lifespan determination. Alternatively, TEs and factors influencing 6mA levels may act parallel to these pathways.

In the light of the above results, we next analysed available RNAseq data in wild-type versus *daf-2(−)* mutant animals^52^, now focusing on *damt-1* and *nmad-1* expression. The DAF-2/IGF-1-mediated pathway responses to nutritional stress via inhibiting the FOXO-like transcription factor DAF-16. In *daf-2(−)* mutant backgrounds, DAF-16 is (hyper)active and regulates target genes required for enhanced stress tolerance and longevity. The analysis revealed that DAF-16 represses *damt-1* but activates *nmad-1* expression (*damt-1* transcript levels were lower, while *nmad-1* transcript levels were higher, in mutant animals defective for DAF-2 function as compared with normal; Supplementary Fig. 14a and Supplementary Table 8). Although these gene expression changes were rather modest, but still below the significance limit (*P* < 0.05). In good accordance with these results, another set of available RNAseq data from wild-type, *daf-2(−)* single mutant and *daf-2(−);daf-16(−)* double mutant animals^53^ showed that the effects of DAF-2 on *damt-1* and *nmad-1* expression tend to be dependent of DAF-16 activity (Supplementary Fig. 14b and Supplementary Table 8). These findings also denote that 6 mA levels increase with a lower rate at TE sequences in *daf-2(−)* mutants compared to wild-type.

Why does the *N*^*6*^-adenine methylation pattern at TE stretches change with age under normal, stress-free conditions? A reasonable explanation would be a life-long, progressive increase in *damt-1* expression (growing *N*^*6*^-adenine methyltransferase activity) or a gradual decrease in *nmad-1* expression (failing *N*^*6*^-methyladenine demethylase activity). Both alternatives would lead to the same outcome, increasingly growing 6mA levels in TE regions. Thus, we determined the expression activity of *damt-1* and *nmad-1* genes throughout adulthood, but found no significant changes that could underlie the gradually increasing mobilization of TEs over time (Supplementary Fig. 15). The expression of *damt-1* remained nearly constant during ageing (Supplementary Fig. 15a), whereas *nmad-1* exhibited even an increased tendency to produce transcripts (Supplementary Fig. 15b). The latter results were supported at the level of NMAD-1 protein accumulation (Supplementary Fig. 15c, d). An earlier study of transcriptional profile of *C. elegans* ageing has provided similar results regarding these genes^52^. We conclude that age-associated changes in 6 mA levels at TE sequences are driven by mechanisms other than altered expression of genes encoding enzymes involved in DNA 6 mA modification.

## Discussion

Using reverse transcriptase inhibitors blocking retrotransposon mobilization, researchers could extend lifespan in *Drosophila*^22^ and delay cellular senescence in mice^25^. However, these compounds may have multiple targets, thereby affecting the function of other proteins in addition to reverse transcriptases. Our present study provides the first effective RNA interference-mediated downregulation of active TE families in a genetic model system, *C. elegans*. We showed that specific silencing of *Tc1* and *Tc3*, the most active TEs in this organism^26^, slows down the ageing process at different temperatures (Fig. 1a, c, e, g, and Supplementary Fig. 2a–d). In addition, co-repressing these mobile DNA elements additively promoted longevity (Fig. 2a–c). Downregulation of *Tc14*, which is another active TE in this organism^26^, also conferred a statistically significant lifespan advantage over control (Fig. 1i, and Supplementary Fig. 2g). In good accordance with these results, somatic expression of Piwi proteins, which normally inhibit TE activity in the non-ageing germline, lengthened lifespan in transgenic animals (Fig. 3a, d, g, and Supplementary Fig. 4). Taken together, we conclude that the rate at which somatic cells age is influenced by TE activity, and that DNA transposons contribute to lifespan determination.

*C. elegans* appears to be a unique model for exploring the contribution of TEs to ageing. First, only 12% of its genome derives from TE-related repetitive sequences, the majority of which are inactive elements^26^. An active TE family in this species contains only 5-31 copies, the inhibition of which could promote longevity (Figs. 1a, c, e, g, i and 2a–c, and Supplementary Figs. 2a–d, g). Second, it was suggested earlier that TEs destabilize the genome of hyper-proliferating somatic cells, thereby protecting tissues from undergoing tumorigenesis^15^. Somatic expression of Piwi proteins inhibiting TE activity indeed effectively induces tumour development in mice^54^, and tumorous cell lines tested so far have been proven all positive for Piwi activity^12^. Thus, repression of TEs in somatic cells may lead to cancer (a pleiotropic effect), causing an early death rather than extending lifespan. However, tumours are yet to be induced in the *C. elegans* soma, a special feature of the organism that enables TE inhibition without causing cancer.

Although longevity advantage conferred by downregulation of single TE families was quite modest, this can be explained by the relative ineffectiveness of RNAi treatments (Fig. 1b, d, f, h, j), the high copy numbers of genes targeted for downregulation (10-31 copies per a TE family; Supplementary Fig. 1), the rather late onset of depletion during lifespan (see the Methods section), and the remaining mutagenic effect of other active TE families that were not targeted in a given trial.

Results obtained here demonstrate that active TE stretches become increasingly *N*^*6*^-methylated on adenine nucleobases during the adult lifespan (Figs. 4, 6 and 7). This epigenetic modification progressively elevates TE transcription, which, in the absence of Piwi-piRNA complexes, may cause considerable levels of mutagenic events (genomic instability) in somatic cells at advanced ages (Figs. 2e–h and 6j). DNA *N*^*6*^-adenine methylation has been recently discovered in distantly related animal species^36,37,39^, but its functional significance remains largely unclear. This type of DNA modification was suggested to have a role in facilitating TE transposition in *Drosophila*^37^ while inhibit TE activity in mammals^39^. Several recent studies however debate the presence of 6mA epigenetic mark in animal genomes, and attribute it to methodological errors^40–43^. Here, we developed a reliable (sequence-specific and free of artefacts caused by bacterial DNA or RNA contamination) PCR-based method to accurately determine relative 6 mA levels at selected genomic sites (Fig. 4b, and Supplementary Fig. 7). Using this technique, we showed that in *C. elegans* 6mA levels in TE loci gradually increase with age (Fig. 6a, b). Thus, *N*^*6*^-adenine methylation at specific genomic sites influences the rate at which somatic cells age. Since 6mA deposition on active TE stretches is proportional with the age of the organism (Fig. 6a, b), it is possible that this epigenetic modification will be a target to accurately determine age from DNA. We propose that 6mA levels at active TE stretches serve as a signature of ageing.

We also demonstrated that the excision dynamics of a *Tc3* stretch correlates with the actual rate of *N*^*6*^-adenine methylation at the given genomic site (Fig. 6j). Thus, there is a causative relationship among these processes; *N*^*6*^-adenine methylation increasingly occurs at active TE sequences (Fig. 6a–g), and progressively elevates their transcriptional activity (Fig. 5a–d) and mobilization (Figs. 2e, f and 6j). This causes genomic instability (insertional mutations in functional DNA sequences) (Figs. 2g, h and 6j), leading to an accelerated ageing rate and, as a consequence, shortened lifespan (Figs. 1–3 and 5). However, the potential effects of DAMT-1 and NMAD-1 on other genes in lifespan determination cannot be excluded.

*daf-2(−)* mutant animals live two times longer than normal^45^. In this mutant genetic background, 6mA marks accumulated with age in TE loci at a halved rate relative to wild-type (Fig. 6a–g). So, longevity correlates to a lower rate at which 6mA accumulates in TE loci. In *daf-2(−)* mutants, the expression of *damt-1* gene was decreased, while the expression of *nmad-1* gene was increased, as compared with the wild type (Supplementary Fig. 14). Thus, the transcription factor DAF-16, which operates as the effector of the DAF-2/IGF-1-mediated, nutrient-sensing stress response pathway, may modulate the activity of genes determining 6mA levels. Similar to DAF-16, other transcription factors mediating the effect of stress response pathways, such as SKN-1 and HSF-1 (these proteins participate in oxidative and heat stress responses, respectively), may also be involved in controlling 6mA levels at specific genomic sites. In a good agreement with this suppose, *Tc1* and *Tc3* became hypermethylated on adenine nucleobases and over activated in response to heat stress (Fig. 8a, b). The fact that TE mobilization elevates at high temperatures is well documented in other organisms as well^55,56^. Moreover, oxidative, radiation and nutritional stress factors were also reported to promote TE transcription^22,57,58^. Based on these data, we suggest that TEs act downstream of stress response pathways in lifespan determination (Fig. 8f).

Under normal conditions, however, other factors should modulate the rate at which 6mA mark accumulates with age in active TE loci. To examine whether this mechanism is established at the transcriptional control of *damt-1* encoding a putative DNA *N*^*6*^-adenine methyltransferase and/or *nmad-1* coding for a DNA *N*^*6*^-methyladenine demethylase, we assessed transcript levels of these genes at different stages of adulthood but found no significant alterations that could explain why *N*^*6*^-adenine methylation patterns of TEs increase during ageing. *damt-1* transcript levels were nearly constant, whereas *nmad-1* expression and NMAD-1 protein accumulation even increased during the adult lifespan (Supplementary Fig. 15). Presumably, the latter changes are merely a consequence of a genetic compensatory mechanism^59,60^ to overcome a gradual loss in NMAD-1 activity. The age-related accumulation of 6 mA epigenetic mark in active TE sequences is thus not a consequence of changes in transcript/protein levels of factors mediating DNA *N*^*6*^-adenine methylation and *N*^*6*^-methyladenine demethylation. A gradual increase in 6 mA levels at specific genomic sites during adulthood may be established at different levels. These data prompted us to propose a model in which a factor unidentified to date (indicated by X in Fig. 8f) would gradually increase the activity of DAMT-1 *N*^*6*^-adenine methyltransferase or gradually lower the activity of NMAD-1 6 mA demethylase during adulthood (Fig. 8f). These two options would lead to the same result: a progressive, lifelong accumulation of 6 mA mark in active TE loci. Such a factor could be a regulatory protein or an environmental component. Indeed, oxygen was reported to act as a co-factor of NMAD-1/AlkB family of demethylases^61^, and hypoxic milieu is a general feature of ageing cells^62,63^. Thus, a gradual reduction in intracellular oxygen levels throughout adulthood may explain why NMAD-1 activity decreases during ageing.

The important question of why TEs are differentially *N*^*6*^-adenine methylated however remains unresolved. In numerous animal species, de novo DNA methylation that occurs during early development primarily generates 5-methylcytosine (5mC) marks, most of which resides in TE loci^64,65^. TEs are considered intragenomic parasites, and 5-cytosine methylation at TE sequences may represent a defence mechanism that has evolved against the invasion of mobile DNA elements to preserve genomic integrity. The mechanisms underlying 5-cytosine methylation involve a Piwi/piRNA-directed machinery, and piRNA are transcribed from TE-related sequences located in piRNA gene clusters^66^. DNA *N*^*6*^-adenine methylation may also be mediated by a piRNA-driven mechanism, explaining why 6 mA preferably accumulates at TE sequences during ageing. Further studies will certainly clarify this important issue.

The regulation of ageing has been intensely studied and relatively well understood during the last decades^67,68^. However, despite its medical, social and economic significance, the mechanisms underlying the ageing process, that is, genetic factors that actually cause ageing, remain largely unknown. This problem may stem from the fact that the mechanisms involve multicopy factors, the parallel inhibition of which has not yet been established. TEs constitute a large fraction of eukaryotic genomes, and their activity, as shown here, contributes to ageing. To test whether TEs represent an underlying mechanism of the ageing process, one should simultaneously block the mobilization of essentially all active TE families (both retroelements and DNA transposons) in an organism, as they are effectively repressed in the non-ageing germline by the Piwi-piRNA pathway, and detect extreme longevity.

## Methods

### Strains and genetics

Nematodes were maintained on standard Nematode Growth Medium (NGM) as described previously^69,70^, and fed with *Escherichia coli OP50* bacteria. The following *C. elegans* strains were used in this study.

Bristol (N2) as wild-type

DR1344 Bergerac BO

CB1370 *daf-2(e1370)III*

TJ1060 spe*-9(hc88)I; rrf-3(b26)II*

VC2552 *nmad-1(ok3133)III*

VC40319 *C18A3.1(gk961032)II*

TTV680 *unc-119(ed3)III; eluEx390[p*_*hsp-16.2*_*::Tc3 + unc-119(+)]*

TTV681 unc-119(ed3)III; eluEx391[p_hsp-16.2_::prg-1 + unc-119(+)]

TTV614 *unc-119(ed3)III; eluEx330[p*_*hsp-16.2*_*::prg-1::gfp + unc-119(+)]*

TTV491 *unc-119(ed3)III; eluEx364[p*_*hsp-16.2*_*::prg-2 + unc-119(+)]*

TTV615 *unc-119(ed3)III; eluEx358[p*_*hsp-16.2*_*::ppw-2 + unc-119(+)]*

TTV721 *unc-119(ed3)III; eluIs314[p*_*nmad-1*_*::NMAD-1::GFP + unc-119(+)]*

CF1553 *muIs84[(pAD76) sod-3p::GFP + rol-6(su1006)]*

TJ375*gpIs1[hsp-16.2::GFP]*

TTV804 *nmad-1(ok3133)III; gpIs1*

TTV805 *nmad-1(ok3133)III; muIs84*

TTV806 *C18A3.1(gk961032)II; gpIs1*

TTV807 *C18A3.1(gk961032)II; muIs84*

Prior to performing lifespan assays, VC2552 and VC40319 strains were isogenized by outcrossing 3 times with the wild type.

### Lifespan assays

Lifespan assays were carried out at 15 °C, 20 °C and 25 °C, as described previously^69–71^. For synchronization, 20-30 gravid, well-fed adults were transferred to a new agar plate containing NGM seeded with *E. coli* [OP50 or HT115(DE3)] bacteria to lay embryos for 4–5 h, and then removed. Alternatively, embryos were prepared by NaOH/hypochlorite treatment. Approximately 60-70 F1 young adults were transferred to new NGM plates supplemented with 300–400 mg/ml FUdR (5-fluoro-2′-deoxyuridine, Sigma #50-91-9) (*t* = 0). Sterile F1 adults were then assayed. Animals that climbed up the wall of plastic dishes or exhibited a protruded/burst vulval phenotype and thereby died prematurely were excluded from the analysis. Animals were considered dead when they stopped pharyngeal pumping and responding to touching. For measuring the lifespan of TE- (transposable element) specific double-stranded (ds) RNA-treated animals, wild-type hermaphrodites at the young, gravid adulthood were transferred onto RNAi (RNA interference) plates. This relatively late onset of treatment during lifespan allowed TEs to express potential developmental roles (during evolution certain TEs became domesticated and acquired developmental roles)^27^. Indeed, *Tc1* depletion starting from the L1 larval stage caused early lethality instead of promoting longevity. SPSS 17 software was used to calculate mean lifespan and perform statistical analysis. *P* values for comparing Kaplan-Meier survival curves between two groups were determined using log-rank (Mantel–Cox) tests, and P values for comparing mean lifespans were determined using Independent two-sample Student’s two-sided *t* tests with Bonferroni correction. *E. coli* HT115(DE3) RNAi feeding bacteria were grown to OD_600_ = 0.5 in LB medium containing 50 µg/ml ampicillin (Sigma, #69-53-4) in final concentration. L4/young stage adults were transferred to plates containing 300–400 mg/ml FUdR, 50 µg/ml ampicillin and 0.4 mM IPTG (isopropyl β-D-1-thiogalactopyranoside, Sigma #367-93-1) in final concentration. Empty vector- (L4440 and T444T) containing bacteria were used as control.

### Generation of DNA constructs and transgenic strains

Overexpressing single components of the Piwi-piRNA pathway in the soma is expected to induce the activity of the pathway because its certain components were found to accumulate somatically^12,34^ and Piwi proteins, by themselves, can mediate transcript degradation^34^. pPD118.28 served as the cloning vector for generating a translational fusion (functional) *hs*::PRG-1::GFP construct. *pPD49.78* served as the cloning vector for somatic overexpression of *prg-1* (*hs*::PRG-1), *prg-2* (*hs*::PRG-2), *ppw-2* (*hs*::PPW-2) and *Tc3 (hs*::*Tc3)* full length coding regions. These vectors contain an inducible *hsp-16.2* heat shock promoter that is active in the majority of somatic cells. Note that the promoter is leaky, enabling gene expression even at normal culturing temperatures between 15-25 °C. These constructs were cloned by using the following restriction enzymes (in parenthesis), as well as forward and reverse primers. *hs*::PRG-1::GFP: (BamHI + AgeI) 5′-CGC GGA TCC GCG ATG GCA TCT GGA AGT GGT C-3′ and 5′-TAT ACC GGT GTC AAG AAG AAC AGC TTG TCA CG-3′; *hs*::PRG-1: (Gibson) 5′-CAA ACT ATA ATC ATC TCA CTG GAT CAA TGG CAT CTG GAA GTG GTC G-3′ and 5′-AAT ACC ATG GTA CCG TCG ACG CTA GTT ACA AGA AGA ACA GCT TGT CAC GAA GAC A-3′; *hs*::PRG-2: (BamHI + NheI) 5′-CGC GGA TCC GCG CCG ATT GAG ATG ATT CAG AAC GAC-3′ and 5′-CTA GCT AGC TAG TTA CAA GAA GAA CAG CTT GTC ACG-3′; *hs*::PPW-2: (XmaI + KpnI) 5′-CCC CCC GGG GGG ATG CCT GCT ACA CCG GTT-3′ and 5′-CGG GGT ACC CCG TTA AGC ATT GAC ACG GCG G-3′; *hs::Tc3*: (NheI + NcoI) 5′-AAA AAA GCT AGC ATG CCT CGA GGA TCT GCC C-3′ and 5′-AAA AAA CCA TGG ATA GTT AAT CGG GTT TCC TTG TGT GCG-3′.

To generate a *p*_*nmad-1*_::NMAD-1::GFP translational fusion reporter construct, a 2.37 kb-long genomic fragment containing 5′ regulatory and coding regions of *nmad-1* was amplified by the following forward and reverse primers: *5*′*-*AAA ACT GCA GGG CCC TTT CCT AGT TTT TGC*−3*′ *and 5*′*-*AAA CCC GGG ATT TCC CCA AAT CCA CAT ATC A-3′. The resulting fragment was cloned into pPD95.75 vector, using PstI and SmaI restriction enzymes. To generate a *p*_*damt-1*_::DAMT-1::GFP translational fusion construct, a 3.7 kb-long genomic fragment containing of 5′ regulatory and coding regions of damt-1 was amplified by using the following forward and reverse primers: *5*′*-*AAA CTG CAG AGT CCA ACC AAA TCA AAA TCG*−*3′ and 5′*-*AAA GGA TCC CGA GTG AAA ACA TGT TCA GAT TG*−3*′. The resulting fragment was cloned into pPD95.75 vector at the PstI and BamHI restriction sites.

Transgenic animals were generated by microparticle-mediated bombardment (biolistic transformation) into the *unc-119(−)* (uncoordinated) mutant genetic background, using the co-transformation marker *unc-119(+)* gene^72^. Transgenic lines were established by picking up F1 animals with normal movement. Using a Biolistic PDS-1000/He particle delivery system (BioRad), 10-15 μg linearized plasmid DNA was bombarded onto *unc-119(ed3)* mutant hermaphrodites at the L4/adult stages. Fluorescent images were taken by Zeiss AxioImager Z1 epifluorescence microscope.

### RNAi constructs

L4440 served as the original RNAi vector (equivalent to pPD129.36; Fire Lab 1997 Vector Kit). The 344 bp-long MCS (Multi Cloning Site) fragment from L4440 was amplified by using left and right primers that contain T7 terminator sequences at both ends, together with 5′ PciI- and 3′ NgoMIV-specific restriction sites. PCR fragment was ligated into L4440 to create a modified RNAi vector, T444T^27^. This vector can generate specific dsRNAs only, without amplifying vector backbone sequences, thereby producing more effective phenotypes than the original L4440. *Tc1*, *Tc3*, *Tc14*, *Cele14*, *nmad-1* and *damt-1* genomic fragments were cloned into T444T by using the following restriction enzymes (in parenthesis), and forward and reverse primers. T444T: (PciI+ NgoMIV) 5′-TTT TTT ACA TGT CAA AAA ACC CCT CAA GAC CCG TTT AGA GGC CCC AAG GGG TTA TGC TAG TAA TAC GAC TCA CTA TAG GGA GAC CGG CAG-3′ and 5′-TTT TTT GCC GGC CAA AAA ACC CCT CAA GAC CCG TTT AGA GGC CCC AAG GGG TTA TGC TAG TAA TAC GAC TCA CTA TAG GGC GAA TTG GGT ACC G-3′; *Tc1*: (BglII + Acc65I) 5′-CTA GCT AGC TAG AAC AGC TTG ACA ACG ACG TG-3′ and 5′-CTA GCT AGC TAG AAC ATC CTC CGA TCA GCA AG-3′; *Tc3*: (BglII + Acc65I) 5′-TTT TTT AGA TCT ATG CCT CGA GGA TCT GCC CT-3′ and 5′-TTT TTT GGT ACC GGG TAA GTC TTG TTC TGA GCA TAC ACG-3′; *Tc14*: (BglII + Acc65I) 5′-TTT TTT AGA TCT AGA TCC GGA CGC GGA AGA CC-3′ and 5′-TTT TTG GTA CCC CAG CTC GTC CCA TGC CTT C-3′; *Cele14*: (Gibson) 5′-TAA TAC GAC TCA CTA TAG GGC GAA TTG GGT ACA CTG ATA AGG TAT TAC ACG TGG AGT C-3′ and 5′-TAA TAC GAC TCA CTA TAG GGA GAC CGG CAG ATC GAA TTC AAT AAT ACC ACG TGG TGT CAG G-3′; *nmad-1*: (Acc65I + BglII) 5′-TTT TTT AGA TCT TGT GAG ACA ACG GAA CGT GT-3′ and 5′-TTT TTT GGT ACC TCC TGA TGC ATC TCA ATG GCA-3′; *damt-1*: (BglII + Acc65II) 5′-TTT TTT AGA TCT CGT TGA ACG TGT CAA TCC CC-3′ and 5′-TTT TTT GGT ACC CAT TGG AAC GCT GGC AAA AAC-3′.

### Transcript quantification by semi-qPCR

Total RNA was extracted from 100 synchronized animals, using TRI reagent (Sigma, #T9424), and used for first strand cDNA synthesis by RevertAid First Strand cDNA Synthesis Kit (Thermo Scientific, #K1622). Mean relative mRNA levels were determined by using *cdc-42* as a reference gene. Forward and reverse primers and PCR conditions were as follows. *cdc-42*: 5′-CTT CGA CAA TTA CGC CGT CAC-3′ and 5′-CGA AAT TTC AGG CAC CCA TTT TTC-3′. Initial denaturation at 95 °C for 30 s, then 29 cycles of denaturation at 95 °C for 10 s, annealing at 57 °C for 30 s, extension at 68 °C for 30 s, and final extension at 68 °C for 2 min. Samples were tested at 23, 26 and 29 cycles. *Tc1*: 5′-TGC AAA TTC AAC GTT CTC CG-3′ and 5′-TTC TCA AAG CGA CGG ATT TC-3′. Initial denaturation at 95 °C for 30 s, then 35 cycles of denaturation at 95 °C for 10 s, annealing at 55 °C for 30 s, extension at 72 °C for 30 s, and final extension at 72 °C for 2 min. Samples were tested at cycles 29, 32, and 35. *Tc3*: 5′-ATT CTC GAC GCT TGG AAG TC-3′ and 5′-GTT TCC TTG TGT GCG GAT G-3′; initial denaturation at 95 °C for 30 s, then 32 cycles of denaturation at 95 °C for 10 s, annealing at 55 °C for 30 s, extension at 72 °C for 30 s, and final extension at 72 °C for 2 min. Samples were tested at 29, 32 and 35 cycles. *Tc14*: 5′-GTG AAC CCA TCG TTC GTT TTC-3′ and 5′-CGT GCA ATG GTG GTA GAA AC-3′; initial denaturation at 95 °C for 30 s, then 32 cycles of denaturation at 95 °C for 10 s, annealing at 55 °C for 30 s, extension at 72 °C for 30 s, and final extension at 72 °C for 2 min. Samples were tested at 29, 32 and 35 cycles. *prg-1* transgene: 5′-TCT TCA CGA TGA TGC CAA TG-3′ and 5′-TGG GAC AAC TCC AGT GAA AAG-3′; initial denaturation at 95 °C for 30 s, then 29–35 cycles of denaturation at 95 °C for 10 s, annealing at 55 °C for 30 s, extension at 68 °C for 30 s, and final extension at 68 °C for 2 min. *ppw-2* transgene: 5′-TTT GAA GAA AAT GAC CGA TGG-3′ and 5′-AGA TGG CGA TCT GAT GAC AG-3′; initial denaturation at 95 °C for 30 s, then 29–35 cycles of denaturation at 95 °C for 10 s, annealing at 57 °C for 30 s, extension at 68 °C for 30 s, and final extension at 68 °C for 2 min. Where products were separated by agarose gel electrophoresis we used Thermo Scientific™ GeneRuler 1 kb Plus DNA Ladder (#SM1331).

### RNA extraction and quantification of transcript levels by real-time qPCR

Total RNA was extracted from 50 synchronized animals per each sample, using TRI reagent (Sigma, T9424), and RNA isolation was performed according to the Direct-zol^TM^ RNA MiniPrep kit protocol (Zymo Research, R2050). Total RNA samples were used for first strand cDNA synthesis by RevertAid First Strand cDNA Synthesis Kit (Thermo Scientific, K1622). Real-time quantitative-PCR was performed on LightCycler 96 System (Roche, FastStart Essential DNA Green Master, 06402712001) under the following conditions: denaturation at 95 °C for 10 min, followed by 45 cycles of amplification (10 s, 95 °C; 10 s, 58 °C and 20 s, 72 °C). In this method, the fluorescence of double stranded DNA-binding SYBR-Green dye indicates the PCR products. Melting curve analysis was performed to confirm the correct PCR product size and the absence of nonspecific bands. Mean relative mRNA levels were determined by normalizing the PCR threshold cycle number of *tc1*, *tc3*, *tc14*, *nmad-1*, *damt-1* and *prg-1* with that of *cdc-42* reference gene. For qPCR amplification, the following primers were used. *cdc-42*: 5′-CTT CGA CAA TTA CGC CGT CAC-3′ and 5′-CGA AAT TTC AGG CAC CCA TTT TTC-3′; *tc1*: 5′-AAA AAA GCT AGC ATG GAT CGC AAC ATC CTC CGA T-3′ and 5′-CC CAC GCA ACT CGA GCC-3′; *tc3*: 5′-GGT TGT CTT CTC C-3′ and 5′-GTT TCC TTG TGT GCG GAT G-3′; *tc14*: 5′-GTT TCT ACC ACC ATT GCA CG-3′ and 5′-TAC CAT TCG GCA CAG AGA AC-3′; *nmad-1*: forward 5′-AAC GGA ACG TGT GAA AAA GC-3′ and reverse 5′-CGA ATG TGA TCG TAA ATG AAG ACT-3′; *damt-1*: forward 5′-CCT CTT CGA CTT GAT CAT TGC- 3′ and reverse 5′-GAA CTT CTT CGT CCA TTT GAT ATG T-3′; *prg-1*: forward 5′-GTG AAC ATT CCG CTT AAA AAC AC-3′ and reverse 5′-CTT CAA GGC TTT TCG AAC AAA G-3′. Statistical analysis was performed by RStudio (version 0.99.903) software. Since data did not show a normal distribution, Mann-Whitney U-test was used to determine significance levels.

### Determining *N*^*6*^-methyl adenine (6mA) levels at TE stretches

Adenine *N*^*6*^-methylation is generally detected by SMRT (single molecule real-time) sequencing^48^, which is still a rather expensive and laborious technique. Here we developed a relatively simple, PCR-based method to detect the relative *N*^*6*^-methylation level of any adenine base at any repetitive sequence. Genomic DNA from adults with age of days 1 and 11 was digested with DpnI or PvuII for 20 min at 37 °C, then a linker DNA (5′-TAG ATC TGA CCT AAC GGT AAG AGA GTT TCA TAA TAT TTT TTT TTT TTT TTT AT―TAT GAA ACT CTC TTA CCG TTA GGT CAG ATC TA-3′) was ligated to the digested genomic DNA for 24 h at 4 °C. DpnI cleaves DNA only when its restriction site (GATC) is methylated at position A, PvuII cuts any DNA sequence containing its restriction site (CAGCTG). Mean relative DNA levels were determined by using *Tc3* PvuII primers as reference. Forward and reverse primers, and PCR conditions were as follows. *Tc1* (DpnI): 5′-TCT TAC CGT TAG GTC AGA TCT ATC C-3′ and 5′-CAT GGG GAT AGC TTT CCA AG-3′, initial denaturation 95 °C, 30 s, then 40 cycles of denaturation at 95 °C for 10 s, annealing and extension at 50 °C for 30 sec; *Tc3* (DpnI): 5′-ATG AAA CTC TCT TAC CGT TAG GTC AGA TCT ATC TC-3′ and 5′-TGA CCA AAC TTT TCA GCT GGT TGT CC-3′, initial denaturation 95 °C for 30 s, then 35 cycles of denaturation at 95 °C for 10 s, annealing and extension at 57 °C for 30 sec; *Cele14* (DpnI): 5′-ACT CTC TTA CCG TTA GGT CAG ATC TAT-3′ and 5′-CCA GAA AAA TTG TGA CGT CAG CAC GCT C-3′, initial denaturation at 95 °C for 30 s, then 35 cycles of denaturation at 95 °C for 10 s, annealing and extension at 57 °C for 30 s; *Tc3* (PvuII) (Reference): 5′-TGA AAC TCT CTT ACC GTT AGG TCA GAT CTA CTG-3′ and 5′-TCA ATA GTT AAT CGG GTT TCC TTG TGT GCG GAT GAT C-3′, initial denaturation at 95 °C for 30 s, then 32 cycles of denaturation at 95 °C for 10 s, annealing and extension at 57 °C for 30 s.

To determine relative 6mA levels at TE stretches and *cep-1* locus throughout adulthood, genomic DNA was isolated from staged population of wild-type and *daf-2(e1370)* mutant adult animals grown at 20 °C, using Thermo ScientificGeneJET GenomicDNA Purification Kit (#K0721). Genomic DNA was then digested with DpnI at 37 °C for 20 min, and ligated with a linker fragment at 4 °C for overnight. 10 pg DNA was used for PCR amplification to detect relative 6mA levels at a given adenine basis.

### Single-molecule real-time (SMRT) sequencing and bioinformatics

For SMRT sequencing, genomic DNA samples from adult animals staged at the 1- and 5-day were prepared as follows. Wild-type animals collected from 40 NGM plates were synchronized by bleaching (hypochlorite treatment). When the animals reached the young adult stage at 20 °C, they were transferred to 400 NGM plates containing 50 μg/ml FUdR (5-Fluoro-2′-deoxyuridine, Sigma-Aldrich, #F0503). Around 100 animals per plate were transferred. After 1 day, 200 plates were harvested for 1-day-old animals, while the remaining 200 plates were harvested for 5-day-old animals. Genomic DNA was then isolated from the samples with GeneJET Genomic DNA Purification Kit (Thermo Scientific™ GeneJET Genomic DNA Purification Kit, #K0721), and then subjected to SMART sequencing. We used command line tools of PACBIO SMRT Link (version 7.0.1) for methylation calling. First PacBio sequences (stored in unaligned BAM files) were mapped to the *C. elegans* reference genome (Caenorhabditis_elegans.WBcel235.dna.toplevel.fa) by pbalign (version 0.4.1). Then, ipdSummary (version 2.4) was used on the aligned BAM files to detect DNA base-modifications from kinetic signatures. The methylation levels of repeat sequences and cDNA sequences were calculated by in-house python scripts using the gff files, and the annotation files of Dfam release 3.1 (https://www.dfam.org/releases/Dfam_3.1/annotations/ce10/, 10.1093/nar/gkv1272). To determine the relative 6 mA content for a given DNA sequence, we divided the amount of 6 mA signals detected in the given section by the length of that segment to obtain the relative amount of 6 mA in that segment. For the TE-derived sequences, the length of the given TE sections was proportional to the size of the TE section multiplied by the copy number of the given TE. Thus, the analysis was not affected by the high copy numbers of TEs.

### Detecting mobilization of a single *Tc3* stretch

We developed a relatively simple, PCR-based method to detect the mobilization of a single *Tc3* stretch, *R10H1.3*. Genomic DNA from populations of adult animals at the 1-day and 14-day stages was purified by using GeneJET Genomic DNA Purification Kit (Thermo Scientific™ GeneJET Genomic DNA Purification Kit, #K0721), and adjusted to the same concentration, using a nanodrop method. Mean relative DNA levels were determined by using *Tc3* qPCR control primers as reference. Forward and reverse primers, and PCR conditions were as follows. For *Tc3* mobilization: 5′-AGA CCA AAA AGA CGG TGC GTA-3′ and 5′-AAC GGT AAT TGC CGG TCC AA-3′, initial denaturation at 95 °C for 30 s, then 32 or 37 cycles of denaturation at 95 °C for 10 s, annealing and extension at 63 °C for 30 s. For *Tc3* qPCR control: 5′-GTT TCC TTG TGT GCG GAT G-3′ and 5′-ATT CTC GAC GCT TGG AAG TC-3′, initial denaturation at 95 °C for 30 s, then 30 cycles of denaturation at 95°Cfor 10 s, annealing and extension at 66 °C for 30 s.

### Thermotolerance assay

The assay was performed as described earlier^49^. Briefly, worms were synchronized and maintained at 20 °C. Young (non-gravid) adult hermaphrodites were transferred to fresh agar plates seeded with *E. coli* OP50, then exposed to heat shock by transferring them at 35 °C for 5 h. Animals were then transferred back at 20 °C, and allowed to recover overnight. The survival rate was determined by counting the number of live versus dead individuals. At least 60 animals per strain were counted by light microscopy in 3 independent trials. Statistical significance was determined by independent samples *t* tests, using the SPSS 17 software. TE transcript levels of heat-shocked animals were determined by qPCR using the following primers: *cdc-42* (control): forward 5′-CTT CGA CAA TTA CGC CGT CAC-3′ and reverse 5′-CGA AAT TTC AGG CAC CCA TTT TTC-3′; *Tc1*: forward 5′- TGC AAA TTC AAC GTT CTC CG-3′ and reverse 5′-TTC TCA AAG CGA CGG ATT TC-3′; *Tc3*: forward 5′-CGA ACG AAT TGG AGT AAA GTT GTC-3′ and reverse 5′-GTT TCC TTG TGT GCG GAT G-3′.

### Fluorescent microscopy

Animals were synchronized, and approximately 60-70 L4 larvae were transferred to a new NGM plate supplemented with 300 mg/ml FUdR (5-fluoro-2′-deoxyuridine, Sigma). Adult animals were then assayed at days 1, 5 and 10 at 20 °C. In the case of assessing *hsp-16.2::gfp* expression levels, nematodes were heat shocked at 35 °C for 30 min and microscopy was performed after a recovery at 20 °C for 3 h. Transgenic worms were immobilized by adding 0.1 M sodium-aside in M9 buffer. Images for quantitative analysis were taken by a Zeiss AxioImager Zeiss M2 epifluorescence microscope (objective EC Plan-NeoFluar 10 × 0.3 NA). Expression data were obtained from whole animals. Statistical significance was determined by independent samples *t* tests (SPSS 17 software). The software ImageJ was used for quantitative analysis.

### Statistics and reproducibility

Each statistical test implemented in this study is indicated either within the figure legends or the supplementary materials tables. Experiments which display results from representative experiments, including gel images and microscopic images, were conducted with a minimum of three biological replicates. This number was chosen based on accepted guidelines within the scientific community and is designed to balance the need for robust, reproducible data with considerations of experimental efficiency.

### Analysis of published data sets

All external data sets used in Supplementary Fig. 14 were downloaded from GEO GSE93724, and published by Senchuk et al.^52^ and Chen et al.^53^. Expression levels were determined by aligning the raw sequencing data to the Repbase^73^, a *C. elegans*-specific TE database, using custom Python scripts.

### Reporting summary

Further information on research design is available in the Nature Portfolio Reporting Summary linked to this article.

### Supplementary information

Supplementary Information

Reporting Summary

### Source data

Source Data

## Data Availability

All data supporting the findings of this study are provided within the paper and its Supplementary Information. Source data are provided with this paper. Any data are available from the authors upon request. Raw SMRT-sequencing reads of the 1 day old and 5 days old animals are available in the NCBI Sequence Read Archive (SRA) database with the BioProject accession code PRJNA682481. All data, code, and materials used in the analysis are available upon reasonable request for collaborative studies regulated by materials/data transfer agreements (MTA/DTAs) to the corresponding author (vellai.tibor@ttk.elte.hu). Uncropped versions of every picture used in the study can be downloaded from the link below: https://osf.io/c9wxj/?view_only=b67f2ae5eb9a4f9abbe0c5baa563dedb. Source data are provided with this paper.

## References

[1] McClintock B (1950). The origin and behaviour of mutable loci in maize. Proc. Natl Acad. Sci. USA.

[2] Levin HL, Moran JV (2011). Dynamic interactions between transposable elements and their hosts. Nat. Rev. Genet..

[3] Lander ES (2001). Initial sequencing and analysis of the human genome. Nature.

[4] Brouha B (2003). L1s accounts for the bulk retrotransposition in the human population. Proc. Natl Acad. Sci. USA.

[5] Hormozdiari F (2011). Alu repeat discovery and characterization within human genomes. Genome Res..

[6] Gasior SL, Wakeman TP, Xu B, Deininger PL (2006). The human LINE-1 retrotransposon creates DNA double-strand breaks. J. Mol. Biol..

[7] Daskalos A (2009). Hypomethylation of retrotransposable elements correlates with genomic instability in non-small cell lung cancer. Int. J. Cancer.

[8] Belgnaoui SM, Gosden RG, Semmes OJ, Haoudi A (2006). Human LINE-1 retrotransposon induces DNA damage and apoptosis in cancer cells. Cancer Cell Int..

[9] Hedges DJ, Deininger PL (2007). Inviting instability: transposable elements, double-strand breaks, and the maintenance of genome integrity. Mutat. Res..

[10] Vijg J, Dong X (2020). Pathogenic mechanisms of somatic mutation and genome mosaicism in aging. Cell.

[11] Vagin VV (2006). A distinct small RNA pathway silences selfish genetic elements in the germline. Science.

[12] Ross RJ, Weiner MM, Lin H (2014). PIWI proteins and PIWI-interacting RNAs in the soma. Nature.

[13] Murray V (1990). Are transposons a cause of aging?. Mutat. Res..

[14] Sturm Á, Ivics Z, Vellai T (2015). The mechanism of aging: primary role of transposable elements in genome disintegration. Cell. Mol. Life. Sci..

[15] Sturm Á, Perczel A, Ivics Z, Vellai T (2017). The Piwi-piRNA pathway: road to immortality. Aging Cell.

[16] Gorbunova V (2021). The role of retrotransposable elements in ageing and age-associated diseases. Nature.

[17] Egilmez NK, Shmookler Reis RJ (1994). Age-dependent somatic excision of transposable element Tc1 in Caenorhabditis elegans. Mutat. Res..

[18] Li W (2013). Activation of transposable elements during aging and neuronal decline in Drosophila. Nat. Neurosci..

[19] De Cecco M (2013). Transposable elements become active and mobile in the genomes of aging mammalian somatic tissues. Aging (Albany NY).

[20] Patterson MN (2015). Preferential retrotransposition in aging yeast mother cells is correlated with increased genome instability. DNA Repair (Amst.)..

[21] Chen H, Zheng X, Xiao D, Zheng Y (2016). Age-associated derepression of retrotransposons in the Drosophila fat body, its potential cause and consequence. Aging Cell.

[22] Wood JG (2017). Chromatin-modifying genetic interventions suppress age-associated transposable element activation and extend life span in Drosophila. Proc. Natl Acad. Sci. USA.

[23] O’Donnell KA, Burns KH (2010). Mobilizing diversity: transposable element insertions in genetic variation and disease. Mob. DNA.

[24] Van Meter M (2014). SIRT6 represses LINE1 retrotransposons by ribosylating KAP1 but this repression fails with stress and age. Nat. Commun..

[25] De Cecco M (2019). L1 drives IFN in senescent cells and promotes age-associated inflammation. Nature.

[26] Bessereau, J.-L. *Transposons in C. elegans*, WormBook (The C. elegans Research Community, WormBook, 2016).

[27] Sturm Á, Saskői É, Kovács T, Weinhardt N, Vellai T (2018). Highly efficient RNAi and Cas9-based auto-cloning systems for *C. elegans* research. Nucleic Acids Res..

[28] Bouallègue M, Rouault JD, Hua-Van A, Makni M, Capy P (2017). Molecular evolution of piggyBac superfamily: from selfishness to domestication. Genome Biol. Evol..

[29] Plasterk RH (1987). Differences between Tc1 elements from the C. elegans strain Bergerac. Nucleic Acids Res..

[30] Johnson TE, Wood WB (1982). Genetic analysis of life-span in Caenorhabditis elegans. Proc. Natl Acad. Sci. USA.

[31] Surzycki SA, Belknap WR (2000). Repetitive DNA elements are similarly distributed on Caenorhabditis autosomes. Proc. Natl Acad. Sci. USA.

[32] Tóth KF, Pezic D, Stuwe E, Webster A (2016). The piRNA pathway guards the germline genome against transposable elements. Adv. Exp. Med. Biol..

[33] Ninova M, Griffiths-Jones S, Ronshaugen M (2017). Abundant expression of somatic transposon-derived piRNAs throughout Tribolium castaneum embryogenesis. Genome Biol..

[34] Cox DN (1998). A novel class of evolutionarily conserved genes defined by piwi are essential for stem cell self-renewal. Genes Dev..

[35] Vastenhouw NL (2003). A genome-wide screen identifies 27 genes involved in transposon silencing in C. elegans. Curr. Biol..

[36] Greer EL (2015). DNA Methylation on N6-Adenine in C. elegans. Cell.

[37] Zhang G (2015). N6-methyladenine DNA modification in Drosophila. Cell.

[38] Wang SY (2019). The demethylase NMAD-1 regulates DNA replication and repair in the Caenorhabditis elegans germline. PLoS Genet..

[39] Wu TP (2016). DNA methylation on N6-adenine in mammalian embryonic stem cells. Nature.

[40] O’Brown ZK (2019). Sources of artifact in measurements of 6mA and 4mC abundance in eukaryotic genomic DNA. BMC Genomics.

[41] Schiffers S (2017). Quantitative LC-MS provides no evidence for m6dA or m4dC in the genome of mouse embryonic stem cells and tissues. Angew. Chem. Int. Ed. Engl..

[42] Douvlataniotis K, Bensberg M, Lentini A, Gylemo B, Nestor CE (2020). No evidence for DNA N 6-methyladenine in mammals. Sci. Adv..

[43] Kong Y (2022). Critical assessment of DNA adenine methylation in eukaryotes using quantitative deconvolution. Science.

[44] Yao B (2017). DNA N6-methyladenine is dynamically regulated in the mouse brain following environmental stress. Nat. Commun..

[45] Lieberman Greer E, Becker B, Latza C, Antebi A, Shi Y (2016). Mutation of C. elegans demethylase spr-5 extends transgenerational longevity. Cell Res..

[46] Kenyon C, Chang J, Gensch E, Rudner A, Tabtiang RA (1993). C. elegans mutant that lives twice as long as wild type. Nature.

[47] Senchuk MM (2018). Activation of DAF-16/FOXO by reactive oxygen species contributes to longevity in long-lived mitochondrial mutants in Caenorhabditis elegans. PLoS Genet..

[48] Flusberg BA (2010). Direct detection of DNA methylation during single-molecule, real-time sequencing. Nat. Methods.

[49] Zevian SC, Yanowitz JL (2014). Methodological considerations for heat shock of the nematode Caenorhabditis elegans. Methods.

[50] Hsu A-L, Murphy CT, Kenyon C (2003). Regulation of aging and age-related disease by DAF-16 and heat-shock factor. Science.

[51] Papp D, Csermely P, Soti C (2012). A role for SKN-1/Nrf in pathogen resistance and immunosenescence in Caenorhabditis elegans. PLoS Pathog..

[52] Lund J (2002). Transcriptional profile of aging in C. elegans. Curr. Biol..

[53] Chen AT-Y (2015). Longevity genes revealed by integrative analysis of isoform-specific daf-16/Foxo mutants of Caenorhabditis elegans. Genetics.

[54] Siddiqi S, Terry M, Matushansky I (2012). Hiwi mediated tumorigenesis is associated with DNA hypermethylation. PLoS ONE.

[55] Jardim SS, Schuch AP, Pereira CM, Loreto ELS (2015). Effects of heat and UV radiation on the mobilization of transposon mariner-Mos1. Cell Stress Chaperones.

[56] Sun L (2020). Heat stress-induced transposon activation correlates with 3D chromatin organization rearrangement in Arabidopsis. Nat. Commun..

[57] Horvath V, Merenciano M, González J (2017). Revisiting the relationship between transposable elements and the eukaryotic stress response. Trends Genet..

[58] de Oliveira DS, Rosa MT, Vieira C, Loreto ELS (2021). Oxidative and radiation stress induces transposable element transcription in Drosophila melanogaster. J. Evol. Biol..

[59] El-Brolosy MA (2019). Genetic compensation triggered by mutant mRNA degradation. Nature.

[60] Billes V (2018). Developmentally regulated autophagy is required for eye formation in Drosophila. Autophagy.

[61] Boulias K, Greer EL (2022). Means, mechanisms and consequences of adenine methylation in DNA. Nat. Rev. Genet..

[62] Valli A, Harris AL, Kessler BM (2015). Hypoxia metabolism in ageing. Aging (Albany NY).

[63] Snyder B, Wu H-K, Tillman B, Floyd TF (2022). Aged mouse hippocampus exhibits signs of chronic hypoxia and an impaired HIF-controlled response to acute hypoxic exposures. Cells.

[64] Yoder JA, Walsh CP, Bestor TH (1997). Cytosine methylation and the ecology of intragenomic parasites. Trends Genet..

[65] Sturm A, Vellai T (2022). How does maternal age affect genomic stability in the offspring?. Aging Cell.

[66] Deniz Ö, Frost JM, Branco MR (2019). Regulation of transposable elements by DNA modifications. Nat. Rev. Genet..

[67] Kenyon C (2010). The genetics of aging. Nature.

[68] Vellai T (2021). How the amino acid leucine activates the key cell-growth regulator mTOR. Nature.

[69] Hotzi B (2018). Sex-specific regulation of aging in Caenorhabditis elegans. Aging Cell.

[70] Kutnyánszky V (2020). Sex-specific regulation of neuronal functions in Caenorhabditis elegans: the sex-determining TRA-1 represses goa-1/Gα(_i/o_). Mol. Genet. Genomics.

[71] Vellai T (2003). Genetics: Influence of TOR Kinase on Lifespan in C. elegans. Nature.

[72] Berezikov E, Bargmann CI, Plasterk RH (2004). Homologous gene targeting in Caenorhabditis elegans by biolistic transformation. Nucleic Acids Res..

[73] Bao W, Kojima KK, Kohany O (2015). Repbase Update, a database of repetitive elements in eukaryotic genomes. Mob. DNA.

