# Supplementary material for: Downregulation of transposable elements extends lifespan in Caenorhabditis elegans
Source: Nat Commun. 2023 Aug 29;14:5278. doi: 10.1038/s41467-023-40957-9 (PMC10465613; doi:10.1038/s41467-023-40957-9)
Supplement: Supplementary file 1 — Supplementary Information [file 41467_2023_40957_MOESM1_ESM.pdf]

Downregulation of transposable elements extends lifespan in *Caenorhabditis elegans*

Ádám Sturm, Éva Saskői, Bernadette Hotzi, Anna Tarnóci, János Barna, Ferenc Bodnár, Himani Sharma, Tibor Kovács, Eszter Ari, Nóra Weinhardt, Csaba Kerepesi, András Perczel, Zoltán Ivics & Tibor Vellai

Supplementary Figures 1-15 and Figure Legends 1-15, Supplementary Tables 1-8.

Supplementary Figure 1

**DNA transposons (9.4% of the genome)**			
Active elements	*name*	*size (bp)*	*copy number*
	Tc1	1610	32
	Tc2	2074	4 (+ up to 300 remnants)
	Tc3	2337	19 (+ 4 truncated)
	Tc4 (no ORF)	1600	5
	Tc4v	2343	5
	Tc5 [mobile only in *mut-2(-)* background]	3171	4
	Tc7 (no ORF – uses Tc1A transposase)	921	12
	CemaT1	1281	12
Inactive elements	*name*	*size (bp)*	*copy number*
	Tc3-Cella	2166	5 (+ 5 truncated)
	Tc3-Cellb	2165	1 (+ 2 truncated)
	Tc6	1603	20-30
	Tc8	7568	154 (+ 128 truncated)
	Tc9	1600	1 (+ 15 truncated)
	Tc10	1600	3 (+ 15 truncated)
	mle1	1330	15-20
**MITEs (Miniature Inverted-repeat Transposable Elements; up to 2%of the genome) - inactive**			
family	*name*	*size (bp)*	*copy number*
PIF/harbinger	Cele7	360	300
	PAL3A_CE	150	100
Tc1/mariner	Cele1	330	1000
	Cele2	325	1500
	Cele4	470	300
	Cele6	160	100
	PALTA1_CE	146	300
	PALTA2_CE	153	50
	PALTA4_CE	198	20
	TIR54TA1_CE	200	100
Tc1-like	CeleTc1/Tc7	920	30
	Tc6	1600	30
	NPALTA1_CE	173	50-100
Tc2-like	Cele11	220	25
	Cele12	370	50
	CeleTc2	210	150
Tc5-like	CeleTc5	500-1400	15
piggyBac/TTAA	PALTTAA1_CE	592	100
	PALTTAA2_CE	174	100
Mirage	PALTTAA3_CE	594	50
	PALNN1_CE	210	20
	NPAL0A_CE	286	20-30
Merlin/IS1016	PAL8C_CE	200-350	>100
Unclassified	Cele42	240	600
	Cele14	180	2000
	PALTTTAAA1_CE	680	50
	PALTTTAAA2_CE	680	50

Suppl. Fig. 1 Catalogue of the characterised *C. elegans* transposable elements (TEs). TEs constitute approximately 12% of the *C. elegans* genome. Only the better characterized TE families are included. The activity, size and copy number of these TEs are indicated. Light blue colour indicates the main TE classes.

Supplementary Figure 2

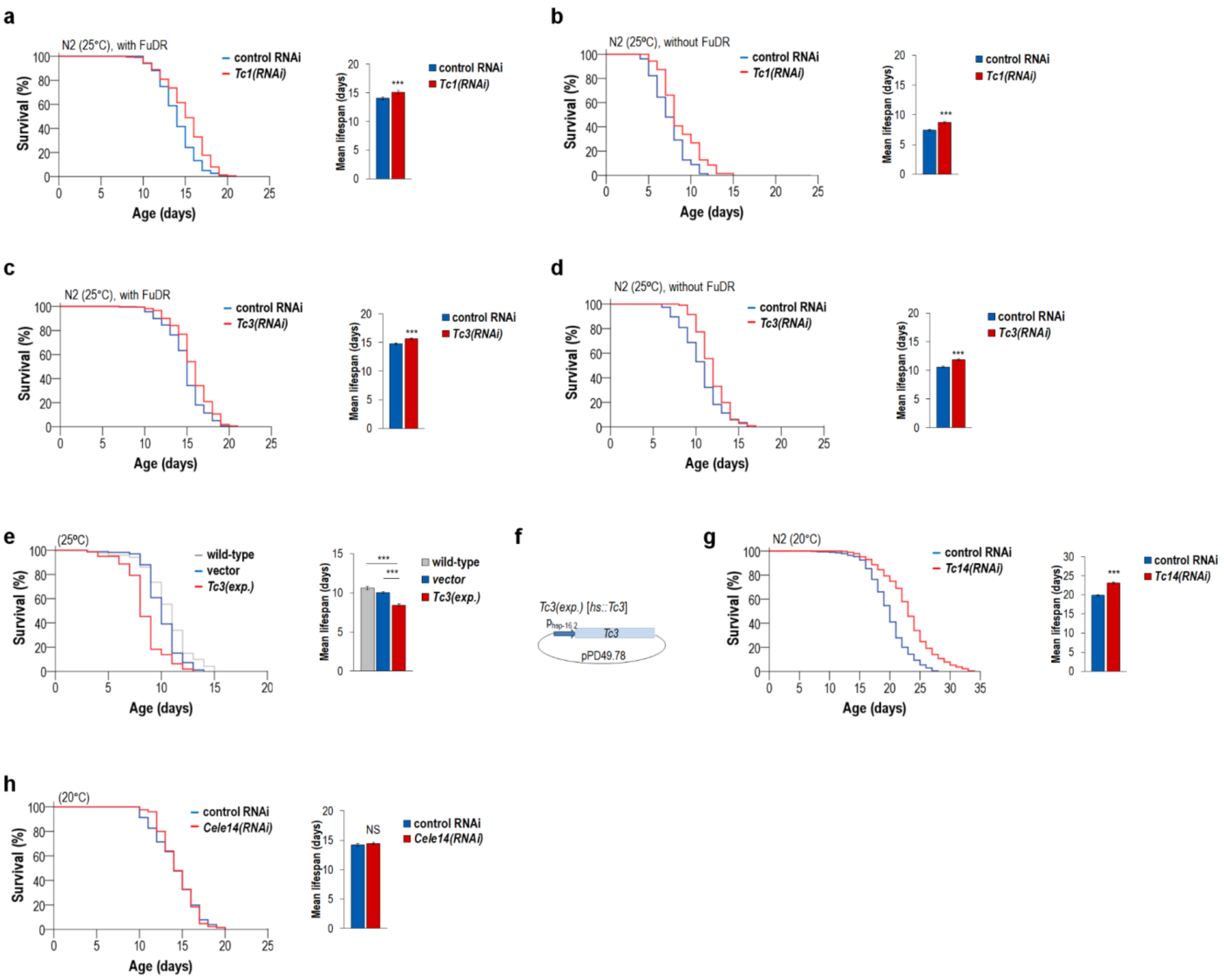

Suppl. Fig. 2 Downregulation of transposable elements extends lifespan. **a** An independent assay showing that at 25°C, animals downregulated for *Tc1* (red curve) live longer than control (blue curve) (left panel). Mean lifespan of control (treated with the empty vector T444T only) versus *Tc1(RNAi)* animals (right panel). **b** *Tc1* downregulation extends lifespan even in the absence of FuDR (left). Red curve indicates *Tc1(RNAi)* animals, the blue curve indicates control nematodes. Mean lifespan of control versus *Tc1(RNAi)* animals (right). **c** An independent assay showing that at 25°C, animals downregulated for *Tc3* (red curve) live longer than control (blue curve) (left). Mean lifespan of control (treated with the empty vector T444T only – blue column) versus *Tc3(RNAi)* animals (red column) (right). **d** *Tc3* downregulation also extends

lifespan when animals are not exposed to FuDR (left). Red curve indicates *Tc3(RNAi)* animals, the blue curve indicates control nematodes. Mean lifespan of control versus *Tc3(RNAi)* animals (right). **e** Lifespan curves of wild-type, control (expressing the empty vector pPD49.78 only) and *Tc3*-overexpressing animals (left). Animals were maintained at 25°C where the promoter is leaky. Mean lifespan data of wild-type, control versus *hs::Tc3* animals (right). **f** Structure of *hs::Tc3* construct. *Tc3* coding region is driven by *hsp-16.2* promoter. **g** An independent assay showing that at 20°C, *Tc14(RNAi)* animals (red curve) live longer than control (blue curve) (left). Mean lifespan of control (treated with the empty vector T444T only – blue column) versus *Tc14(RNAi)* animals (red column) (right). **h** Lifespan curves of control and *Cele14* downregulated animals (left). Control animals were fed with bacteria expressing the empty RNAi vector only. Mean lifespan of control versus *Cele14(RNAi)* animals (right). Kaplan-Meier lifespan curves are shown. On diagrams, bars indicate ±S.E.M; ***: $P<0.001$, independent two-sample Student's two-sided *t*-test with Bonferroni correction. For statistics, see Supplementary Tables 1 and 2.

Supplementary Figure 3

a

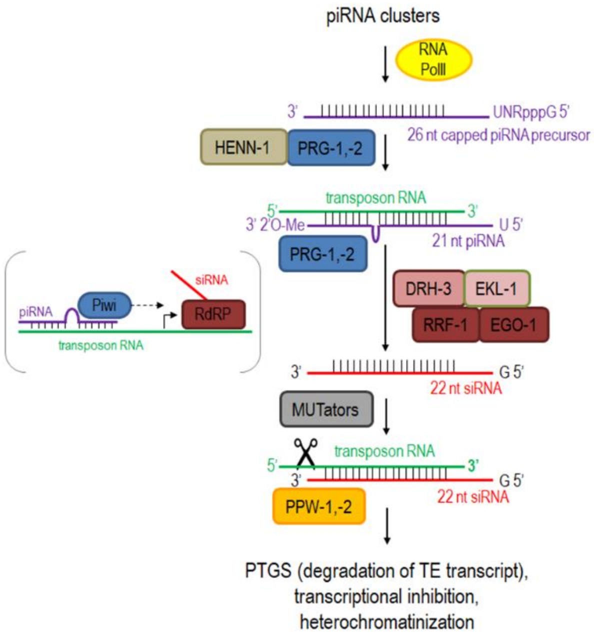

b

Gene	Somatic exp.	Germline exp.
prg-1/Piwi	-	+
prg-2/Piwi	-	+
henn-1	+	+
drh-3	+	+
ekl-1		
rrf-1	+	
ego-1		+
mut-7	+	+
rde-2		+
rde-3/mut-2		+
ppw-1/Ago/WAGO		+
ppw-2/Ago/WAGO	+	

Suppl. Fig. 3 The *C. elegans* Piwi-piRNA pathway. **a** Components of the Piwi-piRNA pathway in *C. elegans*. Nematode orthologues of *Drosophila*/mammalian Piwi-piRNAi pathway components are as follows. PRG-1, -2 (piwi related gene): Piwi; HENN-1 (HEN1 - RNA 3'end methyltransferase of Nematode): Methyltransferase; MUT-7 (mutator), RDE-2, -3 (MUT-2) (RNAi defective): Mutators; DRH-3 (dicer related helicase): Dicer-related helicase; EKL-1 (enhancer of *ksr-1* lethality): Tudor; RRF-1 (RNS-dependent RNA polymerase family), EGO-1 (enhancer of *glp*-one): RNA-dependent RNA Polymerase; PPW-1, -2 (PAZ/PIWI domain-containing): Argonaute/WAGO. **b** Endogenous somatic/germline expression of Piwi-piRNA pathway components. Colour codes correspond to those found in panel **a**. + mark denotes expression, - mark indicates no expression. Data were extracted from the WormBase database (http://www.wormbase.org). Many of these proteins accumulate somatically. This may explain why expressing individual Piwi proteins in the soma can confer a longevity effect.

Supplementary Figure 4

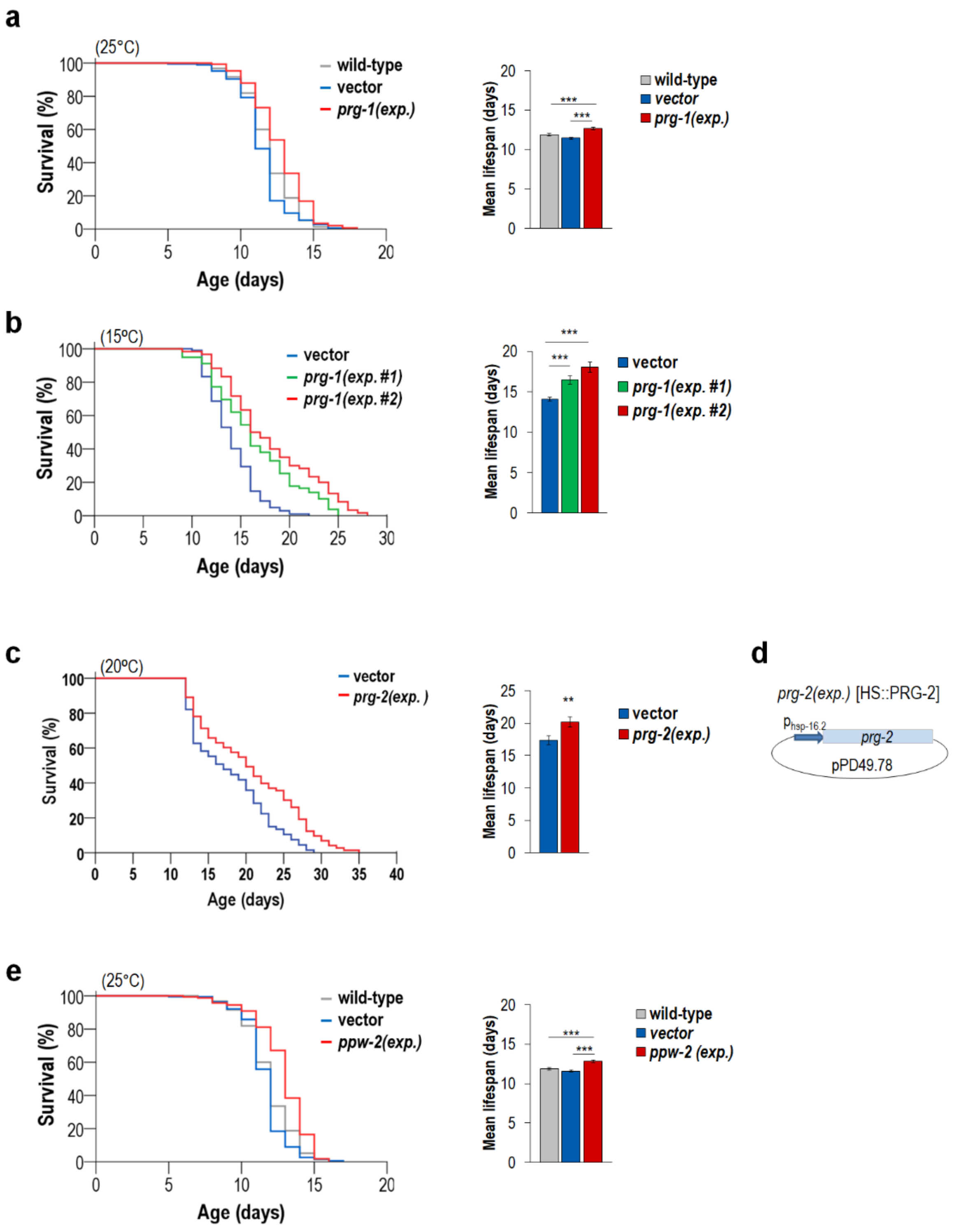

Suppl. Fig. 4 Overexpressing components of the Piwi-piRNA pathway in the soma can extend lifespan in *C. elegans*. **a** Kaplan-Meier lifespan curves of wild-type, control (containing the empty vector only) and *prg-1* overexpressing animals at 25°C (left panel). Under this condition,

somatic expression of the transgene ($p_{hsp16.2}$*::prg-1::gfp*) promote longevity. Mean lifespan of wild-type, control (animals express the empty vector) and *prg-1::gfp*-overexpressing animals (right panel). **b** Kaplan-Meier lifespan curves of animals transgenic for a $p_{hsp16.2}$*::prg-1* construct (without *gfp* reporter) at 15°C (left). Two independent integrated transgenic lines (*exp.#1* and *exp.#2*) were assayed. Animals expressing the empty vector only (vector) were used as control. Mean lifespan of animals transgenic for the empty vector (vector) and $p_{hsp16.2}$*::prg-1* construct (*exp.#1* and *exp.#2*) (right). **c** Kaplan-Meier lifespan curves of control and *prg-2*-overexpressing [*prg-2(exp.)*] animals at 20°C (left). Control animals are transgenic for the empty vector only (vector). Mean lifespan of control (vector) versus *prg-2*-overexpressing (*prg-2 exp.*) animals (right). **d** Structure of $p_{hsp16.2}$*::prg-2* construct. *prg-2* coding region is driven by *hsp-16.2* promoter ($p_{hsp16.2}$). **e** An independent assay showing that animals overexpressing *ppw-2* somatically (red curve) live longer than control (animals expressing the empty vector only – blue curve) at 25°C (left). Mean lifespan of *ppw-2*-overexpressing (red column) and control (blue column) animals (right). On diagrams, bars represent ±S.E.M.; **: $P<0.01$, ***: $P<0.001$; independent two-sample Student's two-sided *t*-tests with Bonferroni correction. For statistics, see Supplementary Table 1.

Supplementary Figure 5

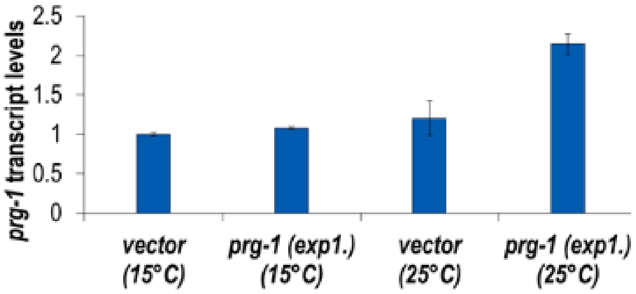

Suppl. Fig. 5 *prg-1* transcripts levels in animals transgenic for a *gfp*-free *prg-1* transgene. *prg-1* expression is highly increased under an inducing condition. Transcript levels were determined by RT-qPCR. Temperatures are indicated. *cdc-42* was used as an internal control. Bars indicate ±S.E.M., Mann-Whitney U test. For statistics, see Supplementary Table 3.

Supplementary Figure 6

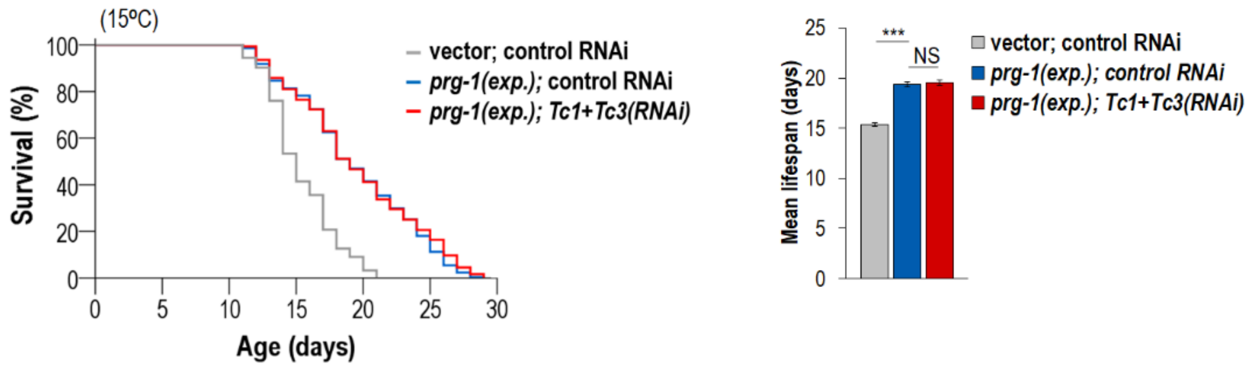

Suppl. Fig. 6 Simultaneous downregulation of *Tc1* and *Tc3* cannot further extend lifespan in transgenic animals expressing PRG-1 somatically. Left panel: Kaplan-Meier lifespan curves of control animals (grey), animals expressing *prg-1* somatically (blue) and animals expressing *prg-1* somatically and co-downregulated for *Tc1* and *Tc3* (red) at 15°C. Right panel: mean lifespan of control (grey column), *prg-1*-overexpressing (blue column) versus *prg-1*-overexpressing, *Tc1+Tc3 dsRNAi*-treated (red) animals. Bars represent ±S.E.M., ***: P< 0.001; NS: not significant; independent two-sample Student's two-sided *t*-test with Bonferroni corrections. For statistics and conditions, see Supplementary Table 1.

Supplementary Figure 7

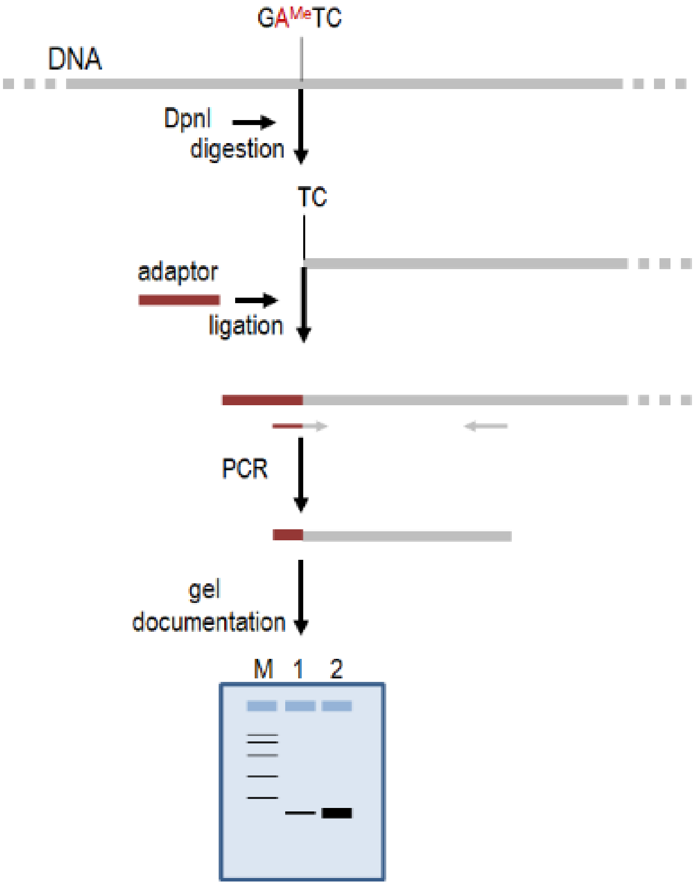

Suppl. Fig. 7 Scheme of the PCR-based method for accurately detecting N^6-methyladenine (6mA) levels at a given genomic site. Genomic DNA is digested with DpnI enzyme (it cuts the GATC target sequence when the A is methylated). An adaptor DNA (linker) is then ligated to the digested genomic DNA fragments. PCR amplification of the target DNA site is achieved by a forward primer that is specific to both linker and a short DNA sequence being adjacent to the target GATC site. This way the methylated (digested) fragments can be assayed directly. This is crucial because N^6-adenine methylation normally occurs at a very low rate in a given locus (only a very few individual genomes from a tissue sample are methylated at a given adenine nucleobase while a vast majority of individual genomes remain unmethylated at the same site), so DpnI digestion coupled with PCR amplification of the digested site alone (without linker ligation) cannot accurately quantify methylation level (PCR reaction actually amplifies the undigested – unmethylated – fragments, the excess of which makes them hardly

distinguishable from each other). PCR products are then visualised on an agarose gel (below), and normalized to an internal control that indicates the quantity of template DNA. M: molecular weight marker, 1 and 2 represent samples. The method identifies 6mA sites in a sequence-specific (PCR primer-directed) manner, thereby generating no artefact.

Supplementary Figure 8

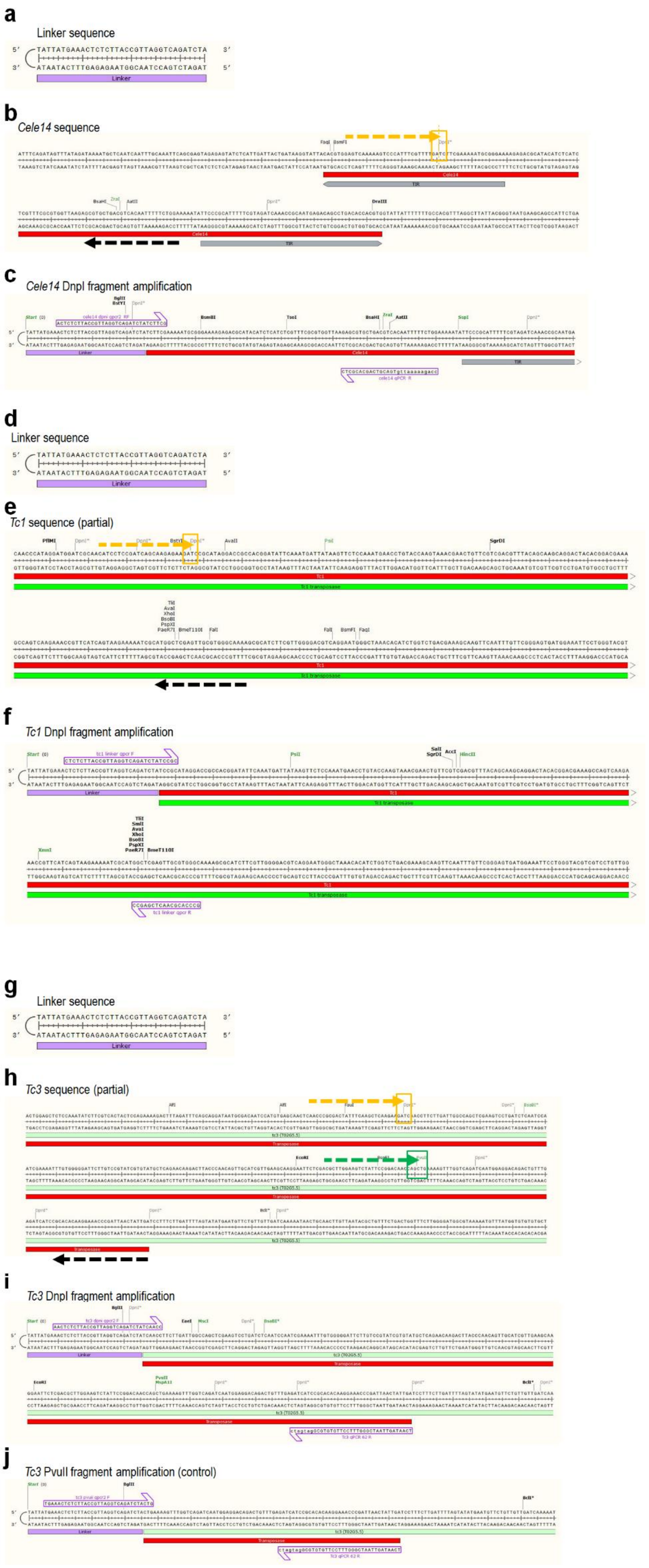

Suppl. Fig. 8 Sequence information of primers and the linker fragment for measuring relative *N*^{6}-methyladenine levels at *Cele14* sequences. **a** Linker sequence. **b** The sequence of *Cele14*. A methylated DpnI site we assayed is indicated by a yellow frame. *Cele14* stretch is indicated by a red bar, the tandem inverted repeats are indicated by grey bars. **c** Primer information (purple arrows) for the amplification of a *Cele14* fragment methylated at the given adenine position. Linker is indicated by purple colouring. Forward primer is specific to both linker and adjacent *Cele14* sequences. **d** Linker fragment. **e** Partial sequence of *Tc1*. A methylated DpnI site is indicated by a yellow frame. *Tc1* stretch is indicated by a red bar, the transposase coding region is indicated by a green bar. **f** Primer information (purple arrows) for the amplification of a *Tc1* fragment methylated at the given adenine. Linker is indicated by purple colouring. Forward primer is specific to both linker and adjacent *Tc1* sequences. **g** Linker sequence. **h** A partial sequence of *Tc3*. A methylated DpnI site is indicated by a yellow frame, a PvuII site (control) is indicated by a green frame. *Tc3* sequence is indicated by a light blue bar, the transposase coding region is shown by a red bar. **i** Primer information (purple arrows) for the amplification of a *Tc3* fragment methylated at the given adenine. Linker is indicated by purple colouring. Forward primer is specific to both linker and adjacent *Tc1* sequences. **j** Primer information (purple arrows) for the amplification of a *Tc3* fragment at a given PvuII site (control fragment). Restriction enzyme sites are shown.

Supplementary Figure 9

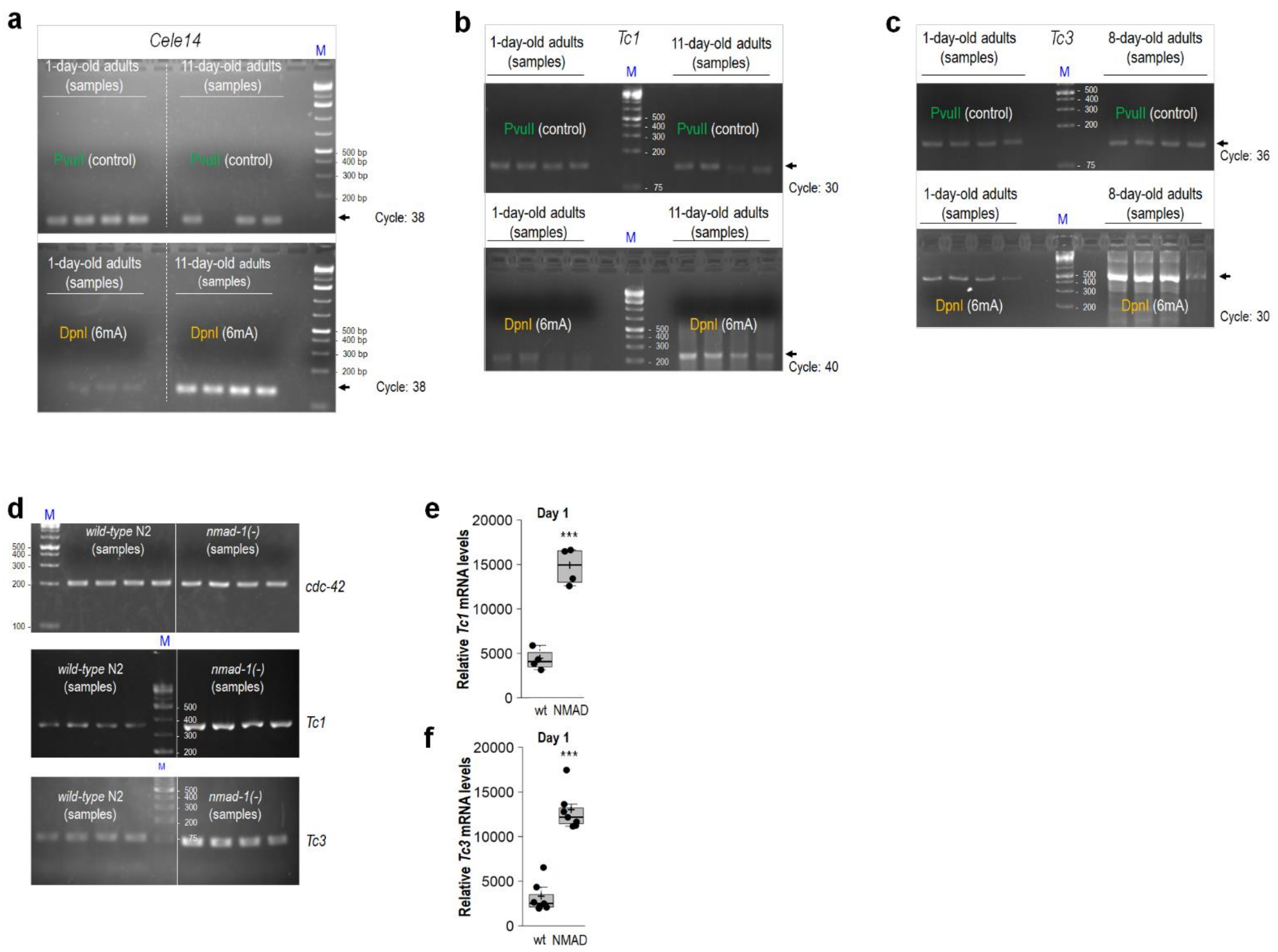

Suppl. Fig. 9 N^6-methyladenine (6mA) levels at transposable element (TE) sequences increase with age. **a** Relative 6mA levels at *Cele14* sequences. Upper panel: relative quantity of a *Cele14* fragment with a PvuII site (control). Similar amounts of DNA can be detected in young and old animals. Bottom panel: relative 6mA levels at a *Cele14* fragment with a methylated DpnI site. 6mA levels increase by around 5-fold in aged animals, as compared with young adults. **b** 6mA levels elevate at *Tc1* sequences during adulthood. Upper panel: relative quantity of a *Tc1* fragment with a PvuII site (control). Similar amounts of DNA can be detected in young and old adults. Bottom panel: relative 6mA levels at a *Tc1* fragment with a methylated DpnI site. 6mA levels significantly increase in old animals, as compared with young ones. **c** 6mA levels

increase at *Tc3* sequences as the animal ages. Upper panel: relative quantity of *Tc3* fragments with a PvuII site (control). Similar amounts of DNA can be detected in young versus aged animals. Bottom panel: relative 6mA levels at *Tc3* fragments with a methylated DpnI site. 6mA levels increase in old animals, as compared with young ones. DNA samples were amplified from young (1-day) and old (11-day) adult nematodes (**a**-**c**). PCR cycles are indicated; M: molecular weight marker; arrows indicate the fragments amplified (**a**-**c**). Animals were maintained at 25°C. **d** Semi-qPCR analysis demonstrating transcript levels of *cdc-42* (control, upper panel), *Tc1* (middle panel) and *Tc3* (bottom panel) in wild-type versus *nmad-1(ok3133)* mutant backgrounds. 4-4 samples were tested for each. M: molecular weight marker. **e** Relative transcript levels of *Tc1*, normalized to *cdc-42*. **f** Relative transcript levels of *Tc3*, normalized to *cdc-42*. Transcript levels were normalized to 1-day-old (young) adults, wt denotes wild-type, and bars represent ±S.E.M., $P<0.001$, independent two-sided *t*-test (**e**, **f**). For statistics, see Supplementary Table 2. Centre lines show the medians; box limits indicate the 25th and 75th percentiles as determined by R software; whiskers extend 1.5 times the interquartile range from the 25th and 75th percentiles, outliers are represented by dots; crosses represent sample means; data points are plotted as circles.

Supplementary Figure 10

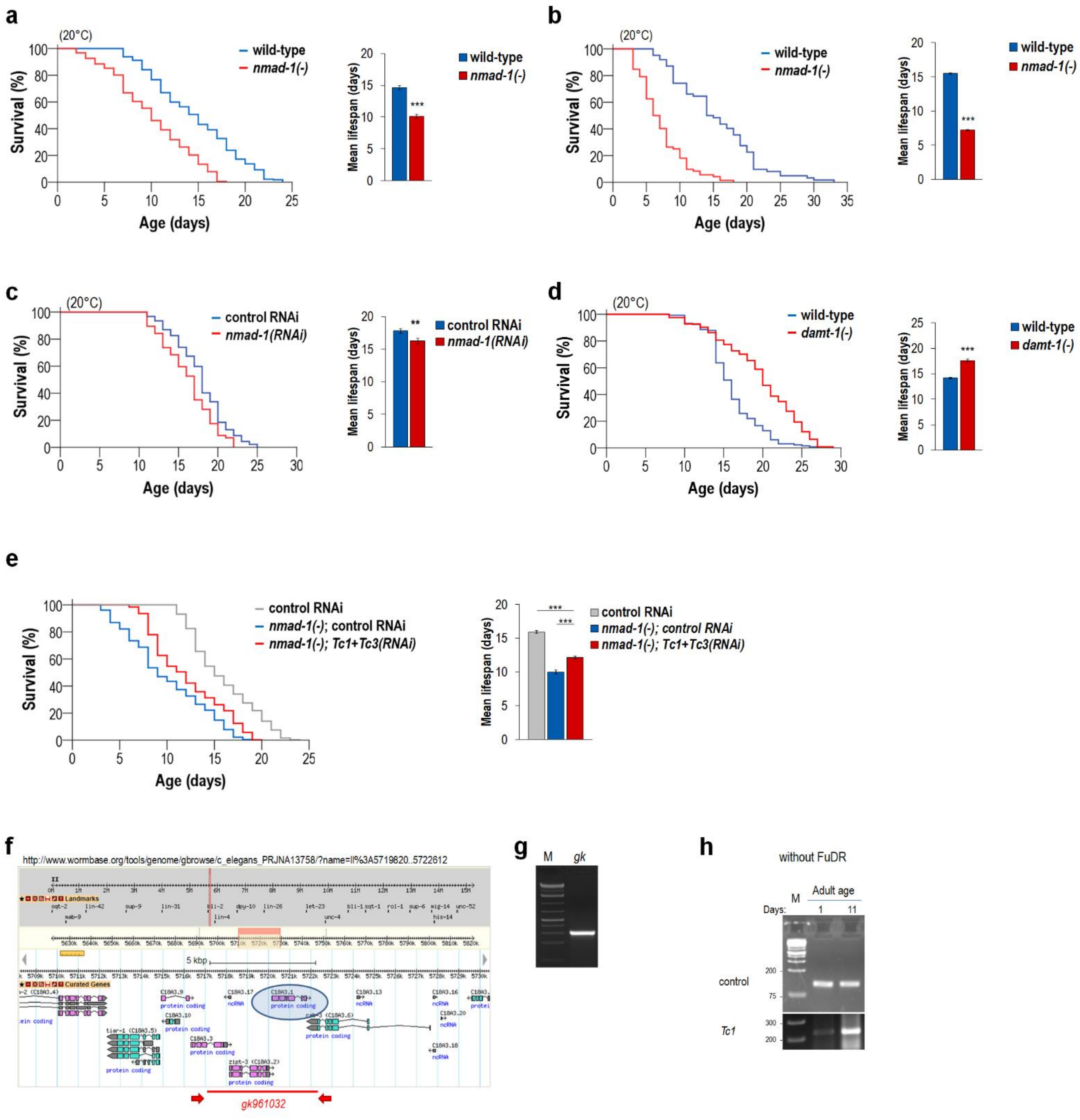

Suppl. Fig. 10 NMAD-1 deficiency shortens, while a *damt-1(-)* mutation increases, lifespan. **a** Lifespan assay showing that *nmad-1(-)* mutant animals (red) live shorter than wild-type (blue) at 20°C (left panel). Mean lifespan of mutant (red) vs. control animals (blue) (right panel). **b** Independent assay revealing the negative effect of an *nmad-1(-)* mutation on lifespan (left). The corresponding mean lifespan of mutant (red) and control (blue) animals (right). **c** *nmad-1(RNAi)*

animals (red) are short lived relative to control (blue) (left). The latter was fed with bacteria expressing the empty RNAi vector only. Mean lifespan data (right). **d** Independent assay demonstrating that a *damt-1(-)* mutation (red) extends lifespan relative to control (blue) (left). Mean lifespan of *damt-1(-)* mutant (red) vs. control (blue) animals (right). **e** Lifespan curves of *N2* control animals (grey), *nmad-1(-)* mutants (blue) and *nmad-1(-)* mutants co-downregulated for *Tc1* and *Tc3* (red) at 20°C (left). Mean lifespan data of control (grey), *nmad-1(-)* mutant (blue) and *nmad-1(-); Tc1+Tc3(RNAi)* animals (red) (right). Simultaneous downregulation of *Tc1* and *Tc3* significantly extends lifespan in *nmad-1(-)* mutant animals. Kaplan-Meier lifespan curves are shown. On diagrams, bars represent ±S.E.M., ***: $P<0.001$; Independent two-sample Student's two-sided *t*-test with Bonferroni corrections. For statistics, see Supplementary Table 1. **f** *damt-1(gk961032)* mutation is a large deletion. Physical map of *damt-1/C18A3.1* locus (http://www.wormbase.org). *C18A3.1* open reading frame (ORF) is highlighted by a blue background (circle). The horizontal red line (bottom) indicates the extent of the *gk961032* deletion. The mutant allele overlaps with the following ORFs: *C18A3.3*, *C18A3.17*, *C18A3.2* (*zipt-3*), *C18A3.1* (*damt-1*) and *C18A3.6* (*rab-3*). **g** Gel electrophoretic picture of a PCR product amplifying the *gk961032* deletion-containing genomic DNA sequence (~300 bp) from *damt-1(gk961032)* mutants. M: molecular weight marker, *gk* denotes *gk961032*. The genomic annealing sites of primers used for PCR amplification are indicated by red arrows in panel (**f**). **h** N^6-methyladenine levels also increase with age at *Tc1* loci in animals not exposed to FuDR. 6mA levels were determined at adult stages of days 1 and 11. M: molecular weight marker.

Supplementary Figure 11

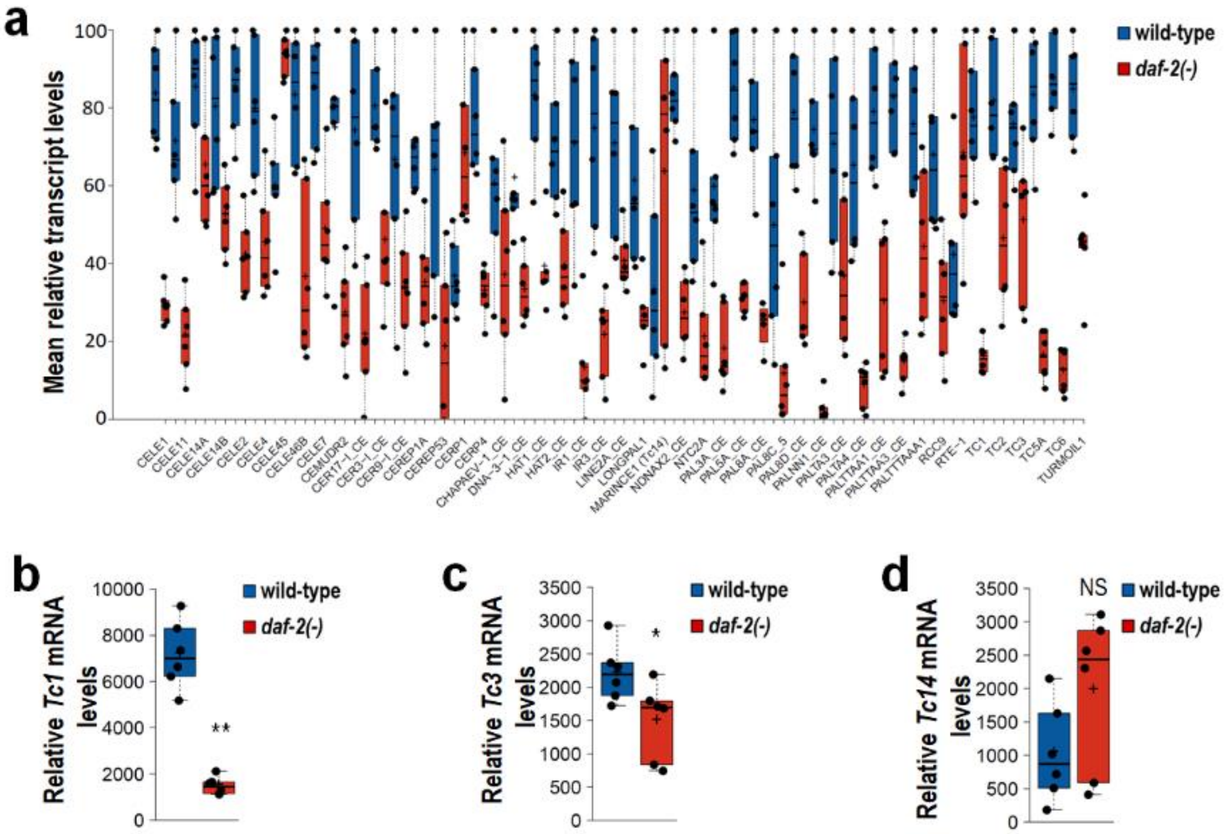

Suppl. Fig. 11 Comparing the expression of certain transposable elements (TEs) in wild-type versus *daf-2(e1370)* mutant animals. Bioinformatics analysis was performed on available RNA-sequencing (RNAseq) data[46]. **a** Mean expression levels of TEs where the difference in expression was significant (P<0.05) between wild-type and *daf-2(-)* mutant animals. Quantities are normalized to the highest value (100). Out of the 180 TE families examined, 46 displayed a significant expression difference when comparing 1 old wild-type and *daf-2(-)* mutant adults. Out of these 46 TE families, 39 were overexpressed in the wild type compared with *daf-2(-)* mutants. **b** Relative expression of *Tc1* in wild-type and *daf-2(-)* mutant animals. **c** Relative expression levels of *Tc3* in wild-type and *daf-2(-)* mutant animals. **d** Expression levels of *Tc14* in wild-type versus *daf-2(-)* mutant backgrounds. At this early adult stages, *Tc14* transcript levels were not changed (shown as NS) in the mutant genetic background. Thus, the cumulative

expression and mobilization of a total of thousands of members of the 180 TE families, rather than a single TE family, in *C. elegans* may significantly affect lifespan. Thus, a significant portion of TE families are less expressed in *daf-2(-)* mutants, and this may be the reason why these animals live longer. WT denotes wild-type, and TE expressions in wild-type animals and *daf-2(-)* mutants are shown by blue and red colourings, respectively. Bar indicates ±S.E.M., *: P<0.05, **: P<0.01, NS: not significant; independent two-sided *t*-test. For statistics, see Supplementary Table 5. Centre lines show the medians; box limits indicate the 25th and 75th percentiles as determined by R software; whiskers extend 1.5 times the interquartile range from the 25th and 75th percentiles, outliers are represented by dots; crosses represent sample means; data points are plotted as circles.

Supplementary Figure 12

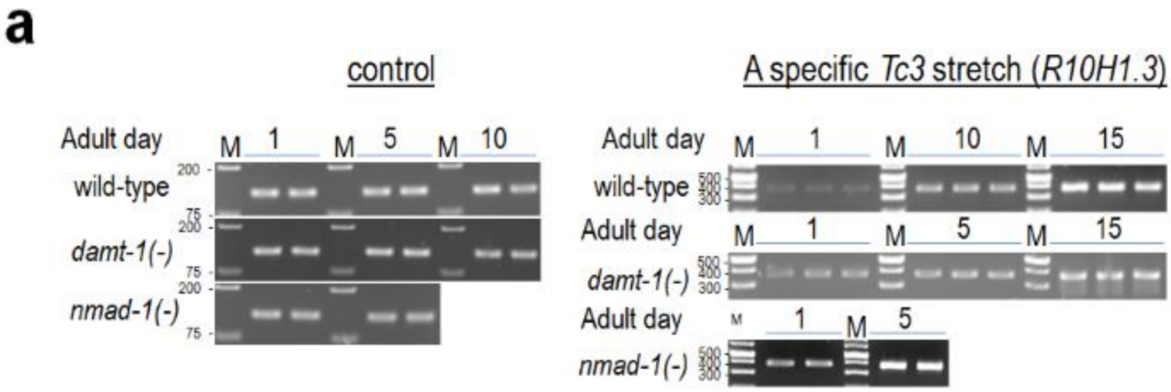

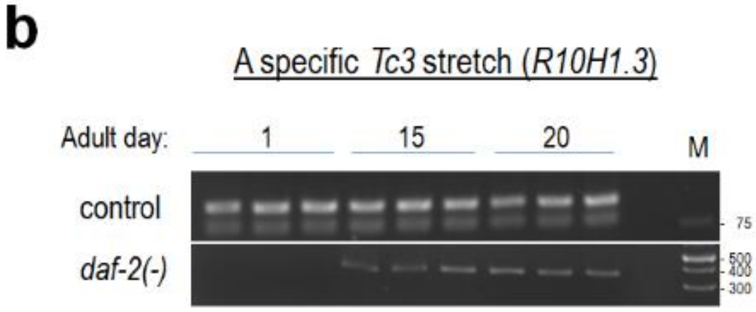

Suppl. Fig. 12 N^6-adenine methylation at an individual *Tc3* stretch (*R10H1.3*) promotes the excision of the element. **a** Control samples showing equal amounts of genomic DNA at different stages of adulthood (left) and the excision rate of *R10H1.3* in wild-type versus *damt-1(-)* and *nmad-1(-)* mutant animals at different adult stages (right). Excision increases with age in each genotype examined, but its rate is higher in mutants defective for NMAD-1 function and lower in mutants deficient in DMAT-1 function, as compared with wild-type. **b** Excision rate of *R10H1.3* in long-lived *daf-2(-)* mutants at different adult stages. Days at which semi-qPCR tests were performed are indicated for each genotype (**a**-**b**). 2 or 3 replicates were used. M: molecular weight marker (fragment sizes are given in base pair).

Supplementary Figure 13

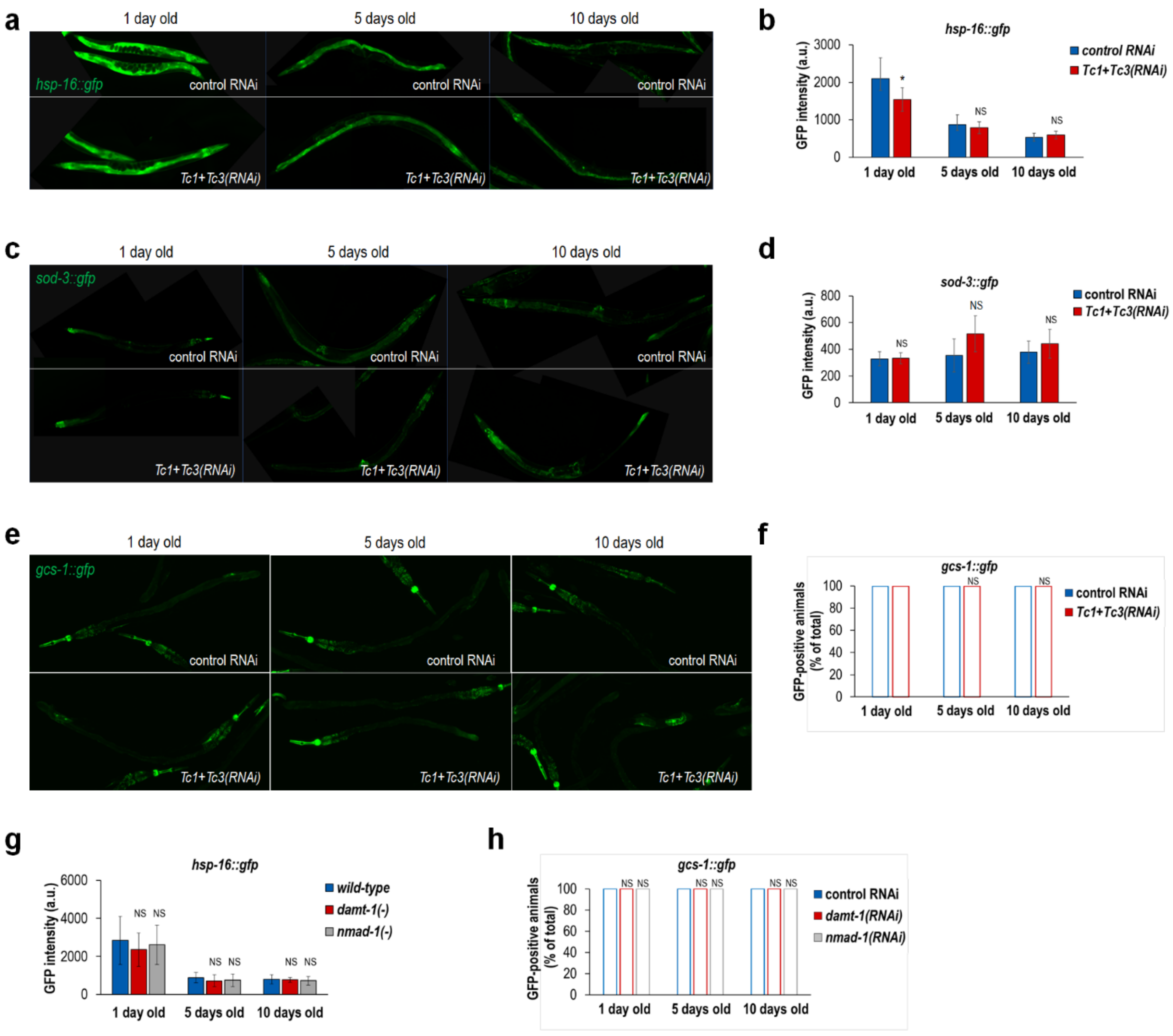

Suppl. Fig. 13 Downregulation of transposable elements (TEs) does not influence the expression of *hsp-16.2*, *sod-3* and *gcs-1*, which are target genes of different stress response pathways. **a** *hsp-16.2* expression levels are similar between control and *Tc1+Tc3(RNAi)* animals at different adult stages. *hsp-16.2* is a target gene of the heat shock response pathway. **b** Quantification of *hsp-16.2::gfp* expression. **c** Expression of a *sod-3::gfp* reporter is independent of *Tc1* and *Tc3* activity at each adult stage examined. *sod-3* is a target gene of the nutritional stress response pathway (mediated by the DAF-2 – DAF-16 signalling axis). **d**

Quantification of *sod-3::gfp* expression in control versus *Tc1+Tc3(RNAi)* animals at different adult stages. **e** The activity of *Tc1* and *Tc3* does not influence the expression of a *gcs-1::gfp* reporter. *gcs-1* is a target gene of the oxidative stress-response pathway. Under normal conditions, only a background expression is visible in the head. **f** Quantification of *gcs-1::gfp* expression in control versus *Tc1+Tc3(RNAi)* background at different adult stages. **g** Expression of a *hsp-16.2::gfp* reporter in wild-type versus *damt-1(-)* and *nmad-1(-)* mutant animals at different adult stages. No significant change is detectable among the genotypes. **h** Expression of a *gcs-1::gfp* reporter in control (wild-type), *damt-1(RNAi)* and *nmad-1(RNAi)* animals. No significant alteration is visible among the genotypes. Representative fluorescent images of control (untreated) versus *Tc1+Tc3(RNAi)* animals at different adult stages (**a**, **c**, **e**). On diagrams, bars indicate ±S.E.M., NS: not significant; independent two-sided *t*-test. For statistics, see Supplementary Table 7.

Supplementary Figure 14

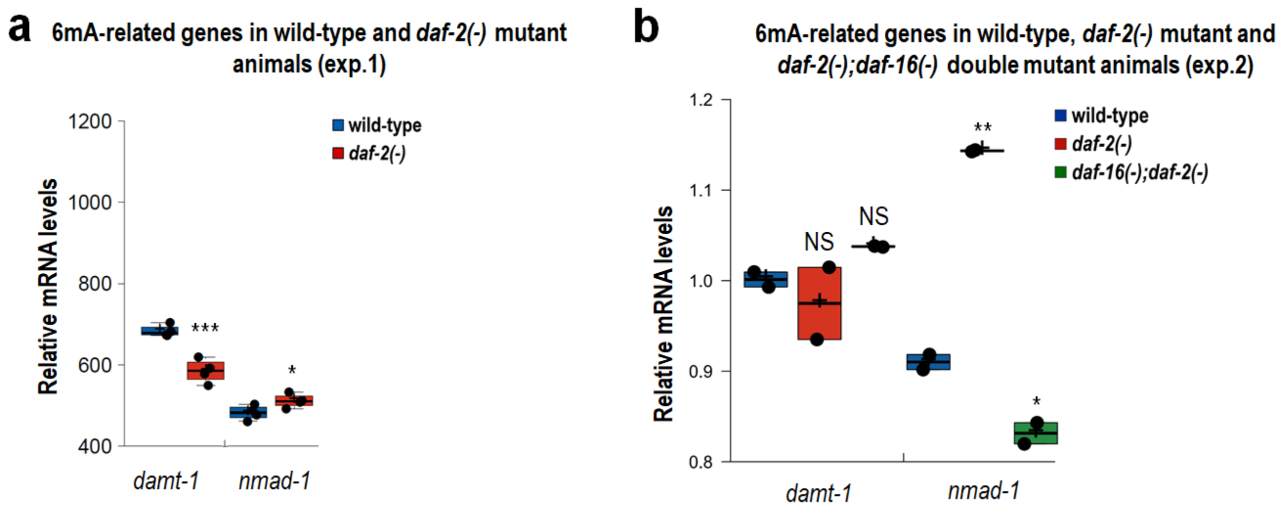

Suppl. Fig. 14 DAF-2 activity causes repression of *damt-1*, but activation of *nmad-1* in a DAF-16-dependent manner. **a** Expression of *damt-1* is decreased, whereas *nmad-1* transcription levels increase, in *daf-2(-)* mutant animals relative to wild-type. Available RNAseq data[53] were analysed. **b** Expression of *damt-1* tends to be lowered, whereas *nmad-1* transcription levels increase, in *daf-2(-)* mutants compared with wild-type. These changes are effectively suppressed by DAF-16 deficiency. Another set of freely available RNAseq data[54] was analysed. The transcription factor DAF-16 acts as the effector of the DAF-2/IGF-1 signalling pathway. *: $P<0.05$, ***: $P<0.001$; NS: not significant; independent two-sided *t*-test. For statistics, see Supplementary Table 8. Centre lines show the medians; box limits indicate the 25th and 75th percentiles as determined by R software; whiskers extend 1.5 times the interquartile range from the 25th and 75th percentiles, outliers are represented by dots; crosses represent sample means; data points are plotted as circles.

Supplementary Figure 15

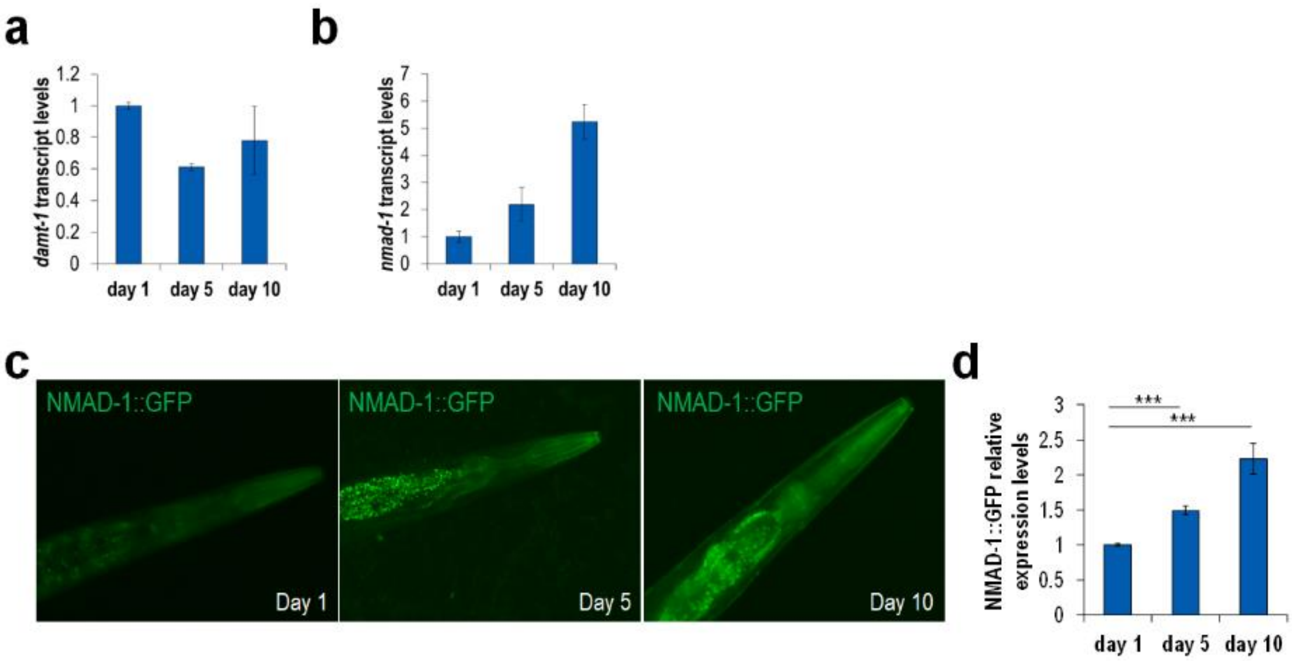

Suppl. Fig. 15 Expression of *damt-1* remains nearly constant, whereas *nmad-1* transcript and NMAD-1 protein levels increase, during the adult lifespan. **a** Transcript levels of *damt-1* at different adult stages. **b** Transcript levels of *nmad-1* at different adult stages. RT-qPCR analysis and bars indicates ±S.E.M. (**a**, **b**). **c** Accumulation levels of a NMAD-1::GFP translational fusion reporter protein at different stages of adulthood. Expression intensities in the head were determined by fluorescence microscopy. **d** Quantification of NMAD-1::GFP signals in the head. Images were captured with the same exposure time. Bars indicates ±S.E.M., ***: P<0.001, independent two-sided *t*-test. For statistics, see Supplementary Tables 3 and 7.

Supplementary Tables

Supplementary Table 1 Statistics for lifespan data generated in this study. WT denotes the wild-type genetic background; BO indicates the mutator strain Bergerac. BO animals were maintained at 15°C only, at higher temperatures animals become highly fragile and sterile. Where there is no indication, genetic manipulation took place in an otherwise wild-type genetic background. exp. indicates somatic overexpression from a heat-shock inducible promoter (*hsp-16.2*) that is active in almost all somatic cells. Lifespan experiments were repeated at least two independent times with similar results and the data for representative experiments are shown. *At least 50 worms per condition were examined in each independent experiment. Animals that climbed up the wall of plastic dishes or died prematurely because of picking were censored from the analysis. Nematodes on the plates contaminated by microorganisms were also excluded from the analysis. Supplementary Table 1 shows the observed number out of the total number of animals.

**Genotype** (temperature at which silencing was performed)	**Indep. exp.**	**Observed number of worms***	**% Lifespan increase in independent experiments**	**Log Rank P value (with Bonferroni correction)**	**Mean lifespan (days)**	**±SEM (days)**	**% Increase in mean lifespan**	**Log Rank P value (with Bonferroni correction)**	**Independent *t*-test P value (with Bonferroni correction)**
for **Fig. 1a**									
WT control (RNAi vector) (15°C)	#1	65			22.25	0.20			
	#2	85							
WT *Tc1(RNAi)* (15°C)	#1	52	vs. control (RNAi vector) #1 **+7.28%**	vs. control (RNAi vector) #1 P=0.0007	23.55	0.24	vs. control (RNAi vector) **+5.84%**	vs. control (RNAi vector) P<0.0001	vs. control (RNAi vector) P<0.0001
	#2	84	vs. control (RNAi vector) #2 **+4.08%**	vs. control (RNAi vector) #2 P=0.0010					
for **Fig. 1b**									
WT control (RNAi vector) (25°C)	#1	84			14.22	0.22			
	#2	42							
WT *Tc1(RNAi)* (25°C)	#1	95	vs. control (RNAi vector) #1 **+6.21%**	vs. control (RNAi vector) #1 P=0.0060	15.15	0.24	vs. control (RNAi vector) **+6.54%**	vs. control (RNAi vector) P=0.0007	vs. control (RNAi vector) P=0.0052
	#2	47	vs. control (RNAi vector) #2 **+6.76%**	vs. control (RNAi vector) #2 P=0.0550					
for **Fig. 1c**									
BO control (RNAi vector) (15°C)	#1	55			20.28	0.41			
	#2	63							
BO	#1	71	vs. control	vs. control	22.16	0.46			vs. control

Tc1(RNAi)<br>(15°C)			(RNAi vector) #1<br>**+7.95%**	(RNAi vector) #1<br>P=0.0526			vs. control<br>(RNAi vector)<br>**+9.47%**	vs. control<br>(RNAi vector)<br>P=0.0003	(RNAi vector)<br>P=0.0031
	#2	71	vs. control<br>(RNAi vector) #2<br>**+11.70%**	vs. control<br>(RNAi vector) #2<br>P=0.0024					
for **Fig. 1d**									
WT<br>control (RNAi vector)<br>(25°C)	#1	84			14.22	0.22			
	#2	42							
WT<br>*Tc3(RNAi)*<br>(25°C)	#1	85	vs. control<br>(RNAi vector) #1<br>**+8.18%**	vs. control<br>(RNAi vector) #1<br>P=0.0023	15.38	0.23	vs. control<br>(RNAi vector)<br>**+8.16%**	vs. control<br>(RNAi vector)<br>P=0.0003	vs. control (RNAi vector)<br>P=0.0003
	#2	43	vs. control<br>(RNAi vector) #2<br>**+7.75%**	vs. control<br>(RNAi vector) #2<br>P=0.0529					
for **Fig. 1e**									
WT<br>control (RNAi vector)<br>(20°C)	#1	48			21.63	0.34			
WT<br>*Tc14(RNAi)*<br>(20°C)	#1	46	vs. control<br>(RNAi vector) #1<br>**+10.96%**	vs. control<br>(RNAi vector) #1<br>P=0.0001	24.00	0.54	vs. control<br>(RNAi vector)<br>**+10.96%**	vs. control<br>(RNAi vector)<br>P=0.0001	vs. control<br>(RNAi vector)<br>P=0.0003
WT<br>control (RNAi vector)<br>(20°C)	#2	95			21.24	0.29			
WT<br>*Tc14(RNAi)*<br>(20°C)	#2	93	vs. control<br>(RNAi vector) #2<br>**+11.97%**	vs. control<br>(RNAi vector) #2<br>P<0.0001	23.79	0.39	vs. control<br>(RNAi vector)<br>**+11.97%**	vs. control<br>(RNAi vector)<br>P<0.0001	vs. control<br>(RNAi vector)<br>P<0.0001
for **Fig. 2a**									
WT<br>control (RNAi vector)<br>(20°C)	#1	48			21.63	0.34			
WT<br>*Tc1(RNAi)*<br>(20°C)	#1	44	vs. control<br>(RNAi vector) #1<br>**+5.69%**	vs. control<br>(RNAi vector) #1<br>P=0.0222	22.86	0.44	vs. control<br>(RNAi vector)<br>**+5.69%**	vs. control<br>(RNAi vector)<br>P=0.0222	vs. control<br>(RNAi vector)<br>P=0.0271
WT<br>control (RNAi vector)<br>(20°C)	#2	95			21.32	0.25			
WT<br>*Tc1(RNAi)*<br>(20°C)	#2	87	vs. control<br>(RNAi vector) #2<br>**+5.04%**	vs. control<br>(RNAi vector) #2<br>P=0.0047	22.39	0.29	vs. control<br>(RNAi vector)<br>**+5.04%**	vs. control<br>(RNAi vector)<br>P=0.0047	vs. control<br>(RNAi vector)<br>P=0.0050
for **Fig. 2b**									
WT<br>control (RNAi vector)<br>(20°C)	#1	48			21.63	0.34			
WT<br>*Tc3(RNAi)*<br>(20°C)	#1	49	vs. control<br>(RNAi vector) #1<br>**+6.70%**	vs. control<br>(RNAi vector) #1<br>P=0.0081	23.08	0.32	vs. control<br>(RNAi vector)<br>**+6.70%**	vs. control<br>(RNAi vector)<br>P=0.0081	vs. control<br>(RNAi vector)<br>P=0.0024
WT<br>control (RNAi vector)<br>(20°C)	#2	95			21.32	0.25			
WT<br>*Tc3(RNAi)*<br>(20°C)	#2	97	vs. control<br>(RNAi vector) #2<br>**+6.84%**	vs. control<br>(RNAi vector) #2<br>P<0.0001	22.77	0.22	vs. control<br>(RNAi vector)<br>**+6.84%**	vs. control<br>(RNAi vector)<br>P<0.0001	vs. control<br>(RNAi vector)<br>P<0.0001
for **Fig. 2c**									
WT<br>control (RNAi vector)<br>(20°C)	#1	48			21.63	0.34			
WT<br>*Tc1+Tc3(RNAi)*<br>(20°C)	#1	54	vs. control<br>(RNAi vector) #1<br>**+16.74%**	vs. control<br>(RNAi vector) #1<br>P<0.0001	25.25	0.39	vs. control<br>(RNAi vector)<br>**+16.74%**	vs. control<br>(RNAi vector)<br>P<0.0001	vs. control<br>(RNAi vector)<br>P<0.0001
WT<br>control (RNAi vector)<br>(20°C)	#2	95			21.32	0.25			
WT<br>*Tc1+Tc3(RNAi)*<br>(20°C)	#2	103	vs. control<br>(RNAi vector) #2<br>**+16.24%**	vs. control<br>(RNAi vector) #2<br>P<0.0001	24.78	0.28	vs. control<br>(RNAi vector)<br>**+16.24%**	vs. control<br>(RNAi vector)<br>P<0.0001	vs. control<br>(RNAi vector)<br>P<0.0001
for **Fig. 3a**									
wild-type<br>(20°C)	#1	60	vs. *prg-1(exp1.)* #1<br>**+34.28%**	vs. *prg-1(exp1.)* #1<br>P<0.0001	14.00	0.44	vs. *prg-1(exp1.)*<br>**+34.28%**	vs. *prg-1(exp1.)*<br>P<0.0001	vs. *prg-1(exp1.)*<br>P<0.0001

control<br>(vector pPD118.26)<br>(20°C)	#1	56			14.21	0.38			
prg-1(exp.)<br>(20°C)	#1	56	vs. control<br>(vector) #1<br>**+32.30%**	vs. control<br>(vector) #1<br>P<0.0001	18.80	0.60	vs. control<br>(vector)<br>**+32.30%**	vs. control<br>(vector)<br>P<0.0001	vs. control<br>(vector)<br>P<0.0001
wild-type<br>(20°C)	#2	96	vs. *prg-1(exp1.)* #2<br>**+20.68%**	vs. *prg-1(exp1.)* #2<br>P<0.0001	13.49	0.21	vs. *prg-1(exp1.)*<br>**+20.68%**	vs. *prg-1(exp1.)*<br>P<0.0001	vs. *prg-1(exp1.)*<br>P<0.0001
control<br>(vector pPD118.26)<br>(20°C)	#2	89			13.70	0.25			
prg-1(exp.)<br>(20°C)	#2	82	vs. control<br>(vector) #2<br>**+18.86%**	vs. control<br>(vector) #2<br>P<0.0001	16.28	0.47	vs. control<br>(vector)<br>**+18.86%**	vs. control<br>(vector)<br>P<0.0001	vs. control<br>(vector)<br>P<0.0001
for **Fig. 3d**									
wild-type<br>(25°C)	#1	85	vs. *prg-1(exp1.)* #1<br>**+16.12%**	vs. *prg-1(exp1.)* #1<br>P<0.0001	10.63	0.11	vs. *prg-1 (exp1.)*<br>**+14.48%**	vs. *prg-1 (exp1.)*<br>P<0.0001	vs. *prg-1 (exp1.)*<br>P<0.0001
	#2	66	vs. *prg-1(exp1.)* #2<br>**+11.46%**	vs. *prg-1(exp1.)* #2<br>P<0.0001					
control<br>(vector pPD49.78)<br>(25°C)	#1	69			10.65	0.11			
	#2	79							
prg-1(exp1.)<br>(25°C)	#1	68	vs. control<br>(vector) #1<br>**+15.24%**	vs. control<br>(vector) #1<br>P<0.0001	12.17	0.13	vs. control<br>(vector)<br>**+14.27%**	vs. control<br>(vector)<br>P<0.0001	vs. control<br>(vector)<br>P<0.0001
	#2	80	vs. control<br>(vector) #2<br>**+10.67%**	vs. control<br>(vector) #2<br>P<0.0001					
for **Fig. 3g**									
wild-type<br>(25°C)	#1	54	vs. *ppw-2(exp.)* #1<br>**+12.63%**	vs. *ppw-2(exp.)* #1<br>P=0.0081	11.83	0.30	vs. *ppw-2(exp.)*<br>**+12.63%**	vs. *ppw-2(exp.)*<br>P=0.0081	vs. *ppw-2(exp.)*<br>P<0.0001
control<br>(vector pPD49.78)<br>(25°C)	#1	59			12.05	0.16			
ppw-2(exp.)<br>(25°C)	#1	64	vs. control<br>(vector) #1<br>**+10.60%**	vs. control<br>(vector) #1<br>P<0.0001	13.33	0.14	vs. control<br>(vector)<br>**+10.60%**	vs. control<br>(vector)<br>P<0.0001	vs. control<br>(vector)<br>P<0.0001
for **Fig. 5g**									
wild-type<br>(20°C)	#1	110			13.86	0.35			
nmad-1(-)<br>(20°C)	#1	109	vs. wild-type #1<br>**-37.52%**	vs. *wild-type* #1<br>P<0.0001	8.66	0.43	vs. *wild-type*<br>**-37.52%**	vs. *wild-type*<br>P<0.0001	vs. *wild-type*<br>P<0.0001
for **Fig. 5h**									
wild-type<br>*(20°C)*	#1	141			14.26	0.22			
damt-1(gk961032)<br>*(20°C)*	#1	103	vs. *wild-type* #1<br>**+22.93%**	vs. *wild-type* #1<br>P<0.0001	17.53	0.30	vs. *wild-type*<br>**+22.93%**	vs. *wild-type*<br>P<0.0001	vs. *wild-type*<br>P<0.0001
for **Fig. 5i**									
WT<br>control (RNAi vector)<br>(20°C)	#1	84			14.22	0.22			
	#2	42							
WT<br>*damt-1(RNAi)*<br>(20°C)	#1	67	vs. control<br>(RNAi vector) #1<br>**+8.24%**	vs. control<br>(RNAi vector) #1<br>P=0.0078	15.47	0.26	vs. control<br>(RNAi vector)<br>**+8.79%**	vs. control<br>(RNAi vector)<br>P=0.0005	vs. control<br>(RNAi vector)<br>P=0.0003
	#2	40	vs. control<br>(RNAi vector) #2<br>**+9.19%**	vs. control<br>(RNAi vector) #2<br>P=0.0299					
for S**upplementary Fig. S2a**									
WT<br>control (RNAi vector)<br>(25°C)<br>with FuDR	#1	219			14.04	0.15			
WT<br>*Tc1(RNAi)*<br>(25°C)<br>with FuDR	#1	288	vs. control<br>(RNAi vector) #1<br>**+7.34%**	vs. control<br>(RNAi vector) #1<br>P<0.0001	15.07	0.15	vs. control<br>(RNAi vector)<br>**+7.34%**	vs. control<br>(RNAi vector)<br>P<0.0001	vs. control<br>(RNAi vector)<br>P<0.0001
for S**upplementary Fig. S2b**									
WT	#1	79			7.43	0.22			

control (RNAi vector)<br>(25°C)<br>without FuDR									
WT<br>*Tc1(RNAi)*<br>(25°C)<br>without FuDR	#1	71	vs. control<br>(RNAi vector) #1<br>**+17.50%**	vs. control<br>(RNAi vector) #1<br>P=0.0003	8.73	0.27	vs. control<br>(RNAi vector)<br>**+17.50%**	vs. control<br>(RNAi vector)<br>P=0.0003	vs. control<br>(RNAi vector)<br>P=0.0002
for S**upplementary Fig. S2c**									
WT<br>control (RNAi vector)<br>(25°C)<br>with FuDR	#1	316			14.78	0.13			
WT<br>*Tc3(RNAi)*<br>(25°C)<br>with FuDR	#1	320	vs. control<br>(RNAi vector) #1<br>**+5.98%**	vs. control<br>(RNAi vector) #1<br>P<0.0001	15.66	0.13	vs. control<br>(RNAi vector)<br>**+5.98%**	vs. control<br>(RNAi vector)<br>P<0.0001	vs. control<br>(RNAi vector)<br>P<0.0001
for S**upplementary Fig. S2d**									
WT<br>control (RNAi vector)<br>(25°C)<br>without FuDR	#1	115			10.61	0.22			
WT<br>*Tc3(RNAi)*<br>(25°C)<br>without FuDR	#1	106	vs. control<br>(RNAi vector) #1<br>**+11.78%**	vs. control<br>(RNAi vector) #1<br>P=0.0011	11.86	0.17	vs. control<br>(RNAi vector)<br>**+11.78%**	vs. control<br>(RNAi vector)<br>P=0.0011	vs. control<br>(RNAi vector)<br>P<0.0001
for **Supplementary Fig. S2e**									
wild-type<br>(25°C)	#1	70	vs. *Tc3(exp.)* #1<br>**-22.24%**	vs. *Tc3(exp.)* #1<br>P<0.0001	10.59	0.21	vs. *Tc3(exp.)*<br>**-20.49%**	vs. *Tc3(exp.)*<br>P<0.0001	vs. *Tc3(exp.)*<br>P<0.0001
	#2	51	vs. *Tc3(exp.)* #2<br>**-17.86%**	vs. *Tc3(exp.)* #2<br>P<0.0001					
control<br>(vector pPD49.78)<br>(25°C)	#1	108			10.02	0.13			
	#2	58							
Tc3(exp.)<br>(25°C)	#1	96	vs. control<br>(vector) #1<br>**-18.25%**	vs. control<br>(vector) #1<br>P<0.0001	8.42	0.15	vs. control<br>(vector)<br>**-15.97%**	vs. control<br>(vector)<br>P<0.0001	vs. control<br>(vector)<br>P<0.0001
	#2	63	vs. control<br>(vector) #2<br>**-12.53%**	vs. control<br>(vector) #2<br>P=0.0012					
for **Supplementary Fig. S2g**									
WT<br>control (RNAi vector)<br>(20°C)	#3	136			18.31	0.29			
WT<br>*Tc14(RNAi)*<br>(20°C)	#3	133	vs. control<br>(RNAi vector) #3<br>**+21.25%**	vs. control<br>(RNAi vector) #3<br>P<0.0001	22.20	0.39	vs. control<br>(RNAi vector)<br>**+21.25%**	vs. control<br>(RNAi vector)<br>P<0.0001	vs. control<br>(RNAi vector)<br>P<0.0001
for **Supplementary Fig. S2h**									
WT<br>control (RNAi vector)<br>(20°C)	#1	84			14.22	0.22			
	#2	42							
WT<br>*Cele14(RNAi)*<br>(20°C)	#1	79	vs. control<br>(RNAi vector) #1<br>**+1.78%**	vs. control<br>(RNAi vector) #1<br>P=0.9037	14.46	0.19	vs. control<br>(RNAi vector)<br>**+1.69%**	vs. control<br>(RNAi vector)<br>P=0.9393	vs. control<br>(RNAi vector)<br>P=0.4200
	#2	46	vs. control<br>(RNAi vector) #2<br>**+1.03%**	vs. control<br>(RNAi vector) #2<br>P=0.9303					
for **Supplementary Fig. S4a**									
wild-type<br>(25°C)	#1	54	vs. *prg-1(exp.)* #1<br>**+2.94%**	vs. *prg-1(exp.)* #1<br>P=1.0000	11.88	0.14	vs. *prg-1(exp.)*<br>**+6.57%**	vs. *prg-1(exp.)*<br>P=0.0001	vs. *prg-1(exp.)*<br>P=0.0002
	#2	101	vs. *prg-1(exp.)* #2<br>**+8.61%**	vs. *prg-1(exp.)* #2<br>P<0.0001					
control<br>(vector pPD118.26)<br>(25°C)	#1	57			11.46	0.12			
	#2	131							
prg-1(exp.)<br>(25°C)	#1	55	vs. control<br>(vector) #1<br>**+5.05%**	vs. control<br>(vector) #1<br>P=0.8280	12.66	0.15	vs. control<br>(vector)<br>**+10.47%**	vs. control<br>(vector)<br>P<0.0001	vs. control<br>(vector)<br>P<0.0001
	#2	94	vs. control<br>(vector) #2	vs. control<br>(vector) #2					

			**+13.43%**	P<0.0001					
for **Supplementary Fig. S4b**									
control (vector pPD49.78) (15°C)	#1	60			14.07	0.24			
	#2	42							
prg-1(exp. #1) (15°C)	#1	49	vs. control (vector) #1 **+18.48%**	vs. control (vector) #1 P<0.0001	16.44	0.49	vs. control (vector) **+16.84%**	vs. control (vector) P<0.0001	vs. control (vector) P<0.0001
	#2	30	vs. control (vector) #2 **+14.61%**	vs. control (vector) #2 P=0.0080					
prg-1(exp. #2) (15°C)	#1	35	vs. control (vector) #1 **+23.12%**	vs. control (vector) #1 P<0.0001	18.00	0.62	vs. control (vector) **+27.93%**	vs. control (vector) P<0.0001	vs. control (vector) P<0.0001
	#2	25	vs. control (vector) #2 **+34.46%**	vs. control (vector) #2 P<0.0001					
for **Supplementary Fig. S4c**									
control (vector pPD49.78) (20°C)	#1	34			17.82	0.64			
	#2	33							
prg-2(exp.) (20°C)	#1	37	vs. control (vector) #1 **+9.61%**	vs. control (vector) #1 P=0.2013	20.59	0.78	vs. control (vector) **+15.54%**	vs. control (vector) P=0.0013	vs. control (vector) P=0.0075
	#2	36	vs. control (vector) #2 **+21.22%**	vs. control (vector) #2 P=0.0014					
for **Supplementary Fig. S4e**									
wild-type (25°C)	#2	155	vs. *ppw-2(exp.)*#1 **+8.08%**	vs. *ppw-2(exp.)#1* P<0.0001	11.88	0.14	vs. *ppw-2(exp.)* **+8.08%**	vs. *ppw-2(exp.)* P<0.0001	vs. *ppw-2(exp.)* P<0.0001
control (vector pPD49.78) (25°C)	#2	190			11.61	0.11			
ppw-2(exp.) (25°C)	#2	164	vs. control (vector) #1 **+10.59%**	vs. control (vector) #1 P<0.0001	12.84	0.14	vs. control (vector) **+10.59%**	vs. control (vector) P<0.0001	vs. control (vector) P<0.0001
for **Supplementary Fig. S6**									
control (vector pPD49.78); control RNAi (25°C)	#1	309	vs. *prg-1(exp1.)*; *Tc1+Tc3(RNAi)* #1 **+27.00%**	vs. *prg-1(exp1.)*; *Tc1+Tc3(RNAi)* #1 P<0.0001	15.37	0.15	vs. *prg-1(exp1.)*; *Tc1+Tc3(RNAi)* **+27.00%**	vs. *prg-1(exp1.)*; *Tc1+Tc3(RNAi)* P<0.0001	vs. *prg-1(exp1.)*; *Tc1+Tc3(RNAi)* P<0.0001
prg-1(exp1.); control RNAi (25°C)	#1	294	vs. control*;* control RNAi #1 **+26.02%**	vs. control*;* control RNAi #1 P<0.0001	19.37	0.27	vs. control*;* control RNAi #1 **+26.02%**	vs. control*;* control RNAi P<0.0001	vs. control*;* control RNAi P<0.0001
prg-1(exp1.); *Tc1+Tc3(RNAi)* (25°C)	#1	311	vs. *prg-1(exp1.);* control RNAi #1 **+0.77%**	vs. *prg-1(exp1.);* control RNAi #1 P=0.8316	19.52	0.27	vs. *prg-1(exp1.);* control RNAi **+0.77%**	vs. *prg-1(exp1.);* control RNAi P=0.8316	vs. *prg-1(exp1.);* control RNAi P=1.0000
for **Supplementary Fig. S10a**									
wild-type (20°C)	#2	117			14.63	0.31			
nmad-1(-) (20°C)	#2	108	vs. *wild-type* #2 **-30.90%**	vs. *wild-type* #2 P<0.0001	10.11	0.29	vs. *wild-type* **-30.90%**	vs. *wild-type* P<0.0001	vs. *wild-type* P<0.0001
for **Supplementary Fig. S10b**									
wild-type (20°C)	#3	62			15.48	0.79			
nmad-1(-) (20°C)	#3	72	vs. *wild-type* #3 **-53.36%**	vs. *wild-type #3* P<0.0001	7.22	0.41	vs. *wild-type* **-53.36%**	vs. *wild-type* P<0.0001	vs. *wild-type* P<0.0001
for **Supplementary Fig. S10c**									
WT control (RNAi vector) (20°C)	#1	60			17.82	0.35			
	#2	32							
nmad-1(RNAi) (20°C)	#1	31	vs. control (RNAi vector) #1 **-9.48%**	vs. control (RNAi vector) #1 P=0.0167	16.25	0.43	vs. control (RNAi vector) **-8.81%**	vs. control (RNAi vector) P=0.0043	vs. control (RNAi vector) P=0.0055
	#2	26	vs. control (RNAi vector) #2 **-8.12%**	vs. control (RNAi vector) #2 P=0.1119					

for **Supplementary Fig. S10d**									
wild-type *(20°C)*	#2	132			14.16	0.18			
damt-1(gk961032) *(20°C)*	#2	124	vs. *wild-type* #2 **+24.44%**	vs. *wild-type* #2 P<0.0001	17.62	0.28	vs. wild-type **+24.44%**	vs. wild-type P<0.0001	vs. wild-type P<0.0001
for **Supplementary Fig. S10e**									
control RNAi (25°C)	#1	320	vs. *nmad-1(-); Tc1+Tc3(RNAi)*#1 **-23.76%**	vs. *nmad-1(-); Tc1+Tc3(RNAi)*#1 P<0.0001	15.91	0.16	vs. *nmad-1(-); Tc1+Tc3(RNAi)* **-23.76%**	vs. *nmad-1(-); Tc1+Tc3(RNAi)* P<0.0001	vs. *nmad-1(-); Tc1+Tc3(RNAi)* P<0.0001
nmad-1(-); control RNAi (25°C)	#1	230	vs. control RNAi #1 **-37.46%**	vs. control RNAi #1 P<0.0001	9.95	0.29	vs. control RNAi **-37.46%**	vs. control RNAi P<0.0001	vs. control RNAi P<0.0001
nmad-1(-); *Tc1+Tc3(RNAi)* (25°C)	#1	313	vs. *nmad-1(-)*; control RNAi #1 **+21.91%**	vs. *nmad-1(-)*; control RNAi #1 P<0.0001	12.13	0.22	vs. *nmad-1(-)*; control RNAi **+21.91%**	vs. *nmad-1(-)*; control RNAi P<0.0001	vs. *nmad-1(-)*; control RNAi P<0.0001

Supplementary Table 2. Statistics for transcript levels determined by semi-qPCR**.** Data for Figs. 1, 2 and 5, and Supplementary Fig. S9d-d'', relative transcript levels were normalized to that of *cdc-42* (internal control).

**Gene**	**Background**	**Trials**	**mRNA level (band intensity)**	**±S.E.M.**	**Independent two-sided *t*-test P value vs. control**
for **Fig. 1b**					
Tc1	*wild-type*; control RNAi (15°C)	3	44001.1	7752.4	control
	wild-type; *Tc1* RNAi (15°C)	3	24583.7	7949.1	P=0.006258
for **Fig. 1f**					
Tc1	Bergerac; control RNAi (15°C)	3	5559.8	627.3	control
	Bergerac*;* *Tc1(RNAi)* 15°C)	3	3068.8	165.3	P<0.0001
for **Fig. 1d**					
Tc1	*wild-type*; control RNAi (25°C)	3	5646.3	3530.2	control
	wild-type; *Tc1(RNAi)* (25°C)	3	3190.5	3114.8	P=0.07119
for **Fig. 1h**					
Tc3	*wild-type*; control RNAi (25°C)	3	1692.4	360.9	control
	wild-type; *Tc3(RNAi)* (25°C)	3	917.0	154.2	P=0.035

for **Fig. 1j**					
Tc14	*wild-type*; control RNAi (20°C)	3	3008.5	918.3	control
	wild-type; *Tc14(RNAi)* (20°C)	3	1881.7	501.7	P=0.037
for **Fig. 2d**					
Tc1+Tc3	control RNAi	3	9421.5	1217.0	control
	Tc1+Tc3(RNAi)	3	7622.8	1069.4	P=0.013
for **Fig. 2e, f**					
cdc-42	day 1	2	2648.4	331.6	control
	day 4	2	2457.8	354.4	P=0.634
	day 7	2	2431.4	354.8	P=0.592
	day 9	2	2593.0	293.1	P=0.878
	day 12	2	2498.1	454.0	P=0.742
Tc1	day 1	2	3123.3	478.3	control
	day 4	2	3311.2	203.8	P=0.660
	day 7	2	4912.7	568.9	P=0.076
	day 9	2	5101.8	429.2	P=0.049
	day 12	2	5292.9	398.4	P=0.039
Tc3	day 1	2	3064.5	501.9	control
	day 4	2	3040.3	336.6	P=0.960
	day 7	2	4596.5	318.1	P=0.068
	day 9	2	4777.2	289.9	P=0.053
	day 12	2	4829.7	299.4	P=0.051
Tc14	day 1	2	2811.3	89.1	control
	day 4	2	2742.5	160.8	P=0.649
	day 7	2	4792.0	140.9	P=0.004
	day 9	2	4860.2	121.0	P=0.003
	day 12	2	4881.4	365.0	P=0.016
for **Fig. 5a, b-d**					
Tc1	*wild-type*	9	0,591881	0,120321	control
	nmad-1(-)	9	0,973081	0,036204	P<0.0001
Tc3	*wild-type*	9	0,307278	0,09958	control
	nmad-1(-)	9	0,865074	0,223986	P<0.0001
Tc14	*wild-type*	9	0,752211	0,180683	control
	nmad-1(-)	9	0,883465	0,138103	NS
Tc1	control RNAi	9	0,968935	0,039099	control
	damt-1(RNAi)	9	0,667706	0,128233	P<0.0001
Tc3	control RNAi	9	0,945062	0,060094	control
	damt-1(RNAi)	9	0,735837	0,164964	P=0.0025
Tc14	control RNAi	9	0,90486	0,147225	control
	damt-1(RNAi)	9	0,638618	0,124403	p=0,00076
for **Supplementary Fig. 9e** (1 day-old „young" adults)					
Tc1	*wild-type*	3	4304	1159	control
	nmad-1(-)	3	14781	2083	P=0.0005

for **Supplementary Fig. 9f** (1 day-old „young” adults)						
Tc3	*wild-type*		3	3174	1694	control
	nmad-1(-)		3	12884	2204	P<0.0001
**Gene**	**Background**	**Adult age**	**Trials**	**mRNA level (band intensity)**	**±S.D.**	**One -way ANOVA with Tukey Post Hoc Test, P value**
for **Fig. 6j**						
Tc3	*wild-type*	day 1	3	1.000	0.068	control
		day 10	3	2.699	0.040	P<0.0001
		day 15	3	4.146	0.254	P<0.0001
	damt-1 (-)	day 1	3	0.527	0.065	control
		day 5	3	0.975	0.126	P=0.0050
		day 10	3	1.653	0.117	P<0.0001
	nmad-1 (-)	day 1	3	1.701	0.125	control
		day 5	3	3.779	0.123	P<0.0001
	daf-2(-)	day 1	3	0.004	0.007	control
		day 15	3	1.000	0.650	P=0.0013
		day 20	3	2.773	0.612	P=0.0003

Supplementary Table 3 Statistics for transcript levels determined by RT-qPCR**.** Relative transcript levels were normalized to that of *cdc-42* (inner control).

Gene	Genotype	Trials	Relative transcript level	±S.E.M.	Mann-Whitney U test P value vs. control
for **Fig. 3f**					
Tc1	control (vector pPD49.78)	3	1	0.313	control
	prg-1(exp1.)	3	0.651	0.083	P=0.003
for **Fig. 3f**					
Tc3	control (vector pPD49.78)	3	1	0.173	control
	prg-1(exp1.)	3	0.392	0.067	P<0.0001
for **Fig. 3j**					
Tc1	control (vector pPD49.78)	3	1	0.293	control
	ppw-2(exp.)	3	0.737	0.135	P=0.642
for **Fig. 3j**					
Tc3	control (vector pPD49.78)	3	1	0.291	control
	ppw-2(exp.)	3	0.290	0.107	P=0.044
for **Fig. 3j**					
Tc14	control (vector pPD49.78)	3	1	0.206	control
	ppw-2(exp.)	3	0.207	0.276	P= 0.0202
for **Fig. 8c**					
Tc1	*wild-type* (20 °C)	3	1	0	control
	wild-type (heat shock, 30 min 35°C)	3	4.579	3.185	P=0.037
Tc3	*wild-type* (20 °C)	3	1	0	control
	wild-type (heat shock, 30 min 35°C)	3	2.900	1.701	P=0.037
for **Supplementary Fig. 5**					
prg-1	control (vector pPD118.26) (15°C)	2	1	0.020	control
	prg-1(exp1.) (15°C)	2	1.079	0.019	P=0.519
	control (vector pPD118.26) (25°C)	2	1.206	0.214	control
	prg-1(exp1.)	2	2.144	0.131	P<0.0001

	(25°C)				
for **Supplementary Fig. 15a, b**					
damt-1	*wild-type* (day1)	2	1	0.020	control
	wild-type (day5)	2	0.612	0.019	P<0.0001
	wild-type (day10)	2	0.780	0.214	P=0.116
nmad-1	*wild-type* (day1)	2	1	0.200	control
	wild-type (day5)	2	2.191	0.612	P=0.012
	wild-type (day10)	2	5.231	0.637	P<0.0001

Supplementary Table 4. Statistics for N^6-methyladenine (6mA) levels. TE: transposable element.

TE/gene	Adult age	Trials	Relative 6mA levels	±S.E.M.	Independent two-sided *t*-test P value
for **Fig. 4e**					
Cele14	day 1	3	100.0	8.9	control
	day 11	3	568.0	73.1	P<0.0001
for **Fig. 4f**					
Tc1	day 1	3	100.0	90.0	control
	day 11	3	443.0	209.0	P=0.010
for **Fig. 4g**					
Tc3	day 1	3	100.0	7.6	control
	day 11	3	461.8	15.0	P<0.0001

Background	TE/gene	Adult age (days)	Trials	Mean of relative 6mA level	±SD	One -way ANOVA with Tukey Post Hoc Test, P value
**for Fig. 6a-d**						
N2	*Tc1*	day 1	3	1.000	0.410	control
		day 4	3	3.363	0.115	vs. 1. day P=0.017
		day 7	3	8.896	0.932	vs. 1. day P=0.008
		day 9	3	10.414	0.826	vs. 1. day P<0.0001
		day 12	3	12.784	0.278	vs. 1. day P<0.0001
	Tc3	day 1	3	1.000	0.033	control
		day 4	3	5.243	1.310	vs. 1. day P=0.004
		day 7	3	9.068	1.373	vs. 1. day P<0.0001
		day 9	3	11.351	1.040	vs. 1. day P<0.0001
		day 12	3	13.392	0.503	vs. 1. day P<0.0001
	Tc14	day 1	3	1.000	0.323	control
		day 4	3	3.798	0.344	vs. 1. day P=0.004
		day 7	3	7.060	1.091	vs. 1. day P<0.0001
		day 9	3	10.130	0.391	vs. 1. day P<0.0001
		day 12	3	11.526	0.516	vs. 1. day P<0.0001
	cep-1	day 1	3	1.000	0.087	control
		day 4	3	1.004	0.130	vs. 1. day P=0.966
		day 7	3	0.971	0.097	vs. 1. day P=0.958
		day 9	3	0.979	0.044	vs. 1. day P=1.000
		day 12	3	0.951	0.117	vs. 1. day P=0.996
daf-2	*Tc1*	day 1	3	1.000	0.255	control
		day 7	3	3.134	0.548	vs. 1. day P=0.003
		day 12	3	6.945	0.466	vs. 1. day P<0.0001
		day 16	3	10.053	0.882	vs. 1. day P<0.0001

		day 20	3	13.166	0.536	vs. 1. day P<0.0001
	Tc3	day 1	3	1.000	0.025	control
		day 7	3	3.791	0.393	vs. 1. day P=0.083
		day 12	3	8.092	0.689	vs. 1. day P=0.0001
		day 16	3	10.718	0.717	vs. 1. day P<0.0001
		day 20	3	14.081	0.533	vs. 1. day P<0.0001
	Tc14	day 1	2	1.000	0.066	control
		day 7	2	3.761	0.461	vs. 1. day P=0.0003
		day 12	2	6.317	0.583	vs. 1. day P<0.0001
		day 16	2	9.359	0.429	vs. 1. day P<0.0001
		day 20	2	13.123	1.095	vs. 1. day P<0.0001

TE/gene	Background	Trials	Relative 6mA levels	±S.E.M.	Independent two-sided *t*-test P value
for **Fig. 8a, b**					
Tc1	*wild-type* (control)	3	5701.9	1109.6	control
	wild-type (heat shock)	3	10446.6	1560.4	P<0.0001
Tc3	*wild-type* (control)	3	8129.0	2137.7	control
	wild-type (heat shock)	3	13286.6	3495.7	P=0.023

Supplementary Table 5. Statistics for the bioinformatical analysis of *Tc1* and *Tc3* transcript levels in wild-type versus *daf-2(-)* mutant genetic backgrounds.

Gene	Genotype	Trials	Relative mRNA level	±S.E.M.	Independent two-sided *t*-test P value
for **Supplementary Fig. 11a**					
CELE1	*wild-type*	6	16973.5	2676.176	control
	daf-2(-)	6	5932.5	906.2419	2.37E-06
CELE11	*wild-type*	6	628.3333	150.9777	control
	daf-2(-)	6	189.6667	88.72805	0.00011
CELE14A	*wild-type*	6	681	125.2629	control
	daf-2(-)	6	520.8333	144.2351	0.067093
CELE14B	*wild-type*	6	9304	2243.518	control
	daf-2(-)	6	6089.833	1119.215	0.010507
CELE2	*wild-type*	6	2076	302.717	control
	daf-2(-)	6	1026.167	237.579	5.48E-05
CELE4	*wild-type*	6	813	178.7199	control
	daf-2(-)	6	461.6667	144.5941	0.003824
CELE45	*wild-type*	6	1160.167	251.5301	control
	daf-2(-)	6	1814	102.1352	0.000151
CELE46B	*wild-type*	6	2880.167	560.3554	control
	daf-2(-)	6	1268.667	777.5543	0.002082
CELE7	*wild-type*	6	427.3333	71.74585	control
	daf-2(-)	6	245.5	75.79644	0.001643
CEMUDR2	*wild-type*	6	1463	466.7865	control
	daf-2(-)	6	534.3333	237.3105	0.001457
CER17-I_CE	*wild-type*	6	1588.833	529.7303	control
	daf-2(-)	6	465.5	320.457	0.001246
CER3-I_CE	*wild-type*	6	527.1667	79.09088	control
	daf-2(-)	6	301.5	130.8904	0.004733
CER9-I_CE	*wild-type*	6	2426.833	1047.674	control
	daf-2(-)	6	1219.5	527.1052	0.030303
CEREP1A	*wild-type*	6	7261.167	1573.184	control
	daf-2(-)	6	3599.167	1366.273	0.001549
CEREP53	*wild-type*	6	340	145.5005	control
	daf-2(-)	6	99.16667	108.853	0.008773
CERP1	*wild-type*	6	2141.667	559.3656	control
	daf-2(-)	6	3989	1135.87	0.005063
CERP4	*wild-type*	6	4933	944.6311	control
	daf-2(-)	6	2098.333	417.9443	5.22E-05
CHAPAEV-1_CE	*wild-type*	6	528.8333	212.4104	control
	daf-2(-)	6	323.1667	210.8406	0.123231
DNA-3-1_CE	*wild-type*	6	387.1667	120.3917	control
	daf-2(-)	6	208.3333	53.51511	0.007684

HAT1_CE	*wild-type*	6	3108.833	624.613	control
	daf-2(-)	6	1444	390.5714	0.000249
HAT2_CE	*wild-type*	6	1507.167	365.8122	control
	daf-2(-)	6	830.8333	258.0019	0.004103
IR1_CE	*wild-type*	6	715.6667	262.8008	control
	daf-2(-)	6	134	127.0889	0.000641
IR3_CE	*wild-type*	6	451.6667	151.7783	control
	daf-2(-)	6	130.6667	67.2478	0.000796
LINE2A_CE	*wild-type*	6	846.1667	274.8959	control
	daf-2(-)	6	485.5	90.65043	0.012208
LONGPAL1	*wild-type*	6	1894.667	712.9021	control
	daf-2(-)	6	822.3333	275.1019	0.006359
MARINCE1 (Tc14)	*wild-type*	6	103.5	73.53027	control
	daf-2(-)	6	197.6667	117.6685	0.127418
NDNAX2_CE	*wild-type*	6	15923.5	1915.557	control
	daf-2(-)	6	5192.833	1781.427	1.52E-06
NTC2A	*wild-type*	6	7642.5	3055.241	control
	daf-2(-)	6	2756.833	1761.235	0.006845
PAL3A_CE	*wild-type*	6	586.3333	213.9875	control
	daf-2(-)	6	177.3333	101.0261	0.001734
PAL5A_CE	*wild-type*	6	19477.5	3245.668	control
	daf-2(-)	6	7179.667	864.9466	4.27E-06
PAL8A_CE	*wild-type*	6	1902	401.2755	control
	daf-2(-)	6	577	171.5074	2.22E-05
PAL8C_5	*wild-type*	6	357.8333	226.3673	control
	daf-2(-)	6	83.16667	106.242	0.022679
PAL8D_CE	*wild-type*	6	7205.333	1601.604	control
	daf-2(-)	6	2731.833	1116.927	0.000224
PALNN1_CE	*wild-type*	6	794.6667	161.5694	control
	daf-2(-)	6	29.33333	39.65182	5.27E-07
PALTA3_CE	*wild-type*	6	858.8333	310.7284	control
	daf-2(-)	6	447.3333	235.0512	0.027086
PALTA4_CE	*wild-type*	6	411.1667	154.3093	control
	daf-2(-)	6	55.66667	35.43257	0.000262
PALTTAA1_CE	*wild-type*	6	5857.333	1266.045	control
	daf-2(-)	6	2256.833	1407.903	0.000897
PALTTAA3_CE	*wild-type*	6	9520.667	1486.797	control
	daf-2(-)	6	1697.167	624.5758	3.2E-07
PALTTTAAA1	*wild-type*	6	1026.833	249.1011	control
	daf-2(-)	6	598.6667	276.1932	0.018166
RCC9	*wild-type*	6	115.6667	35.17764	control
	daf-2(-)	6	51.83333	26.63394	0.005324
RTE-1	*wild-type*	6	8658.833	4041.09	control
	daf-2(-)	6	14043.83	5280.069	0.075391
TC1	*wild-type*	6	71650.5	14761.62	control

	daf-2(-)	6	14866.83	3803.455	3.66E-06
TC2	*wild-type*	6	1911	339.2474	control
	daf-2(-)	6	1086.167	423.9865	0.00397
TC3	*wild-type*	6	2215.333	428.2133	control
	daf-2(-)	6	1494.667	575.4861	0.033624
TC5A	*wild-type*	6	12306.83	2424.54	control
	daf-2(-)	6	2415	928.1502	2.98E-06
TC6	*wild-type*	6	7532.833	929.6783	control
	daf-2(-)	6	1076.333	504.301	3.6E-08
TURMOIL1	*wild-type*	6	9868.5	1489.621	control
	daf-2(-)	6	5152.5	1271.587	0.000151
for **Supplementary Fig. 11b**					
Tc1	*wild-type*	6	71650	14866	control
	daf-2(-)	6	14761	3803	0.002
for **Supplementary Fig. 11c**					
Tc3	*wild-type*	6	2215	1494	control
	daf-2(-)	6	428	575	0.0001
for **Supplementary Fig. 11d**					
Tc14	*wild-type*	6	1035	1976	control
	daf-2(-)	6	735	1176	0.05

Supplementary Table 6. Statistics for thermotolerance assays.

**Strain**	**Number of plates**	**Trials**	**Number of animals**	**Mean survival (%)**	**±S.D.**	**Independent two-sided t-test P value**
for **Fig. 8d**						
control RNAi	9	3	252	29	7	control
Tc1+Tc3(RNAi)	9	3	277	56	13	0.035
for **Fig. 8e**						
N2	3	3	71	54	13	control
damt-1(-)	3	3	88	87	0	0.0001
nmad-1(-)	3	3	53	38	8	0.127
daf-2(-)	3	3	82	96	14	0.0001
daf-2(-);nmad-1(-)	3	3	61	71	6	0.0089
for **Fig. 8e**						
N2	3	3	79	62	13	control
damt-1(-)	3	3	89	83	9	0.0019
nmad-1(-)	3	3	70	54	2	0.3421
daf-2(-)	3	3	90	96	2	0.0001

daf-2(-);nmad-1(-)	3	3	101	100	0	0.0001

Supplementary Table 7. Statistics for GFP expression levels.

Strain	Adult age (days)	Trials	Number of animals	Mean GFP intensity (a.u.)	±S.D.	Independent two-sided *t*-test P value
for **Supplementary Fig. 13d**						
control RNAi; sod-3::gfp	1	2	18	329.15	53.33	control
Tc1+Tc3(RNAi); sod-3::gfp	1	2	26	333.76	41.84	P = 0.764
control RNAi; sod-3::gfp	5	2	34	353.181	123.956	control
Tc1+Tc3(RNAi); sod-3::gfp	5	2	24	516.179	134.71	P = 0.0000141
control RNAi; sod-3::gfp	10	2	16	379.058	83.564	control
Tc1+Tc3(RNAi); sod-3::gfp	10	2	13	441.8126	108.482	P = 0.119
for **Supplementary Fig. 13b**						
control RNAi; hsp-16.2::gfp	1	2	30	4205.579	2694.063	control
Tc1+Tc3(RNAi); hsp-16.2::gfp	1	2	28	2776.168	1781.491	P = 0.020
control RNAi; hsp-16.2::gfp	5	2	27	871.669	262.415	control
Tc1+Tc3(RNAi); hsp-16.2::gfp	5	2	19	793.473	158.135	P = 0.215
control RNAi; hsp-16.2::gfp	10	2	12	526.239	118.719	control
Tc1+Tc3(RNAi); hsp-16.2::gfp	10	2	11	590.647	101.704	P = 0.273
for **Supplementary Fig. 13g**						
hsp-16.2::gfp	1	2	33	2831.582	1268.795	control
damt-1(-); hsp-16.2::gfp	1	2	39	2350.666	874.99	P = 0.071
nmad-1(-); hsp-16.2::gfp	1	2	28	2611.393	1030.223	P = 0.465
hsp-16.2::gfp	5	2	25	880.831	262.981	control
damt-1(-); hsp-16.2::gfp	5	2	18	708.508	314.403	P = 0.057
damt-1(-); hsp-16.2::gfp	5	2	27	735.495	326.917	P = 0.084
hsp-16.2::gfp	10	2	22	779.435	239.122	control
damt-1(-); hsp-16.2::gfp	10	2	16	761.876	122.644	P = 0.769

damt-1(-); hsp-16::gfp	10	2	25	721.949	229.549	P = 0.405
for **Supplementary Fig. 13f**						
control RNAi; gcs-1::gfp	1	1	30	0	0	control
damt-1 RNAi; gcs-1::gfp	1	1	30	0	0	NS
nmad-1 RNAi; gcs-1::gfp	1	1	30	0	0	NS
control RNAi; gcs-1::gfp	5	1	30	0	0	control
damt-1 RNAi; gcs-1::gfp	5	1	30	0	0	NS
nmad-1 RNAi; gcs-1::gfp	5	1	30	0	0	NS
control RNAi; gcs-1::gfp	10	1	30	0	0	control
damt-1 RNAi; gcs-1::gfp	10	1	30	0	0	NS
nmad-1 RNAi; gcs-1::gfp	10	1	30	0	0	NS
for **Supplementary Fig. 13h**						
control RNAi; gcs-1::gfp	1	1	30	0	0	control
Tc1+Tc3(RNAi); gcs-1::gfp	1	1	30	0	0	NS
control RNAi; gcs-1::gfp	5	1	30	0	0	control
Tc1+Tc3(RNAi); gcs-1::gfp	5	1	30	0	0	NS
control RNAi; gcs-1::gfp	10	1	30	0	0	control
Tc1+Tc3(RNAi); gcs-1::gfp	10	1	30	0	0	NS

Strain	Adult age (days)	Trials	Number of animals	Relative expression level	±S.E.M.	Kruskal-Wallis H test with Dunn's Post Hoc Test P value
for **Supplementary Fig. 15a, b, d**						
NMAD-1::GFP	1	3	121	1.000	0.022	control
	5	3	59	1.489	0.065	P<0.0001
	10	3	82	2.230	0.111	P<0.0001

Supplementary Table 8. Statistics for RNAseq.

Gene	Genotype	Trials	Relative mRNA levels	±S.D.	Independent two-sided *t*-test P value

for **Supplementary Fig. 14a**					
damt-1	*wild-type*	4	683.273	10.848	control
	daf-2(-)	4	585.187	35.662	0.0015
nmad-1	*wild-type*	4	482.577	23.787	control
	daf-2(-)	4	511.946	20.251	0.0346
for **Supplementary Fig. 14b**					
damt-1	*wild-type*	2	0.996744	0.011389	control
	daf-2(-)	2	0.971133	0.054699	0.58331
	daf-16(-);daf-2(-)	2	1.032122	0.000761	0.048312
nmad-1	*wild-type*	2	0.918759	0.011433	control
	daf-2(-)	2	1.145153	0.00116	0.001286
	daf-16(-);daf-2(-)	2	0.842448	0.016048	0.031755
