# Supplementary material for: Downregulation of transposable elements extends lifespan in Caenorhabditis elegans
Source: Nat Commun. 2023 Aug 29;14:5278. doi: 10.1038/s41467-023-40957-9 (PMC10465613; doi:10.1038/s41467-023-40957-9)
Supplement: Supplementary file 2 — Reporting Summary [file 41467_2023_40957_MOESM2_ESM.pdf]

nature portfolio

Corresponding author(s): Vellai Tibor

Last updated by author(s): May 22, 2023

Reporting Summary

Nature Portfolio wishes to improve the reproducibility of the work that we publish. This form provides structure for consistency and transparency in reporting. For further information on Nature Portfolio policies, see our Editorial Policies and the Editorial Policy Checklist.

Statistics

For all statistical analyses, confirm that the following items are present in the figure legend, table legend, main text, or Methods section.

n/a	Confirmed	
☐	☒	The exact sample size (*n*) for each experimental group/condition, given as a discrete number and unit of measurement
☐	☒	A statement on whether measurements were taken from distinct samples or whether the same sample was measured repeatedly
☐	☒	The statistical test(s) used AND whether they are one- or two-sided<br>*Only common tests should be described solely by name; describe more complex techniques in the Methods section.*
☒	☐	A description of all covariates tested
☐	☒	A description of any assumptions or corrections, such as tests of normality and adjustment for multiple comparisons
☐	☒	A full description of the statistical parameters including central tendency (e.g. means) or other basic estimates (e.g. regression coefficient) AND variation (e.g. standard deviation) or associated estimates of uncertainty (e.g. confidence intervals)
☐	☒	For null hypothesis testing, the test statistic (e.g. *F*, *t*, *r*) with confidence intervals, effect sizes, degrees of freedom and *P* value noted<br>*Give P values as exact values whenever suitable.*
☒	☐	For Bayesian analysis, information on the choice of priors and Markov chain Monte Carlo settings
☒	☐	For hierarchical and complex designs, identification of the appropriate level for tests and full reporting of outcomes
☒	☐	Estimates of effect sizes (e.g. Cohen's *d*, Pearson's *r*), indicating how they were calculated

Our web collection on statistics for biologists contains articles on many of the points above.

Software and code

Policy information about availability of computer code

Data collection	Statistical significance was determined by independent samples t tests (SPSS 17 778 software).
Data analysis	We used command line tools of PACBIO SMRT Link (version 7.0.1) for methylation calling. First PacBio sequences (stored in unaligned BAM files) were mapped to the C. elegans reference genome (Caenorhabditis_elegans.WBcel235.dna.toplevel.fa) by pbalign (version 0.4.1). Then, ipdSummary (version 2.4) was used on the aligned BAM files to detect DNA base modifications from kinetic signatures. The methylation levels of repeat sequences and cDNA sequences were calculated by in-house python scripts using the gff files, and the annotation files of Dfam release 3.1 (https://www.dfam.org/releases/733 Dfam_3.1/annotations/ce10/, https://doi.org/10.1093/nar/gkv1272).

For manuscripts utilizing custom algorithms or software that are central to the research but not yet described in published literature, software must be made available to editors and reviewers. We strongly encourage code deposition in a community repository (e.g. GitHub). See the Nature Portfolio guidelines for submitting code & software for further information.

Data

Policy information about availability of data

All manuscripts must include a data availability statement. This statement should provide the following information, where applicable:

- Accession codes, unique identifiers, or web links for publicly available datasets
- A description of any restrictions on data availability
- For clinical datasets or third party data, please ensure that the statement adheres to our policy

All data supporting the findings of this study are provided within the paper and its Supplementary information. Any data are available from the authors upon request. Raw SMRT-sequencing reads of the 1 day old and 5 days old animals are available in the NCBI Sequence Read Archive (SRA) database with the BioProject accession PRJNA68248. All data, code, and materials used in the analysis are available upon reasonable request for collaborative studies regulated by materials/data transfer agreements (MTA/DTAs) to the corresponding author (vellai.tibor@ttk.elte.hu). Uncropped versions of every picture used in the study can be downloaded from the link below: https://osf.io/c9wxj/?view_only=b67f2ae5eb9a4f9abbe0c5baa563dedb

Human research participants

Policy information about studies involving human research participants and Sex and Gender in Research.

Reporting on sex and gender	*Use the terms sex (biological attribute) and gender (shaped by social and cultural circumstances) carefully in order to avoid confusing both terms. Indicate if findings apply to only one sex or gender; describe whether sex and gender were considered in study design whether sex and/or gender was determined based on self-reporting or assigned and methods used. Provide in the source data disaggregated sex and gender data where this information has been collected, and consent has been obtained for sharing of individual-level data; provide overall numbers in this Reporting Summary. Please state if this information has not been collected. Report sex- and gender-based analyses where performed, justify reasons for lack of sex- and gender-based analysis.*
Population characteristics	*Describe the covariate-relevant population characteristics of the human research participants (e.g. age, genotypic information, past and current diagnosis and treatment categories). If you filled out the behavioural & social sciences study design questions and have nothing to add here, write "See above."*
Recruitment	*Describe how participants were recruited. Outline any potential self-selection bias or other biases that may be present and how these are likely to impact results.*
Ethics oversight	*Identify the organization(s) that approved the study protocol.*

Note that full information on the approval of the study protocol must also be provided in the manuscript.

Field-specific reporting

Please select the one below that is the best fit for your research. If you are not sure, read the appropriate sections before making your selection.

☒ Life sciences ☐ Behavioural & social sciences ☐ Ecological, evolutionary & environmental sciences

For a reference copy of the document with all sections, see nature.com/documents/nr-reporting-summary-flat.pdf

Life sciences study design

All studies must disclose on these points even when the disclosure is negative.

Sample size	For lifespan experiments n=2 was chosen as the minimal replicate number and n>50 was chosen as the minimal sample size. We determined this to be sufficient to reach statistical significance for all previously published results that we have repeated, so we assumed that it is sufficient for all new results as well.
Data exclusions	Data were not excluded from analysis.
Replication	All replication attempts were successful and observed patterns were consistent with orthogonal methods and previously known results.
Randomization	Every sample was analyzed equally with no sub-sampling and thus, there was no requirement for randomization.
Blinding	Blinding was not possible as experimental conditions were evident from the data. Quantifications were performed using a computational pipeline applied equally to all conditions and replicates for a given experiment.

Reporting for specific materials, systems and methods

We require information from authors about some types of materials, experimental systems and methods used in many studies. Here, indicate whether each material, system or method listed is relevant to your study. If you are not sure if a list item applies to your research, read the appropriate section before selecting a response.

Materials & experimental systems

n/a	Involved in the study
☒	☐ Antibodies
☒	☐ Eukaryotic cell lines
☒	☐ Palaeontology and archaeology
☐	☒ Animals and other organisms
☒	☐ Clinical data
☒	☐ Dual use research of concern

Methods

n/a	Involved in the study
☒	☐ ChIP-seq
☒	☐ Flow cytometry
☒	☐ MRI-based neuroimaging

Animals and other research organisms

Policy information about studies involving animals; ARRIVE guidelines recommended for reporting animal research, and Sex and Gender in Research

Laboratory animals	The following Caenorhabditis elegans strains were used in this study:<br>Bristol (N2) as wild-type<br>DR1344 Bergerac BO<br>CB1370 daf-2(e1370)III<br>TJ1060 spe-9(hc88)I; rrf-3(b26)II<br>VC2552 nmad-1(ok3133)III<br>VC40319 C18A3.1(gk961032)II<br>TTV680 unc-119(ed3)III; eluEx390[phsp-16.2::Tc3 + unc-119(+)]<br>TTV681 unc-119(ed3)III; eluEx391[phsp-16.2::prg-1 + unc-119(+)]<br>TTV614 unc-119(ed3)III; eluEx330[phsp-16.2::prg-1::gfp + unc-119(+)]<br>TTV491 unc-119(ed3)III; eluEx364[phsp-16.2::prg-2 + unc-119(+)]<br>TTV615 unc-119(ed3)III; eluEx358[phsp-16.2::ppw-2 + unc-119(+)]<br>TTV721 unc-119(ed3)III; eluIs314[pnmad-1::NMAD-1::GFP + unc-119(+)]<br>CF1553 muIs84[(pAD76) sod-3p::GFP + rol-6(su1006)]<br>TJ375 gpIs1[hsp-16.2::GFP]<br>TTV804 nmad-1(ok3133)III; gpIs1<br>TTV805 nmad-1(ok3133)III; muIs84<br>TTV806 C18A3.1(gk961032)II; gpIs1<br>TTV807 C18A3.1(gk961032)II; muIs84<br>Prior to performing lifespan assays, VC2552 and VC40319 strains were isogenized by outcrossing 3 times with the wild type.<br>All animals used were hermaphrodites.
Wild animals	No wild animals were used in this study.
Reporting on sex	All animals used were hermaphrodites.
Field-collected samples	No field-collected samples were used in this study.
Ethics oversight	There was no need for ethical approval and guidance as Caenorhabditis elegans and in vitro research do not require such.

Note that full information on the approval of the study protocol must also be provided in the manuscript.
